# Regio- and diastereoselective synthesis of thioxothiazolidin-indolin-2-ones, oxoindolin-carbamodithioate hybrids, and their base-catalyzed conversion into dispirocyclopentanebisoxindoles

**DOI:** 10.1038/s41598-024-65087-0

**Published:** 2024-06-20

**Authors:** Bagher Aghamiri, F. Matloubi Moghaddam, Sara Badpa, Leila Kavoosi

**Affiliations:** Laboratory of Organic Synthesis and Natural Products, Department of Chemistry, Sharif University of Technology, Azadi Street, PO Box 111559516, Tehran, Iran

**Keywords:** Regio- and diastereoselectivity, Kinetically controlled reaction, Thioxothiazolidin-indolin-2-ones, Dispirocyclopentanebisoxindoles, Oxoindolin-carbamodithioate hybrids, Drug discovery, Chemistry

## Abstract

In this publication, we reported a regio-, diastereoselective, and kinetically controlled reaction for synthesizing thioxothiazolidin-indolin-2-ones, oxoindolin-carbamodithioate hybrids, and their base-catalyzed conversion into dispirocyclopentanebisoxindoles. Obtaining only the kinetic thioxothiazolidin-indolin-2-one products together with their straightforward conversion to dispirocyclopentanebisoxindoles, excellent regio- and diastereoselectivity, easy reaction workup, and one-pot synthetic operation are considerable advantages of this work.

## Introduction

3-Alkenyl-oxindoles are pharmacologically advantageous scaffolds that have many biological properties. The widespread occurrence of oxindole cores at the heart of many plant-based alkaloids and natural products has further reinforced their merit in organic and medicinal chemistry^1–5^. In recent decades, 3-alkenyl oxindoles have been widely investigated, and detailed works have been done for transforming 3-alkenyl oxindoles into novel functionalized oxindole-based heterocycles^6–10^. For instance, very recently, Li et al. described a procedure for the construction of sp^2^ C–N bond between 3-alkenyl oxindoles and indazole/benzotriazole^11^. In 2023, Mainkar et al. reported proline-catalyzed diastereoselective synthesis of dihydroquinolinyl-spirooxindole via aza-Michael/aldol reaction of 3-alkenyl oxindole^12^. In 2023, Song and coworkers described the synthesis of 3-alkenyl-2-oxindoles via a transition-metal-free [4+1] cyclization pathway^13^. Again in 2023, Stephan et al. developed a metal-free, B(C_6_F_5_)_3_ catalyzed cyclopropanation of 3-alkenyl-oxindoles with diazomethanes^14^. Copper-catalyzed hydroboration of these compounds was also reported by Moro et al.^15^. According to other reports, 3-alkenyl oxindoles could easily converted to dispirooxindoles^16–21^. For example, the synthesis of dispirocyclopentanebisoxindoles via sequential Michael-aldol reaction of 3-alkenyl oxindoles was reported by Yan et al.^22^. In addition, very recently our research group described a facile reaction for transforming 3-alkenyl oxindoles to dispirocyclopentanebisoxindoles^23^.

**Scheme 1:**
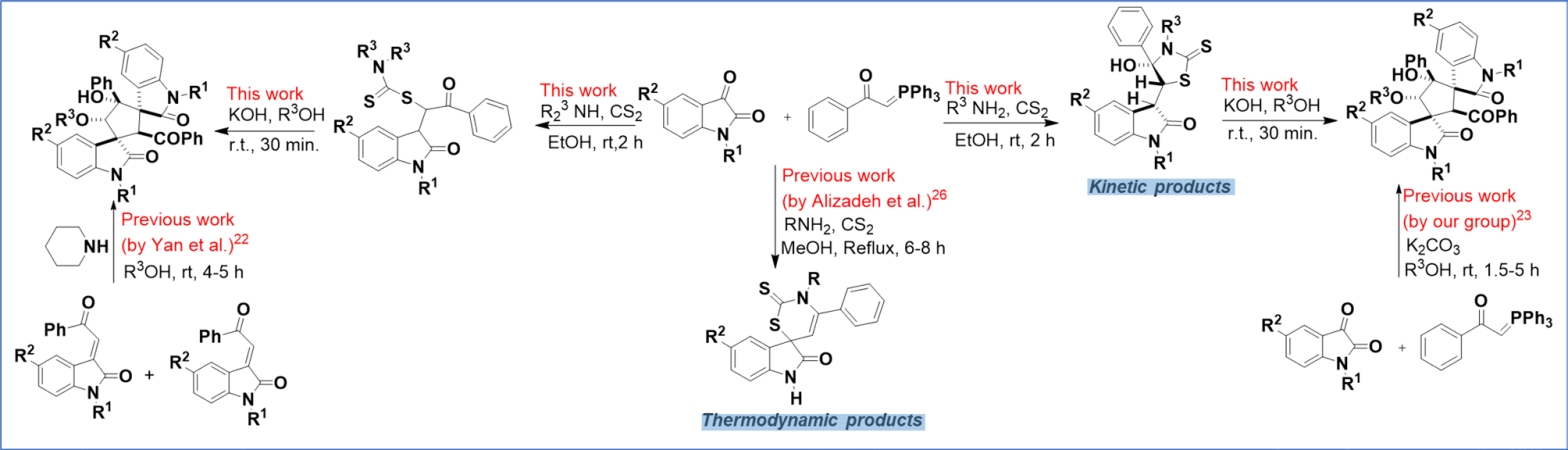
A comparative overview of the employed synthetic process in the previous and this work. All of the images were generated using ChemDraw 2016 (https://chemistry.com.pk/software/chemdraw-pro-2016/#google_vignette), PowerPoint 2016 (https://www.microsoft.com/en-us/store/collections/getpowerpoint2016andmorewithoffice365), and paint (https://apps.microsoft.com/detail/9pcfs5b6t72h?hl=en-US&gl=US)

On the other hand, from the distant past to the present, S-heterocycles have maintained their importance as an essential part and core of FDA-approved drugs and medicinally active molecules. A straightforward approach to synthesizing S-heterocycles is exploiting dithiocarbamates^24,25^. The great nucleophilic strength of dithiocarbamates has made them widely used in organic transformations. For example, Alizadeh and coworkers reported a thermodynamic approach for synthesizing 2ʹ,3ʹ-dihydro-2ʹ-thioxospiro[indole-3,6ʹ-[1, 3]-thiazin]-2(1*H*)-ones derivatives using dithiocarbamates at reflux conditions^26^. Unexpectedly, when we checked the above reaction at room temperature, we found that the final products are completely different from the previous work, and at room temperature, the reaction seems to be under kinetic control that leads to the formation of novel thioxothiazolidin-indolin-2-ones molecules (compounds **3a–3x**). In addition to this, we found that the final products (thioxothiazolidin-indolin-2-ones (compounds **3a–3x**) and oxoindolin-carbamodithioates (compounds **4a–4d**)) are not stable in basic media, and easily convert to dispirocyclopentanebisoxindoles under basic conditions, the same as 3-alkenyl oxindoles and our previous work (Scheme 1).

As a result of what we have mentioned above, in this publication, we would like to report a kinetically controlled reaction for synthesizing novel thioxothiazolidin-indolin-2-ones, oxoindolin-carbamodithioate hybrids, and their base-catalyzed conversion to dispirocyclopentanebisoxindoles. This synthetic procedure provides a feasible approach for synthesizing novel organic molecules bearing oxindole cores that might have future medicinal applications and the obtained molecules could be suitable for drug screening.

## Results and discussion

Initially, we investigated the four-component reaction of *N*-allyl isatin **1a**, phosphonium ylide **2**, carbon disulfide, and methylamine as the model reaction (Table 1). Thus, a mixture of **1** (0.3 mmol), **2**(0.3 mmol) in EtOH (2.0 mL) was mixed and stirred at room temperature for 30 min, then methylamine (0.3 mmol) and carbon disulfide (0.3 mmol) were added and stirred for more 1.5 h to afford 1-allyl-4-hydroxy-3-methyl-4-phenyl-2-thioxothi-azolidin-5-yl)indolin-2-one (**3a**) in satisfactory yield. Then, several solvents were assessed to achieve the optimal reaction conditions. The reaction proceeded slowly in DMSO, CHCl_3_, CH_3_CN, and CH_2_Cl_2_ with unsatisfactory yield (Table 1, entries 1–4). Furthermore, the reaction failed to occur in H_2_O and THF (Table 1, entries 5, 6). In complete contrast, the reaction yield increased greatly in alcoholic solvents such as *n*-Octanol, MeOH, and PrOH, especially in EtOH (Table 1, entries 7–12). PEG-400 was not as good as alcoholic solvents (Table 1, entry 10). We found that the reaction proceeded well under catalyst-free conditions and at room temperature. These results prompted us to further screen the reaction conditions. In the next step of synthetic work, we considered the formation of product **3a** under reflux conditions (Table 1, entry 12). We found that reflux conditions prevent product **3a** formation (please refer to Scheme 1). In addition, product **3a** was also sensitive to heat. If we heat it for a short time (less than 1 min.), this compound returns to isatin chalcone (The color change from white to orange).

**Table 1:** Screening the optimal reaction conditions.


Entry^a^	solvent	Base (equiv.)	Amines	Temp. (°C)	Time	Yield^b^ of **3a** (%)	Yield^b^ of **4a** (%)	Yield^b^ of **5** (%)
1	DMSO	–	MeNH_2_	25	2h	36	–	–
2	CHCl3	–	MeNH_2_	25	2h	53	–	–
3	CH_3_CN	–	MeNH_2_	25	2h	42	–	–
4	CH_2_Cl_2_	–	MeNH_2_	25	2h	48	–	–
5	H_2_O	–	MeNH_2_	25	2h	–	–	–
6	THF	–	MeNH_2_	25	2h	–	–	–
7	n-Octanol	–	MeNH_2_	25	2h	73	–	–
8	MeOH	–	MeNH_2_	25	2h	79	–	–
9	PrOH	–	MeNH_2_	25	2h	75	–	–
10	PEG-400	–	MeNH_2_	25	2h	56	–	–
11^c^	EtOH	–	MeNH_2_	25	2h	81	–	–
12	EtOH		MeNH_2_	Reflux	6h	–	–	–
13^c^	EtOH	–	Me2NH	25	2h	–	83	–
14	MeOH	–	Me_2_NH	25	2h	–	74	–
15	MeOH	KOH (0.8 equiv.)	MeNH_2_	25	30 min	–	–	86 (5a)
16	MeOH	KOH (0.3 equiv.)	MeNH_2_	25	30 min	–	–	71 (5a)
17	MeOH	KOH (excess)	MeNH_2_	25	30 min	–	–	85 (5a)
18	MeOH	NOH (0.8 equiv.)	MeNH_2_	25	30 min	–	–	75 (5a)
19	MeOH	K_2_CO_3_ (excess)	MeNH_2_	25	1 h	–	–	79 (5a)
20	MeOH	KOH (0.8equiv.)	Me_2_NH	25	30 min	–	–	79 (5a)
22	MeOH	LDA (0.8)	MeNH_2_	25	30 min	–	–	81 (5a)
23	*n*-Octanol	KOH (0.8 equiv.)	MeNH_2_	25	1.5 h	–	–	89 (5b)
24	PrOH	KOH (0.8 equiv.)	MeNH_2_	25	30 min	–	–	79 (5c)
25	PrOH	KOH (0.8 equiv.)	MeNH_2_	25	30 min	–	–	75 (5d)
26	MeOH	Et_3_N (excess)	MeNH_2_	25	24 h	–	–	–

^a^Reaction conditions: Reaction of 1a (0.3 mmol), 2 (0.3 mmol), carbon disulfide (0.3 mmol) and methylamine (0.3 mmol) was performed in 2.0 mL of solvent to form 3a product. Reaction of 1a (0.3 mmol), 2 (0.3 mmol), carbon disulfide (0.3 mmol) and dimethylamine (0.3 mmol) was performed in 2.0 mL of solvent to form 4a product. Reaction of 1a (0.3 mmol), 2 (0.3 mmol), carbon disulfide (0.3 mmol) and methylamine(0.3 mmol) was performed in 2.0 mL of alcoholic solvents and after 2 h of stirring, base was added to the mixture and stirred for more 30–90 min to form 5a-5d products.

^b^Isolated yields of products after filtering of reaction mixture. Simple filtering of the reaction mixture and washing with EtOH afforded desired products.

^c^Optimal reaction conditions.

**Figure 1:**
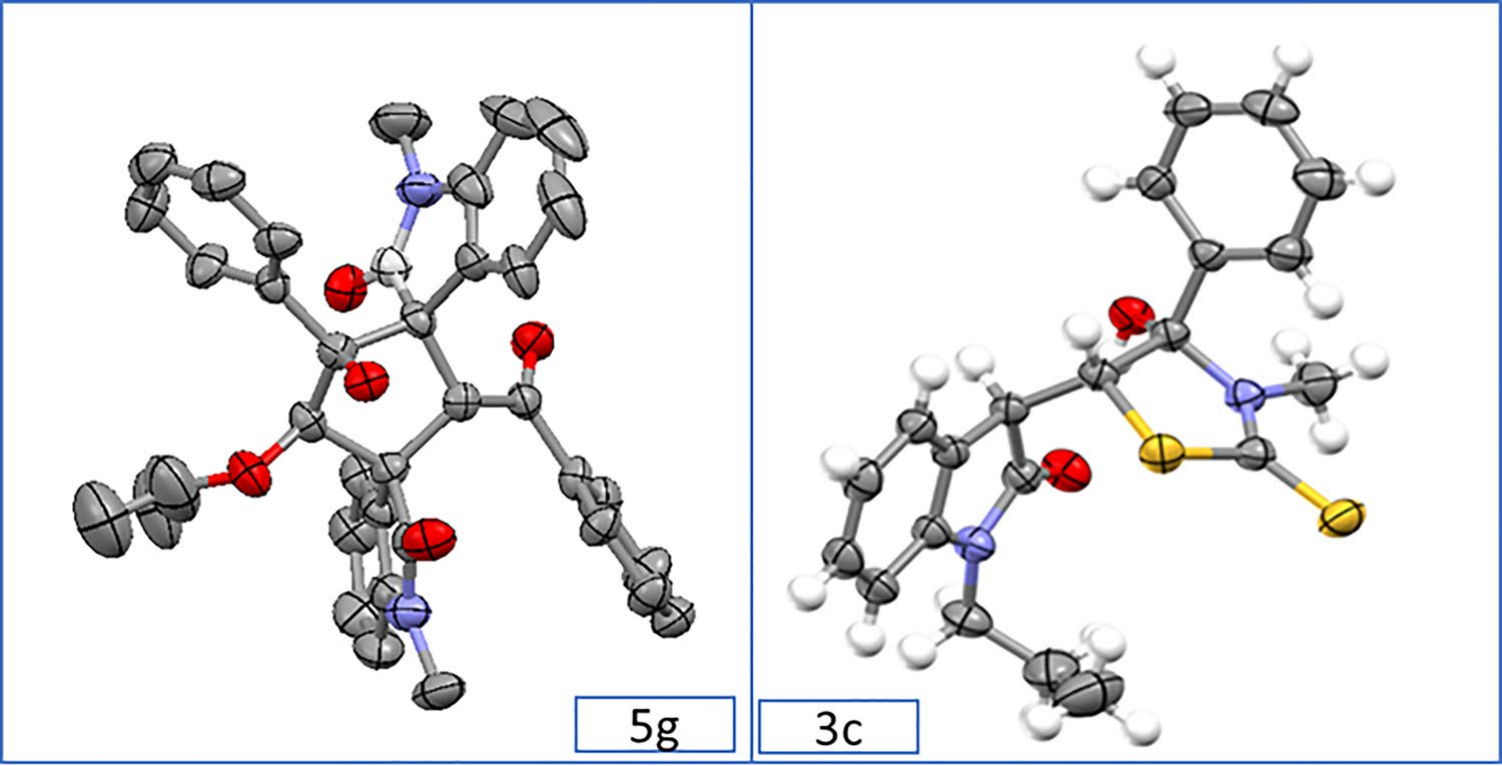
Single-crystal structure of 3c and 5g.

**Figure 2:**
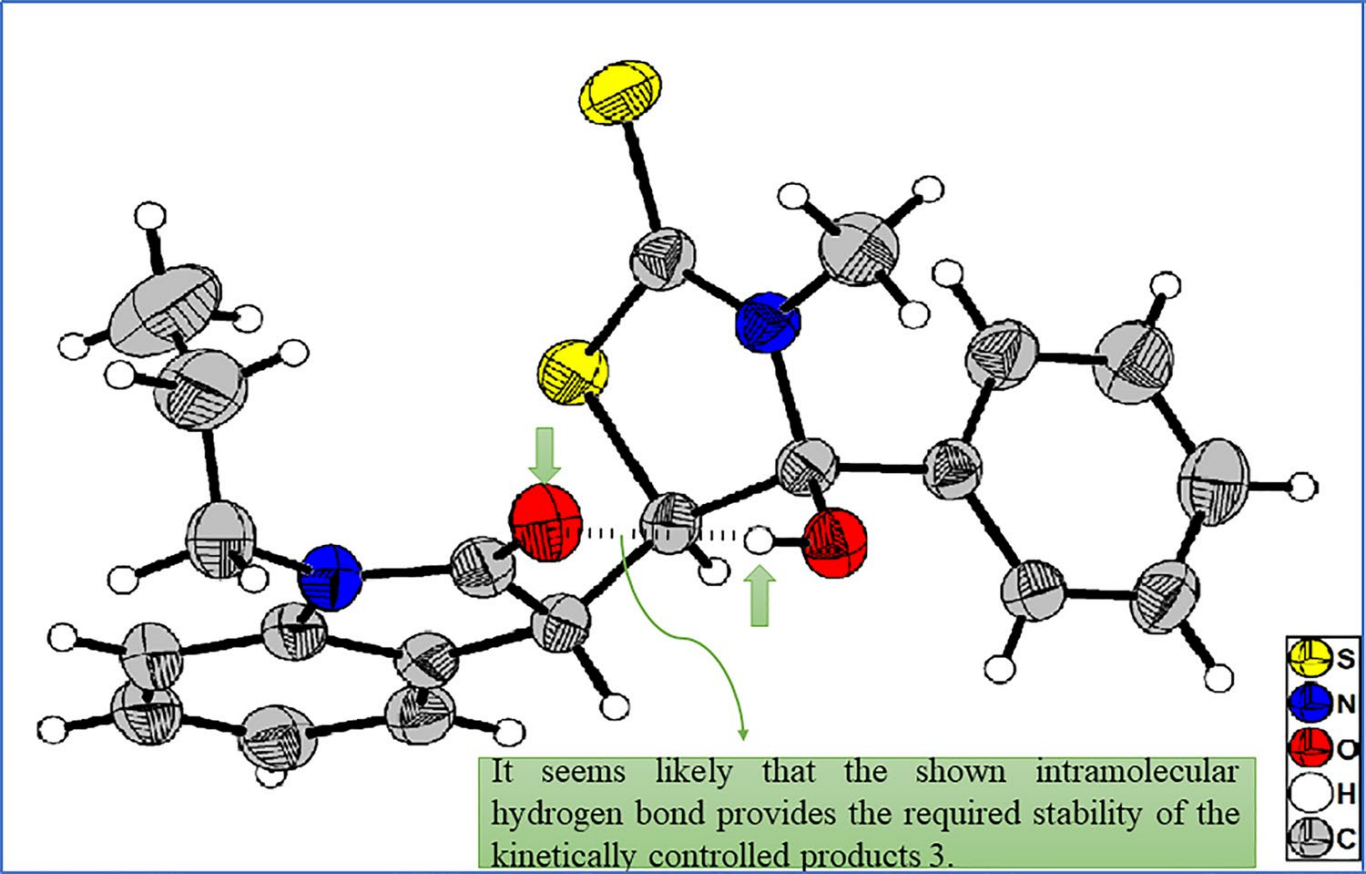
Possible explanation for the stability of the kinetic products 3a–x.

Hence, the best results for synthesizing product **3a** were obtained at 25 °C under catalyst-free conditions and after 2 h of stirring (Table 1, entry 11). The general procedure used for synthesizing product **3a** was also employed for compound **4a**, except using dimethylamine in place of methylamine (Table 1, entry 13). To our surprise, products **3a** and **4a** were easily converted to dispirocyclopentanebisoxindoles (**5a–5d**) under basic conditions (Table 1, entries 15–25). This conversion proceeded well in the presence of different bases such as LDA, KOH, NaOH, and K_2_CO_3_ via one-pot operation after 30–90 min of stirring. In contrast, Et_3_N converted products **3a** and **4a** to isatin chalcone and was not able to convert **3a** and **4a** to dispirocyclopentanebisoxindole products (Table 1, entry 26). In the next step of synthetic work, with the optimal reaction conditions in hand, the generality for substrates was also studied (Table 2). Most functional groups were tolerable and the reaction was carried out successfully with various substituents on the nitrogen and the aromatic ring of the isatin. Moreover, the reaction gave satisfactory answers with different aromatic and aliphatic amines. ^1^H and ^13^C NMR, FT-IR, and elemental analysis determined the structure of the final products. Compound 3c and 5g were also crystallized from EtOAc by slow evaporation at room temperature and the structure was confirmed by *X*-ray crystallographic analysis (Fig. 1). *X*-ray crystallographic structure of product **3c** show an intramolecular hydrogen bond that could be the main reason for the stability of the kinetic products (Fig. 2).

**Table 2:**
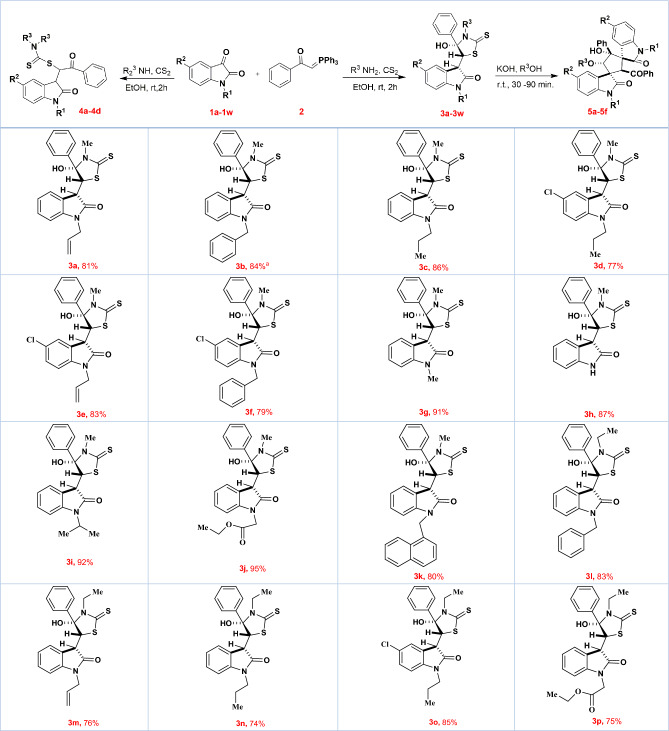

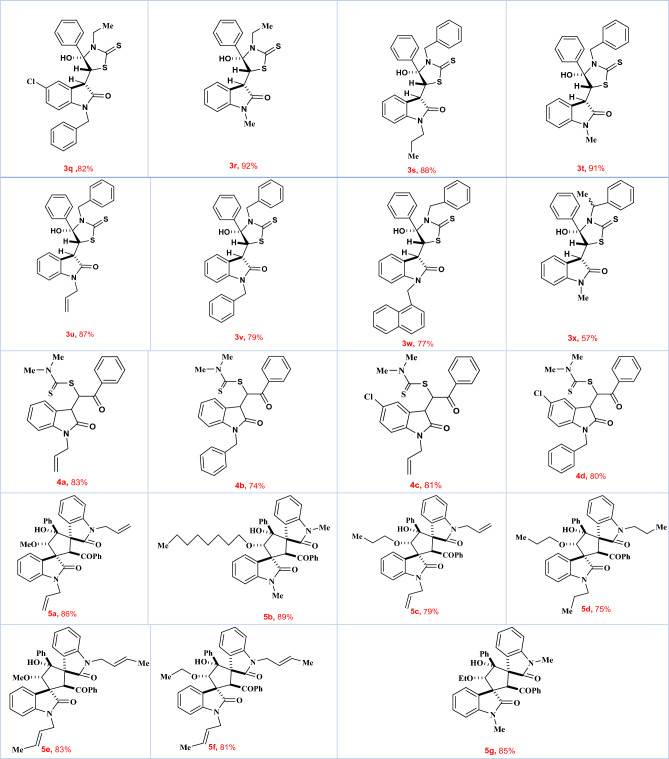
Generality of substrate scope.

Reaction conditions: Reaction of **1a–1w** (0.3 mmol), 2 (0.3 mmol), carbon disulfide (0.3 mmol) and primary amines (0.3 mmol) was performed in 2.0 mL of solvent to form **3a–3 × **products. Reaction of 1a–1d (0.3 mmol), 2 (0.3 mmol), carbon disulfide (0.3 mmol) and secondary amines (0.3 mmol) was performed in 2.0 mL of solvent to form **4a–4d** products. Reaction of 1a (0.3 mmol), 2 (0.3 mmol), carbon disulfide (0.3 mmol) and primary amines (0.3 mmol) was performed in 2.0 mL of alcoholic solvents and after 2 h of stirring, KOH was added to the mixture and stirred for more 30–90 min to form **5a–5 g** products (**5a**, **5c**, **5d**, **5e**, **5f** and **5g** were obtained after 30 min of stirring).

**Scheme 2:**
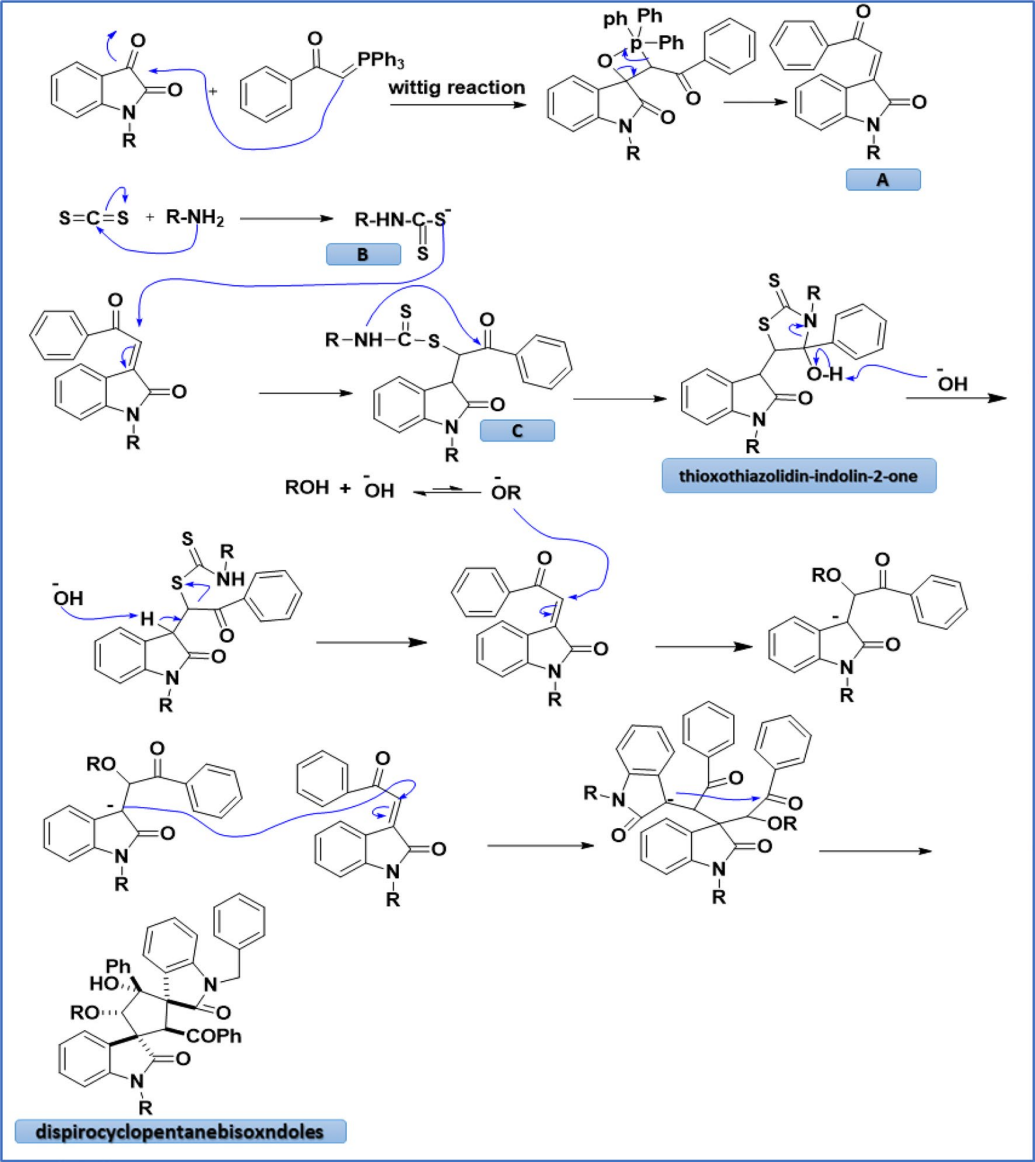
Proposed mechanism for the formation of final products.

To understand the sequence of steps of the reaction, we proposed a plausible mechanism as illustrated in Scheme 2. In the beginning, isatin derivatives reacted with phosphonium ylide to form isatin chalcone **A**. In addition, carbamodithioic acid **B** could be resulted from the addition of the amine to carbon disulfide. Subsequent attack of the carbamodithioic acid **B** on isatin chalcone **A** lead to the formation of intermediate **C**. Finally, the intramolecular attack on the carbonyl yields the final thioxothiazolidin-indolin-2-one products. Following, in basic media, the thioxothiazolidin-indolin-2-one could be converted to dispirocyclopentanebisoxindoles via sequential condensation, Michael addition, and intramolecular cyclization reaction.

**Scheme 3:**
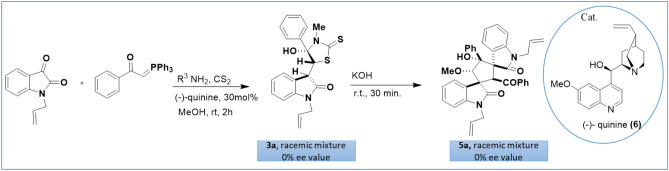
Study on catalytic asymmetric induction.

The products **3a–3x** and **5a–5g** were racemic mixtures (0% ee value) owing to the lack of chiral inducing agents. To obtain the related enantiomeric products in unequal amounts, we used the (−)-quinine (**6**, 30 mol%) as a chiral catalyst. Unfortunately, all our attempts to induce enantioselectivity and obtain the asymmetric version of the final products failed (Scheme 3). At the second attempt, we used chiral amine to induce enantioselectivity. For this reason, we checked the reaction using (S)-(−)-1-Phenylethylamine and obtained the product **3x**. Then, we determined the optical rotation of compound **3x.** To our surprise, although (S)-(−)-1-Phenylethylamine rotated the polarized light counterclockwise, compound **3x **was optical inactive and had the observed rotation of zero. It seems that racemization of the chiral amine occurred during the reaction pathway (please refer to Scheme 5).

**Scheme 4:**
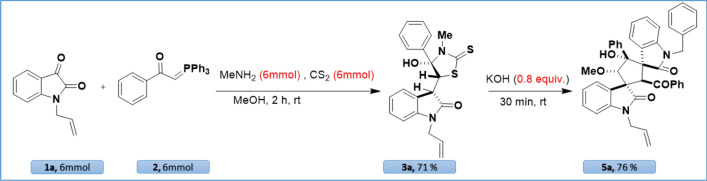
Scale-up reaction.

**Scheme 5:**
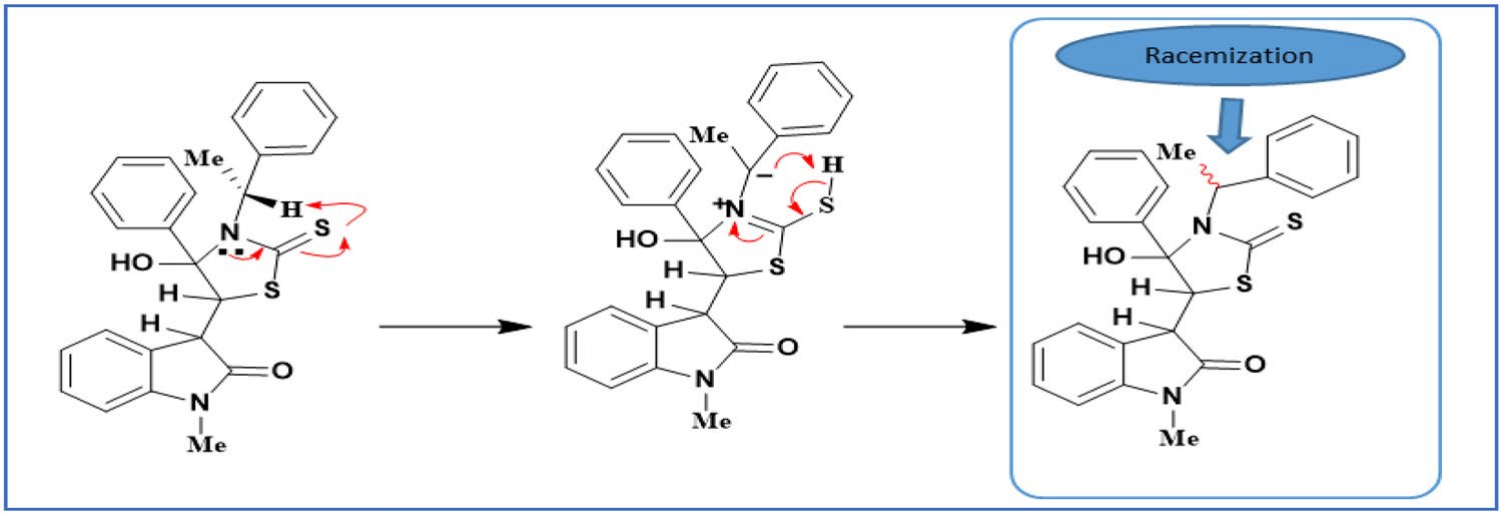
Proposed mechanism for racemization of the (S)-(−)-1-Phenylethylamine.

To show that these synthetic procedures are certainly worthwhile, we eventually tested the gram scale synthesis of products **3a** and **5a** (Scheme 4). For this reason, to a solution of *N-*allyl isatin (**1a**, 6.0 mmol, 1.12 g) in MeOH, phosphonium ylide (**2,** 6.0 mmol, 1.53 g) was added and stirred at room temperature for 30 min to afford the corresponding isatin chalcone. In the next step, carbon disulfide (6.0 mmol, 0.36 mL) and methylamine (6.0 mmol, 0.52 mL) were added to the reaction mixture and stirred for more 1.5 h to form product **3a**. Upon completion of the reaction, the reaction color changes from red to pale yellow. In this stage, we added KOH (0.8 equiv.) to form dispirocyclopentanebisoxindole (**5a**). After completion of the reaction, the organic products were simply filtered off and the precipitate was washed with EtOH (Scheme 5).

We were delighted to obtain the products **3a** and **5a** in 71 and 76% yield, respectively.

## Conclusion

In this publication, we achieved success in designing regio-, diastereoselective, and kinetically controlled reactions for synthesizing novel thioxothiazolidin-indolin-2-ones, oxoindolin-carbamodihioates, together with their conversion to dispirocyclopentanebisoxindoles in basic media. The procedure exhibited great efficiency in providing final products with decent yields from accessible starting materials and displaying excellent regio- and diastereoselectivity. Short reaction time, mild reaction conditions, satisfying the green chemistry standards, easy reaction workup, favorable response to gram-scale synthesis, and one-pot synthetic operation are other advantages of this work.

## Experimental section

### General remarks

All solvents and starting materials were purchased from Merck and Sigma-Aldrich used without any additional purification. Analytical TLC was carried out using Merck 0.2 mm silica gel 60 F-254 Al-plates. ^1^H NMR and ^13^C NMR spectra were recorded on a Bruker Avance DRX-500 machine using DMSO-*d*_6_ as solvent and TMS as an internal standard at room temperature (DMSO-*d*_6_^1^H NMR: *δ* (ppm) = 2.50 ppm; ^13^C NMR: *δ* (ppm) = 39.9 ppm; CDCl_3_^1^HNMR: *δ* (ppm) = 7.26 and ^13^C-NMR: *δ* (ppm) = 77.00 ppm). Chemical shifts were reported in ppm scale. FT-IR spectra of samples were obtained on ABB Bomem MB100 spectrometer with potassium bromide (KBr) pellets. Melting points were determined using an Electrothermal 9100 apparatus and are uncorrected. Elemental analysis was done by LECO Truspec.

### Experimental procedure for the synthesis of compounds 3 (3a–3x), 4 (4a–4d), and 5 (5a–5g)

To a solution of isatin derivatives (**1a–1x**, 0.3mmol) in alcoholic solvents (2.0 mL), phosphonium ylide (**2,** 0.3 mmol) was added and stirred at room temperature for 30 min to afford the corresponding isatin chalcone. Upon completion of the reaction, the reaction color changes from orange to red. In the next step, carbon disulfide (0.3 mmol) and primary (1°) amines (0.3 mmol) were added to the reaction mixture and stirred for more 1.5 h to form products **3a–3x** (the general procedure used for synthesizing the products **3a–3x **was also employed for compounds **4a–4d**, except using secondary (2°) amines in place of primary (1°) amines). Upon completion of the reaction, the reaction color changes from red to pale yellow. In this stage, if we added KOH (15 mg, 0.8 equiv.) to the reaction mixture, products **3** or **4** easily converted to dispirocyclopentane-bisoxindoles (**5a–5g**) after 30–90 min of stirring (first, the reaction color changes from pale yellow to red, and then from red to white). After completion of the reaction, the organic products were simply filtered off and the precipitate was washed with EtOH. The pure products were dried in air and directly characterized by ^1^H NMR, ^13^C NMR, elemental and FT-IR analysis. In addition, the structure of compound **3c** was confirmed by *X*-ray crystallographic analysis.

#### 1-Allyl-3-(4-hydroxy-3-methyl-4-phenyl-2-thioxothiazolidin-5-yl) indolin-2-one (3a)

White solid; yield (96 mg, 81%); m.p. 178–180 °C; ^1^H NMR (500 MHz, CDCl_3_) δ 3.09 (3H, s), 4.35 (1H, d.d., *J* = 5.0 Hz), 4.44 (1H, d, *J* = 5.0 Hz), 4.46 (1H, d, *J* = 5.0 Hz), 4.53 (1H, d.d, *J* = 5.0 Hz), 5.33 (2H, quintet, *J* = 15.0 Hz), 5.87 (1H, m), 6.93 (1H, d, *J* = 10.0 Hz), 7.08 (2H, t, *J* = 5.0 Hz), 7.31–7.59 (6H, m), 8.15 (1H, s) ppm; ^13^C NMR (125 MHz, CDCl_3_) δ 32.7, 43.2, 46.9, 56.8, 100.2, 110.2, 118.6, 123.7, 123.8, 124.8, 125.4, 129.2, 129.3, 129.4, 130.2, 141.8, 143.5, 176.5, 191.4 ppm; IR (KBr) ν = 3300–3080, 1675, 1640, 1609 cm^−1^; Anal. Calcd. for C_21_H_20_N_2_O_2_S_2_: C, 63.61; H, 5.08; N, 7.06; Found: C, 63.45; H, 5.16; N, 6.75%.

#### 1-Benzyl-3-(4-hydroxy-3-methyl-4-phenyl-2-thioxothiazolidin-5-yl) indolin-2-one (3b)

White solid; yield (112 mg, 84%); m.p. 160–162 °C; ^1^H NMR (500 MHz, CDCl_3_) δ 3.12 (3H, s), 4.46 (1H, d, *J* = 5.0 Hz), 4.52 (1H, d, *J* = 5.0 Hz), 4.79 (1H, d, *J* = 10.0 Hz), 5.23 (1H, d, *J* = 10.0 Hz), 6.81 (1H, d, *J* = 10.0 Hz), 7.05 (2H, t, *J* = 5.0 Hz), 7.23–7.62 (11H, m), 8.19 (1H, s) ppm; ^13^C NMR (125 MHz, CDCl_3_) δ 32.7, 44.8, 47.0, 56.9, 100.2, 110.4, 123.7, 123.8, 124.7, 125.4, 127.6, 128.1, 129.1, 129.2, 129.3, 129.4, 134.5, 141.8, 143.5, 176.9, 191.4 ppm; IR (KBr) ν = 3300–3161, 1675, 1614 cm^−1^; Anal. Calcd. for C_25_H_22_N_2_O_2_S_2_: C, 67.24; H, 4.97; N, 6.27; Found: C, 67.01; H, 5.11; N, 6.08%.

#### 3-(4-Hydroxy-3-methyl-4-phenyl-2-thioxothiazolidin-5-yl)-1-propylindolin-2-one (3c)

White solid; yield (102 mg, 86%); m.p. 172–174 °C; ^1^H NMR (500 MHz, CDCl_3_) δ 1.04 (3H, t,* J* = 10.0 Hz), 1.78 (2H, m), 3.09 (3H, s), 3.69 (1H, m), 3.86 (1H, m), 4.42 (1H, d, *J* = 5.0 Hz), 4.43 (1H, d, *J* = 5.0 Hz), 6.96 (1H, d, *J* = 10.0 Hz), 7.08 (2H, t, *J* = 5.0 Hz), 7.34–7.60 (6H, m), 8.27 (1H, s) ppm; ^13^C NMR (125 MHz, CDCl_3_) δ 11.6, 20.8, 32.7, 42.6, 46.8, 56.8, 100.2, 109.6, 123.6, 123.8, 124.7, 124.8, 125.6, 129.2, 129.4, 141.9, 143.9, 176.7, 191.4 ppm; IR (KBr) ν = 3300–3050, 1670, 1609 cm^−1^; Anal. Calcd. for C_21_H_22_N_2_O_2_S_2_: C, 63.29; H, 5.56; N, 7.03; Found: C, 62.97; H, 5.68; N, 6.87%.

#### 5-Chloro-3-(4-hydroxy-3-methyl-4-phenyl-2-thioxothiazolidin-5-yl)-1-propylindolin-2-one (3d)

White solid; yield (100 mg, 77%); m.p. 150–152 °C; ^1^H NMR (500 MHz, CDCl_3_) δ 1.03 (3H, t,* J* = 5.0 Hz), 1.76 (2H, m), 3.08 (3H, s), 3.66 (1H, m), 3.84 (1H, m), 4.37 (1H, d, *J* = 5.0 Hz), 4.42 (1H, d, *J* = 5.0 Hz), 6.88 (1H, d, *J* = 10.0 Hz), 7.08 (1H, s), 7.33–7.57 (6H, m), 8.06 (1H, s) ppm; ^13^C NMR (125 MHz, CDCl_3_) δ 11.5, 20.8, 32.7, 42.8, 46.9, 56.6, 100.1, 100.2, 110.5, 124.3, 124.7, 127.2, 129.3, 129.4, 129.5, 141.6, 142.5, 176.2, 191.1 ppm; IR (KBr) ν = 3300–3056, 1675, 1609 cm^−1^; Anal. Calcd. for C_21_H_21_ClN_2_O_2_S_2_: C, 58.25; H, 4.89; N, 6.47; Found: C, 58.01; H, 5.04; N, 6.21%.

#### 1-Allyl-5-chloro-3-(4-hydroxy-3-methyl-4-phenyl-2-thioxothiazolidin-5-yl) indolin-2-one (3e)

White solid; yield (107 mg, 83%); m.p. 154–156 °C; ^1^H NMR (500 MHz, CDCl_3_) δ 3.08 (3H, s), 4.32 (1H, d.d, *J* = 5.0 Hz), 4.39 (1H, d, *J* = 5.0 Hz), 4.47 (1H, d, *J* = 5.0 Hz), 4.54 (1H, d.d, *J* = 5.0 Hz), 5.33 (2H, quintet, *J* = 15.0 Hz), 5.84 (1H, m), 6.86 (1H, d, *J* = 10.0 Hz), 7.09 (1H, s), 7.31–7.58 (6H, m), 7.95 (1H, s) ppm; ^13^C NMR (125 MHz, CDCl_3_) δ 32.8, 43.3, 46.9, 56.6, 100.2, 111.2, 118.9, 124.3, 124.7, 127.1, 129.3, 129.4, 129.9, 141.5, 156.7, 176.5, 191.0 ppm; IR (KBr) ν = 3300–3086, 1672, 1643, 1612 cm^−1^; Anal. Calcd. for C_21_H_19_ClN_2_O_2_S_2_: C, 58.53; H, 4.44; N, 6.50; Found: C, 58.22; H, 4.51; N, 6.35%.

#### 1-Benzyl-5-chloro-3-(4-hydroxy-3-methyl-4-phenyl-2-thioxothiazolidin-5-yl) indolin-2-one (3f)

White solid; yield (114 mg, 79%); m.p. 128–130 °C; ^1^H NMR (500 MHz, CDCl_3_) δ 3.12 (3H, s), 4.41 (1H, d, *J* = 5.0 Hz), 4.54 (1H, d, *J* = 5.0 Hz), 4.75 (1H, d, *J* = 10.0 Hz), 5.23 (1H, d, *J* = 10.0 Hz), 6.73 (1H, d, *J* = 10.0 Hz), 7.03 (1H, s), 7.21–7.60(11H, m), 7.99 (1H, s) ppm; ^13^C NMR (125 MHz, CDCl_3_) δ 32.8, 44.9, 47.1, 56.7, 100.2, 111.3, 124.2, 124.7, 127.1, 127.6, 128.3, 129.2, 129.3, 129.4, 129.5, 129.5, 134.0, 141.5, 142.0, 176.5, 191.0 ppm; IR (KBr) ν = 3350–3100, 1678, 1615 cm^−1^; Anal. Calcd. for C_25_H_21_ClN_2_O_2_S_2_: C, 62.42; H, 4.40; N, 5.82; Found: C, 62.15; H, 4.53; N, 5.65%.

#### 3-(4-Hydroxy-3-methyl-4-phenyl-2-thioxothiazolidin-5-yl)-1-methylindolin-2-one (3g)

White solid; yield (101 mg, 91%); m.p. 170–172 °C; ^1^H NMR (500 MHz, CDCl_3_) δ 3.09 (3H, s), 3.33 (3H, s), 4.43 (1H, d, *J* = 5.0 Hz), 4.45 (1H, d, *J* = 5.0 Hz), 6.94 (1H, d, *J* = 10.0 Hz), 7.09 (2H, t, *J* = 5.0 Hz), 7.36–7.59 (6H, m), 8.26 (1H, s) ppm; ^13^C NMR (125 MHz, CDCl_3_) δ 27.0, 32.8, 46.9, 56.7, 100.2, 109.3, 123.6, 123.9, 124.7, 125.3, 129.2, 129.3, 129.5, 142.0, 144.3, 176.6, 191.5 ppm; IR (KBr) ν = 3400–3150, 1676, 1604 cm^−1^; Anal. Calcd. for C_19_H_18_N_2_O_2_S_2_: C, 61.60; H, 4.90; N, 7.56; Found: C, 61.35; H, 4.96; N, 7.41%.

#### 4-Hydroxy-3-methyl-4-phenyl-2-thioxothiazolidin-5-yl)indolin-2-one (3h)

White solid; yield (93 mg, 87%); m.p. 164–166 °C; ^1^H NMR (500 MHz, CDCl_3_) δ 3.09 (3H, s), 4.44 (1H, d, *J* = 5.0 Hz), 4.46 (1H, d, *J* = 5.0 Hz), 6.98 (1H, d, *J* = 10.0 Hz), 7.07 (2H, t, *J* = 5.0 Hz), 7.32–7.59 (6H, m), 7.88 (1H, s), 8.10 (1H, s) ppm; ^13^C NMR (125 MHz, CDCl_3_) δ 32.8, 47.3, 56.7, 100.2, 109.6, 123.8, 124.0, 124.7, 125.9, 129.3, 129.4, 129.5, 141.2, 141.7, 178.4, 191.7 ppm; IR (KBr) ν = 3350, 3300–3150, 1673, 1610 cm^−1^; Anal. Calcd. for C_18_H_16_N_2_O_2_S_2_: C, 60.65; H, 4.52; N, 7.86; Found: C, 60.53; H, 4.60; N, 7.75%.

#### 3-(4-Hydroxy-3-methyl-4-phenyl-2-thioxothiazolidin-5-yl)-1-isopropylindolin-2-one (3i)

White solid; yield (110 mg, 92%); m.p. 180–182 °C; ^1^H NMR (500 MHz, CDCl_3_) δ 1.56 (6H, d, *J* = 5.0 Hz), 3.09 (3H, s), 4.36 (1H, d, *J* = 5.0 Hz), 4.38 (1H, d, *J* = 5.0 Hz), 4.63 (1H, septet, *J* = 5.0 Hz), 7.06 (2H, t, *J* = 5.0 Hz), 7.10 (1H, d, , *J* = 10.0 Hz), 7.32–7.58 (6H, m), 8.32 (1H, s) ppm; ^13^C NMR (125 MHz, CDCl_3_) δ 19.3, 19.6, 32.7, 45.4, 46.7, 57.4, 100.2, 110.8, 123.3, 123.9, 124.8, 125.9, 129.3, 129.3, 141.8, 143.2, 176.5, 191.5 ppm; Anal. Calcd. for C_21_H_22_N_2_O_2_S_2_: C, 63.29; H, 5.56; N, 7.03; Found: C, 63.13; H, 5.64; N, 6.85%.

#### Ethyl-2-(3-(4-hydroxy-3-methyl-4-phenyl-2-thioxothiazolidin-5-yl)-2-oxoindolin-1-yl)acetate (3j)

White solid; yield (126 mg, 95%); m.p. 142–144 °C; ^1^H NMR (500 MHz, CDCl_3_) δ 1.31 (3H, t, *J* = 5.0 Hz), 3.08 (3H, s), 4.28 (2H, q, *J* = 5.0 Hz), 4.42 (1H, d, *J* = 15.0 Hz), 4.47 (1H, d, *J* = 5.0 Hz), 4.53 (1H, d, *J* = 5.0 Hz), 4.68 (1H, d, *J* = 15.0 Hz), 6.83 (1H, d, *J* = 10.0 Hz), 7.12 (2H, t, *J* = 5.0 Hz), 7.30–7.61 (6H, m), 7.88 (1H, s) ppm; ^13^C NMR (125 MHz, CDCl_3_) δ 14.3, 32.8, 42.1, 46.8, 56.6, 62.3, 100.2, 109.3, 123.9, 124.2, 124.8, 125.1, 129.3, 129.3, 129.5, 141.8, 143.0, 166.8, 176.9, 191.6 ppm; Anal. Calcd. for C_22_H_22_N_2_O_4_S_2_: C, 59.71; H, 5.01; N, 6.33; Found: C, 59.63; H, 5.11; N, 6.21%.

#### 3-(4-Hydroxy-3-methyl-4-phenyl-2-thioxothiazolidin-5-yl)-1-(naphthalen-1-ylmethyl) indolin-2-one (3k)

White solid; yield (119 mg, 80%); m.p. 176–178 °C; ^1^H NMR (500 MHz, CDCl_3_) δ 3.11 (3H, s), 4.52 (1H, d, *J* = 5.0 Hz), 4.61 (1H, d, *J* = 5.0 Hz), 5.44 (1H, d, *J* = 10.0 Hz), 5.57 (1H, d, *J* = 10.0 Hz), 6.74(1H, d, *J* = 10.0 Hz), 7.05–7.21 (3H, m), 7.44–7.69 (9H, m), 7.84 (1H, d, *J* = 10.0 Hz), 7.94 (1H, d, *J* = 10.0 Hz), 8.11 (1H, d, *J* = 10.0 Hz) 8.22 (1H, s) ppm; ^13^C NMR (125 MHz, CDCl_3_) δ 32.8, 42.7, 47.2, 56.9, 100.3, 110.6, 122.6, 123.7, 124.0, 124.7, 124.8, 125.5, 126.0, 126.1, 126.9, 128.6, 129.1, 129.2, 129.3, 129.4, 129.5, 130.9, 133.9, 141.8, 143.8, 177.1, 191.3 ppm; Anal. Calcd. for C_29_H_24_N_2_O_2_S_2_: C, 70.13; H, 4.87; N, 5.64; Found: C, 69.97; H, 5.04; N, 5.49%.

#### 1-Benzyl-3-(3-ethyl-4-hydroxy-4-phenyl-2-thioxothiazolidin-5-yl) indolin-2-one (3l)

White solid; yield (114 mg, 83%); m.p. 152–154 °C; ^1^H NMR (500 MHz, CDCl_3_) δ 1.29 (3H, t, *J* = 5.0 Hz), 3.35 (1H, q, *J* = 10.0 Hz), 3.94 (1H, q, *J* = 5.0 Hz), 4.42 (1H, d, *J* = 5.0 Hz), 4.52 (1H, d, *J* = 5.0 Hz), 4.79 (1H, d, *J* = 15.0 Hz), 5.25 (1H, d, *J* = 15.0 Hz), 6.82 (1H, d, *J* = 10.0 Hz), 7.06 (2H, t, *J* = 5.0 Hz), 7.18–7.65 (11H, m), 8.26 (1H, s) ppm; ^13^C NMR (125 MHz, CDCl_3_) δ 13.4, 42.3, 44.7, 47.1, 57.0, 100.9, 110.3, 123.7, 123.8, 124.8, 125.5, 127.6, 128.1, 129.1, 129.2, 129.3, 129.4, 134.5, 142.6, 143.6, 177.0, 190.8 ppm; Anal. Calcd. for C_26_H_24_N_2_O_2_S_2_: C, 67.80; H, 5.25; N, 6.08; Found: C, 67.61; H, 5.37; N, 5.93%.

#### 1-Allyl-3-(3-ethyl-4-hydroxy-4-phenyl-2-thioxothiazolidin-5-yl) indolin-2-one (3m)

White solid; yield (93 mg, 76%); m.p. 136–138 °C; ^1^H NMR (500 MHz, CDCl_3_) δ 1.26 (3H, t,* J* = 10.0 Hz), 3.32 (1H, q, *J* = 10.0 Hz), 3.90 (1H, q, *J* = 10.0 Hz), 4.37 (1H, d.d, *J* = 5.0 Hz), 4.41 (1H, d, *J* = 5.0 Hz), 4.46 (1H, d.d, *J* = 5.0 Hz), 4.51 (1H, d, *J* = 5.0 Hz), 5.33 (2H, quintet, *J* = 15.0 Hz), 5.85 (1H, m), 6.92 (1H, d, *J* = 10.0 Hz), 7.09 (2H, t, *J* = 5.0 Hz), 7.34–7.62 (6H, m), 8.20 (1H, s) ppm; ^13^C NMR (125 MHz, CDCl_3_) δ 13.4, 42.3, 43.1, 47.0, 56.9, 100.9, 110.1, 118.5, 123.7, 123.8, 124.8, 125.4, 129.1, 129.3, 129.4, 130.2, 142.6, 157.5, 176.5, 190.8 ppm; Anal. Calcd. for C_22_H_22_N_2_O_2_S_2_: C, 64.36; H, 5.40; N, 6.82; Found: C, 64.27; H, 5.51; N, 6.68%.

#### 3-(3-Ethyl-4-hydroxy-4-phenyl-2-thioxothiazolidin-5-yl)-1-propylindolin-2-one (3n)

White solid; yield (91 mg, 74%); m.p. 140–142 °C; ^1^H NMR (500 MHz, CDCl_3_) δ 1.04 (3H, t,* J* = 10.0 Hz), 1.26 (3H, t,* J* = 10.0 Hz), 1.79 (2H, m), 3.32 (1H, m), 3.72 (1H, m), 3.89 (2H, m), 4.39 (1H, d,* J* = 5.0 Hz), 4.41 (1H, d, *J* = 5.0 Hz), 6.95 (1H, d, *J* = 10.0 Hz), 7.07 (2H, t, *J* = 5.0 Hz), 7.26–7.62 (6H, m), 8.34 (1H, s) ppm; ^13^C NMR (125 MHz, CDCl_3_) δ 11.6, 13.4, 20.8, 40.2, 42.6, 47.0, 56.9, 100.9, 109.6, 123.6, 123.8, 124.8, 125.6, 129.1, 129.2, 129.4, 142.7, 144.0, 176.7, 190.9 ppm; Anal. Calcd. for C_22_H_24_N_2_O_2_S_2_: C, 64.15; H, 5.86; N, 6.79; Found: C, 63.81; H, 5.92; N, 6.58%.

#### 5-Chloro-3-(3-ethyl-4-hydroxy-4-phenyl-2-thioxothiazolidin-5-yl)-1-propylindolin-2-one (3o)

White solid; yield (114 mg, 85%); m.p. 134–136 °C; ^1^H NMR (500 MHz, CDCl_3_) δ 1.03 (3H, t,* J* = 10.0 Hz), 1.25 (3H, t,* J* = 10.0 Hz), 1.78 (2H, m), 3.30 (1H, t,* J* = 5.0 Hz), 3.68 (1H, t,* J* = 5.0 Hz), 3.88 (2H, m), 4.32 (1H, d,* J* = 5.0 Hz), 4.42 (1H, d, *J* = 5.0 Hz), 6.88 (1H, d, *J* = 10.0 Hz), 7.07 (1H, s), 7.32–7.60 (6H, m), 8.12 (1H, s) ppm; ^13^C NMR (125 MHz, CDCl_3_) δ 11.5, 13.3, 20.8, 42.3, 42.7, 47.0, 56.7, 100.9, 110.5, 121.4, 124.3, 124.7, 129.2, 129.4, 142.4, 156.2, 176.3, 190.5 ppm; Anal. Calcd. for C_22_H_23_ClN_2_O_2_S_2_: C, 59.11; H, 5.19; N, 6.27; Found: C, 58.93; H, 5.24; N, 6.12%.

#### Ethyl-2-(3-(3-ethyl-4-hydroxy-4-phenyl-2-thioxothiazolidin-5-yl)-2-oxoindolin-1-yl)acetate (3p)

White solid; yield (103 mg, 75%); m.p. 138–140 °C; ^1^H NMR (500 MHz, CDCl_3_) δ 1.26 (3H, t, *J* = 5.0 Hz), 1.32 (3H, t, *J* = 5.0 Hz), 3.30 (1H, q, *J* = 5.0 Hz), 3.91 (1H, q, *J* = 5.0 Hz), 4.28 (2H, q, *J* = 5.0 Hz), 4.40 (1H, d, *J* = 15.0 Hz), 4.44 (1H, d, *J* = 5.0 Hz), 4.53 (1H, d, *J* = 5.0 Hz), 4.73 (1H, d, *J* = 15.0 Hz), 6.83 (1H, d, *J* = 10.0 Hz), 7.10 (2H, t, *J* = 5.0 Hz), 7.33–7.61 (6H, m), 7.91 (1H, s) ppm; ^13^C NMR (125 MHz, CDCl_3_) δ 13.4, 14.3, 42.1, 42.3, 47.0, 56.6, 62.3, 100.9, 109.3, 123.9, 124.1, 124.8, 125.1, 129.2, 129.3, 129.5, 142.6, 143.1, 166.9, 176.9, 191.0 ppm; Anal. Calcd. for C_23_H_24_N_2_O_4_S_2_: C, 60.51; H, 5.30; N, 6.14; Found: C, 60.38; H, 5.42; N, 5.97%.

#### 1-Benzyl-5-chloro-3-(3-ethyl-4-hydroxy-4-phenyl-2-thioxothiazolidin-5-yl) indolin-2-one (3q)

White solid; yield (121 mg, 82%); m.p. 198–200 °C; ^1^H NMR (500 MHz, CDCl_3_) δ 1.57 (3H, t, *J* = 5.0 Hz), 3.53 (2H, q, *J* = 10.0 Hz), 3.94 (1H, d, *J* = 5.0 Hz), 4.16 (1H, d, *J* = 5.0 Hz), 4.99 (2H, s), 6.66 (1H, d, *J* = 10.0 Hz), 7.14 (1H, d, *J* = 5.0 Hz), 7.27–7.63 (10H, m), 8.02 (1H,s) 8.04 (1H, s) ppm; ^13^C NMR (125 MHz, CDCl_3_) δ 39.9, 41.3, 44.2, 110.0, 125.0, 127.3, 127.8, 127.9, 128.0, 128.3, 128.3, 128.8, 129.0, 130.8, 133.7, 135.5, 142.1, 177.4, 196.5 ppm; Anal. Calcd. for C_26_H_23_ClN_2_O_2_S_2_: C, 63.08; H, 4.68; N, 5.66; Found: C, 62.81; H, 4.76; N, 5.41%.

#### 3-(3-Ethyl-4-hydroxy-4-phenyl-2-thioxothiazolidin-5-yl)-1-methylindolin-2-one (3r)

White solid; yield (106 mg, 92%); m.p. 140–142 °C; ^1^H NMR (500 MHz, CDCl_3_) δ 1.27 (3H, t, *J* = 5.0 Hz), 3.30 (1H, m), 3.34 (3H, s), 3.90 (1H, m), 4.42 (1H, d, *J* = 5.0 Hz), 4.43 (1H, d, *J* = 5.0 Hz), 6.94 (1H, d, *J* = 10.0 Hz), 7.09 (2H, m), 7.38–7.62 (6H, m), 8.30 (1H, s) ppm; ^13^C NMR (125 MHz, CDCl_3_) δ 13.4, 27.0, 42.3, 47.1, 56.7, 101.0, 109.3, 123.6, 123.8, 124.8, 125.3, 129.1, 129.3, 129.5, 142.8, 144.3, 176.7, 190.9 ppm; Anal. Calcd. for C_20_H_20_N_2_O_2_S_2_: C, 62.47; H, 5.24; N, 7.29; Found: C, 62.21; H, 5.33; N, 7.10%.

#### 3-(-3-Benzyl-4-hydroxy-4-phenyl-2-thioxothiazolidin-5-yl)-1-propylindolin-2-one (3s)

White solid; yield (125 mg, 88%); m.p. 130–132 °C; ^1^H NMR (500 MHz, CDCl_3_) δ 1.07 (3H, t,* J* = 10.0 Hz), 1.81 (2H, m), 3.70 (1H, t,* J* = 10.0 Hz), 3.87(1H, t,* J* = 10.0 Hz), 4.36 (1H, d, *J* = 5.0 Hz), 4.48 (1H, d, *J* = 5.0 Hz), 4.52 (1H, d, *J* = 10.0 Hz), 5.12 (1H, d, *J* = 10.0 Hz), 6.95 (1H, d, *J* = 10.0 Hz), 7.07 (2H, t, *J* = 5.0 Hz), 7.20–7.59 (11H, m), 8.25 (1H, s) ppm; ^13^C NMR (125 MHz, CDCl_3_) δ 11.7, 20.9, 42.6, 46.7, 50.2, 57.0, 101.0, 109.6, 123.6, 123.8, 125.0, 125.5, 127.0, 128.0, 128.4, 129.0, 129.2, 129.5, 136.7, 142.6, 143.9, 176.5, 193.0 ppm; Anal. Calcd. for C_27_H_26_N_2_O_2_S_2_: C, 68.33; H, 5.52; N, 5.90; Found: C, 68.10; H, 5.61; N, 5.76%.

#### 3-(3-Benzyl-4-hydroxy-4-phenyl-2-thioxothiazolidin-5-yl)-1-methylindolin-2-one (3t)

White solid; yield (122 mg, 91%); m.p. 140–142 °C; ^1^H NMR (500 MHz, CDCl_3_) δ 3.34 (3H, s), 4.38 (1H, d, *J* = 5.0 Hz), 4.48 (1H, d, *J* = 10.0 Hz), 4.51 (1H, d, *J* = 5.0 Hz), 5.14 (1H, d, *J* = 10.0 Hz), 6.93 (1H, d, *J* = 10.0 Hz), 7.09 (2H, t, *J* = 5.0 Hz), 7.21–7.59 (11H, m), 8.17 (1H, s) ppm; ^13^C NMR (125 MHz, CDCl_3_) δ 27.1, 46.7, 50.3, 56.8, 101.1, 109.3, 123.7, 123.8, 125.0, 127.0, 128.0, 128.3, 129.0, 129.2, 129.6, 132.0, 136.6, 142.7, 144.3, 176.5, 193.1 ppm; Anal. Calcd. for C_25_H_22_N_2_O_2_S_2_: C, 67.24; H, 4.97; N, 6.27; Found: C, 66.98; H, 5.11; N, 6.03%.

#### 1-Allyl-3-(3-benzyl-4-hydroxy-4-phenyl-2-thioxothiazolidin-5-yl) indolin-2-one (3u)

White solid; yield (123 mg, 87%); m.p. 132–134°C; ^1^H NMR (500 MHz, CDCl_3_) δ 4.41 (2H, d, *J* = 5.0 Hz), 4.48 (1H, d.d, *J* = 5.0 Hz), 4.50 (1H, d, *J* = 5.0 Hz), 4.52 (1H, d.d, *J* = 5.0 Hz), 5.14 (1H, d, *J* = 10.0 Hz), 5.35 (2H, quintet, *J* = 10.0 Hz), 5.90 (1H, m), 6.93 (1H, d, *J* = 5.0 Hz), 7.08 (2H, t, *J* = 5.0 Hz), 7.20 -7.60 (11H, m), 8.09 (1H, s) ppm; ^13^C NMR (125 MHz, CDCl_3_) δ 43.1, 46.7, 50.2, 57.0, 101.0, 110.2, 118.6, 123.7, 125.0, 125.3, 127.0, 128.0, 128.4, 129.0, 129.2, 129.4, 130.4, 136.6, 142.5, 143.5, 176.3, 193.0 ppm; Anal. Calcd. for C_27_H_24_N_2_O_2_S_2_: C, 68.62; H, 5.12; N, 5.93; Found: C, 68.43; H, 5.26; N, 5.78%.

#### 1-Benzyl-3-(3-benzyl-4-hydroxy-4-phenyl-2-thioxothiazolidin-5-yl) indolin-2-one (3v)

White solid; yield (124 mg, 79%); m.p. 154–156 °C; ^1^H NMR (500 MHz, CDCl_3_) δ 4.46 (1H, d, *J* = 5.0 Hz), 4.49 (1H, d, *J* = 5.0 Hz), 4.51 (1H, d, *J* = 5.0 Hz), 4.80 (1H, d, *J* = 10.0 Hz), 5.20 (2H, d, *J* = 10.0 Hz), 6.82 (1H, d, *J* = 10.0 Hz), 7.05 (2H, t, *J* = 5.0 Hz), 7.19 -7.62 (16H, m), 8.10 (1H, s) ppm; ^13^C NMR (125 MHz, CDCl_3_) δ 44.7, 46.8, 50.3, 57.0, 101.1, 110.2, 123.7, 123.8, 124.9, 125.4, 127.0, 127.6, 128.0, 128.1, 128.5, 129.0, 129.2, 129.2, 129.4, 134.7, 136.7, 142.6, 143.5, 176.7, 193.0 ppm; Anal. Calcd. for C_31_H_26_N_2_O_2_S_2_: C, 71.24; H, 5.01; N, 5.36; Found: C, 71.02; H, 5.12; N, 5.10%.

#### 3-(3-Benzyl-4-hydroxy-4-phenyl-2-thioxothiazolidin-5-yl)-1-(naphthalen-1-ylmethyl) indolin-2-one (3w)

White solid; yield (132 mg, 77%); m.p. 140–142 °C; ^1^H NMR (500 MHz, CDCl_3_) δ 4.49 (1H, d, *J* = 5.0 Hz), 4.52 (1H, d, *J* = 5.0 Hz), 4.56 (1H, d, *J* = 10.0 Hz), 5.19 (1H, d, *J* = 10.0 Hz), 5.43 (1H, d, *J* = 10.0 Hz), 5.58 (1H, d, *J* = 10.0 Hz), 6.75(1H, d, *J* = 10.0 Hz), 7.05–7.69 (17H, m), 7.86 (1H, d, *J* = 10.0 Hz), 7.95 (1H, d, *J* = 10.0 Hz), 8.14 (1H, d, *J* = 10.0 Hz) 8.16 (1H, s) ppm; ^13^C NMR (125 MHz, CDCl_3_) δ 42.8, 47.0, 50.3, 56.9, 100.0, 101.1, 110.6, 122.7, 123.7, 123.9, 124.9, 125.0, 125.9, 126.2, 127.0, 127.0, 128.0, 128.3, 128.7, 129.1, 129.2, 129.3, 129.3, 129.5, 130.9, 133.9, 136.6, 142.6, 143.8, 176.9, 192.9 pm; Anal. Calcd. for C_35_H_28_N_2_O_2_S_2_: C, 73.40; H, 4.93; N, 4.89; Found: C, 73.27; H, 5.09; N, 4.71%.

#### 4-Hydroxy-4-phenyl-3-(1-phenylethyl)-2-thioxothiazolidin-5-yl)-1-methylindolin-2-one (3x)

White solid; yield (78 mg, 57%); ^1^H NMR (500 MHz, CDCl_3_) δ 3.02 (3H, s), 3.33 (3H, d, *J* = 5.0 Hz ), 3.91 (1H, q, *J* = 5.0 Hz), 5.94 (1H, m), 6.25 (1H, d, *J* = 5.0 Hz), 6.52 (1H, d, *J* = 5.0 Hz), 6.85 (1H, d, *J* = 10.0 Hz), 7.01 (1H, t, *J* = 5.0 Hz), 7.14 (2H, m), 7.37 (2H, t, *J* = 10.0 Hz), 7.46 (4H, m), 7.67 (1H, d, *J* = 5.0 Hz), 7.80 (1H, d, *J* = 10.0 Hz), 7.87 (1H, d, *J* = 5.0 Hz) 7.90 (1H, s) ppm; ^13^C NMR (125 MHz, CDCl_3_) δ 26.0, 26.8, 46.1, 52.8, 56.9, 65.8, 86.5, 107.3, 108.7, 121.6, 123.7, 126.2, 127.7, 128.5, 128.7, 133.6, 135.4, 137.9, 143.2, 143.9, 177.3, 193.7.

#### 1-(1-Allyl-2-oxoindolin-3-yl)-2-oxo-2-phenylethyldimethyl-carbamodithioate (4a)

White solid; yield (102 mg, 83%); m.p. 114–116 °C; ^1^H NMR (500 MHz, CDCl_3_) δ 3.38 (3H, s), 3.62 (3H, s), 3.92 (1H, d, *J* = 5.0 Hz), 4.47 (2H, d.d, *J* = 5.0 Hz), 5.35 (2H, d.d, *J* = 5.0 Hz), 5.97 (1H, quintet, *J* = 5.0 Hz), 6.84 (2H, d, *J* = 5.0 Hz), 7.01 (1H, d, *J* = 5.0 Hz), 7.25 (1H, d, *J* = 5.0 Hz), 7.43–7.58 (4H, m), 8.02 (2H, d ,* J* = 10.0 Hz) ppm; ^13^C NMR (125 MHz, CDCl_3_) δ 35.2, 42.5, 46.2, 57.5, 109.2, 117.8, 122.9, 126.6, 127.8, 128.5, 128.7, 128.9, 131.2, 131.7, 132.6, 133.9, 136.4, 137.7, 176.1, 191.3, 194.1 ppm; Anal. Calcd. for C_22_H_22_N_2_O_2_S_2_: C, 64.36; H, 5.40; N, 6.82; Found: C, 64.24; H, 5.48; N, 6.67%.

#### 1-(1-Benzyl-2-oxoindolin-3-yl)-2-oxo-2-phenylethyldimethyl-carbamodithioate (4b)

White solid; yield (102 mg, 74%); m.p. 140–142 °C; ^1^H NMR (500 MHz, CDCl_3_) δ 3.38 (3H, s), 3.63 (3H, s), 4.01 (1H, d, *J* = 5.0 Hz), 5.05 (2H, d, *J* = 10.0 Hz), 6.71 (1H, d, *J* = 5.0 Hz), 6.88 (1H, d, *J* = 5.0 Hz), 6.98 (1H, t, *J* = 5.0 Hz), 7.16 (1H, t, *J* = 5.0 Hz), 7.34–7.59 (9H, m), 8.05 (2H, d,* J* = 10.0 Hz) ppm; ^13^C NMR (125 MHz, CDCl_3_) δ 41.7, 44.1, 46.3, 46.5, 57.5, 94.4, 109.1, 122.4, 123.9, 126.5, 127.5, 127.6, 128.5, 128.7, 129.0, 133.6, 134.9, 136.2, 143.6, 176.3, 194.2, 194.2 ppm; Anal. Calcd. for C_26_H_24_N_2_O_2_S_2_: C, 67.80; H, 5.25; N, 6.08; Found: C, 67.57; H, 5.37; N, 5.92%.

#### 1-(1-Allyl-5-chloro-2-oxoindolin-3-yl)-2-oxo-2-phenylethyl dimethylcarbamodithioate (4c)

White solid; yield (108 mg, 81%); m.p. 110–112 °C; ^1^H NMR (500 MHz, CDCl_3_) δ 3.39 (3H, s), 3.64 (3H, s), 3.90 (1H, d, *J* = 5.0 Hz), 4.45 (2H, d.d, *J* = 5.0 Hz), 5.34 (2H, d.d, *J* = 5.0 Hz), 5.95 (1H, quintet, *J* = 5.0 Hz), 6.79 (2H, d, *J* = 5.0 Hz), 7.20 (1H,d, *J* = 5.0 Hz), 7.45–7.60 (4H, m), 7.97 (2H, d,* J* = 10.0 Hz) ppm; ^13^C NMR (125 MHz, CDCl_3_) δ 41.6, 42.6, 46.2, 46.6, 57.5, 100.0, 109.9, 117.8, 124.2, 127.9, 128.4, 128.9, 129.3, 131.3, 132.2, 133.8, 134.7, 164.6, 184.5, 193.9 ppm; Anal. Calcd. for C_22_H_21_ClN_2_O_2_S_2_: C, 59.38; H, 4.76; N, 6.30; Found: C, 59.22; H, 4.84; N, 6.21%.

#### 1-(1-Benzyl-5-chloro-2-oxoindolin-3-yl)-2-oxo-2-phenylethyl dimethylcarbamodithioate (4d)

White solid; yield (119 mg, 80%); m.p. 132–134 °C; ^1^H NMR (500 MHz, CDCl_3_) δ 3.39 (3H, s), 3.99 (3H, s), 3.99 (1H, d, *J* = 5.0 Hz), 5.03 (2H, d, *J* = 10.0 Hz), 6.61 (1H, d, *J* = 5.0 Hz), 6.85 (1H, d, *J* = 5.0 Hz), 7.12(1H, d, *J* = 5.0 Hz), 7.35–7.60 (9H, m), 8.01 (2H, d,* J* = 10.0 Hz) ppm; ^13^C NMR (125 MHz, CDCl_3_) δ 41.7, 44.2, 46.4, 46.7, 57.5, 110.0, 124.3, 127.5, 127.7, 128.4, 128.8, 128.8, 129.0, 133.8, 134.6, 135.8, 142.2, 176.0, 193.9, 194.0 ppm; Anal. Calcd. for C_26_H_23_ClN_2_O_2_S_2_: C, 63.08; H, 4.68; N, 5.66; Found: C, 62.81; H, 4.81; N, 5.49%.

#### 1,1ʹʹ-Diallyl-2ʹ-benzoyl-4ʹ-hydroxy-5ʹ-methoxy-4ʹ-phenyldispiro[indoline-3,1ʹ-cyclopentane-3ʹ,3ʹʹ-indoline]-2,2ʹʹ-*dione* (5a)

White solid; yield (79 mg, 86%); m.p. 258–260 °C; ^1^H NMR (500 MHz, DMSO-d_6_) δ 2.95 (3H, s), 3.93 (2H, d.d, *J* = 15.0 Hz), 4.21 (1H, d, *J* = 15.0 Hz), 4.53 (2H, d.d, *J* = 15.0 Hz), 4.80 (1H, d, *J* = 15.0 Hz), 5.14 (1H, quintet, *J* = 10.0 Hz), 5.21 (2H, t, *J* = 15.0 Hz), 5.27 (1H, *s*), 5.77 (1H, quintet, *J* = 10.0 Hz), 5.96 (1H, *s*), 6.57 (1H, d, *J* = 5.0 Hz), 6.63 (1H, d, *J* = 5.0 Hz), 6.68 (1H, s), 6.98–7.23 (13H, m), 7.374 (1H, t, *J* = 5.0 Hz), 7.86 (1H, d, *J* = 5.0 Hz), 8.06 (1H, d, *J* = 5.0 Hz) ppm; ^13^C NMR (125 MHz, DMSO-d_6_) δ 41.9, 43.1, 58.3, 60.0, 60.7, 61.6, 84.5, 89.3, 100.0, 108.8, 109.4, 116.9, 118.4, 122.0, 123.9, 125.0, 125.9, 126.2, 126.6, 127.3, 127.6, 127.9, 129.1, 130.6, 131.6, 131.9, 133.3, 136.8, 137.6, 142.5, 143.8, 176.3, 179.3, 196.6 ppm; Anal. Calcd. for C_39_H_34_N_2_O_5_: C, 76.70; H, 5.61; N, 4.59; Found: C, 76.51; H, 5.76; N, 4.34%.

#### 2ʹ-Benzoyl-4ʹ-hydroxy-1,1ʹʹ-dimethyl-5ʹ-(octyloxy)-4ʹ-phenyldispiro[indoline-3,1ʹ-cyclopentane-3ʹ,3ʹʹ-indoline]-2,2ʹʹ-*dione* (5b)

White solid; yield (88 mg, 89%); m.p. 222–224 °C; ^1^H NMR (500 MHz, DMSO-d6) δ 0.79–1.19 (15H, m), 2.79 (3H,s), 2.85 (1H, t, *J* = 10.0 Hz), 3.08 (3H,s), 3.17 (1H, t, *J* = 10.0 Hz), 5.22 (1H, s), 5.99 (1H, s), 6.56 (1H, d, *J* = 5.0 Hz), 6.75 (2H, t, *J* = 5.0 Hz), 6.97–7.49 (14H, m), 7.82 (1H, d, *J* = 5.0 Hz), 8.09 (1H, d, *J* = 5.0 Hz) ppm; ^13^C NMR (125 MHz, DMSO-d6) δ 14.4, 22.5, 25.8, 26.2, 27.1, 28.9, 29.0, 29.6, 31.6, 58.5, 61.6, 64.6, 84.6, 87.5, 108.0, 108.8, 112.7, 122.0, 123.8, 125.7, 126.1, 126.4, 126.7, 127.2, 127.3, 127.8, 128.4, 129.1, 130.6, 131.6, 133.2, 136.8, 137.5, 143.3, 144.5, 176.6, 179.7, 196.6 ppm; Anal. Calcd. for C_42_H_44_N_2_O_5_: C, 76.80; H, 6.75; N, 4.27; Found: C, 76.64; H, 6.83; N, 4.08%.

#### 1,1ʹʹ-Diallyl-2ʹ-benzoyl-4ʹ-hydroxy-4ʹ-phenyl-5ʹ-propoxydispiro[indoline-3,1ʹ-cyclopentane-3ʹ,3ʹʹ-indoline]-2,2ʹʹ-*dione* (5c)

White solid; yield (76 mg, 79%); m.p. 166–168 °C; ^1^H NMR (500 MHz, DMSO-d_6_) δ 0.49 (3H, t, *J* = 10.0 Hz), 1.10 (2H, m), 2.88 (1H, q, *J* = 10.0 Hz), 3.13 (1H, q, *J* = 10.0 Hz), 3.97 (2H, d.d,* J* = 15.0 Hz), 4.21 (1H, d, *J* = 15.0 Hz), 4.51 (2H, d.d, *J* = 15.0 Hz), 5.81 (1H, d, *J* = 15.0 Hz), 5.16 (1H, quintet, *J* = 10.0 Hz), 5.22 (1H, t, *J* = 15.0 Hz), 5.28 (1H, t, *J* = 10.0 Hz), 5.75 (1H, quintet, *J* = 10.0 Hz), 6.04 (1H, *s*), 6.59 (1H, d, *J* = 5.0 Hz), 6.64 (1H, d, *J* = 5.0 Hz), 6.71 (1H, s), 6.98–7.24 (13H, m), 7.37 (1H, t, *J* = 5.0 Hz), 7.86 (1H, d, *J* = 5.0 Hz), 8.04 (1H, d, *J* = 5.0 Hz) ppm; ^13^C NMR (125 MHz, DMSO-d_6_) δ 10.7, 22.9, 41.9, 43.2, 58.4, 61.6, 64.5, 73.8, 84.5, 87.9, 100.0, 101.0, 108.8, 109.3, 116.9, 119.0, 122.0, 123.8, 125.9, 126.2, 126.7, 127.3, 127.5, 127.8, 128.7, 129.1, 130.5, 131.5, 131.9, 133.2, 136.8, 137.6, 142.6, 143.7, 176.3, 179.4, 196.6 ppm; Anal. Calcd. for C_41_H_38_N_2_O_5_: C, 77.09; H, 6.00; N, 4.39; Found: C, 76.89; H, 6.14; N, 4.21%.

#### 4ʹ-Hydroxy-2ʹ-methyl-4ʹ-phenyl-5ʹ-propoxy-1,1ʹʹ-dipropyldispiro[indoline-3,1ʹ-cyclopentane-3ʹ,3ʹʹ-indoline]-2,2ʹʹ-*dione* (5d)

White solid; yield (72 mg, 75%); m.p. 210–212 °C; ^1^H NMR (500 MHz, DMSO-d_6_) δ 0.49 (3H, t, *J* = 10.0 Hz), 0.55 (3H, t, *J* = 10.0 Hz), 0.93 (3H, t, *J* = 10.0 Hz), 1.00 (2H, m), 1.11 (2H, m), 1.47–1.66 (2H, m), 2.86–3.15 (2H, m), 3.20–3.38 (2H, m), 3.50–3.77 (2H, m), 5.23 (1H, s), 6.03 (1H, s), 6.66 (1H, d, *J* = 10.0 Hz), 6.76 (1H, s), 6.79 (2H, d, *J* = 10.0 Hz), 6.96 (2H, d, *J* = 5.0 Hz), 7.02–7.19 (9H, m), 7.25 (1H, t, *J* = 10.0 Hz), 7.36 (1H, t, *J* = 10.0 Hz), 7.84 (1H, d, *J* = 5.0 Hz), 8.04 (1H, d, *J* = 5.0 Hz) ppm; ^13^C NMR (125 MHz, DMSO-d_6_) δ 10.7, 11.6, 11.9, 20.4, 21.0, 23.0, 41.3, 42.5, 58.4, 61.5, 64.2, 73.8, 84.6, 87.9, 100.0, 108.2, 109.0, 121.7, 123.6, 125.9, 126.1, 126.6, 127.2, 127.4, 128.6, 129.1, 130.7, 133.1, 136.9, 137.7, 143.1, 144.4, 176.4, 179.6, 196.6 ppm; Anal. Calcd. for C_41_H_42_N_2_O_5_: C, 76.61; H, 6.59; N, 4.36; Found: C, 76.43; H, 5.71; N, 4.19.

#### 2ʹ-Benzoyl-1,1ʹʹ-di(but-2-en-1-yl)-4ʹ-hydroxy-5ʹ-methoxy-4ʹ-phenyldispiro[indoline-3,1ʹ-cyclopentane-3ʹ,3ʹʹ-indoline]-2,2ʹʹ-*dione* (5e)

White solid; yield (79 mg, 83%); m.p. 206–208 °C; ^1^H NMR (500 MHz, DMSO-d_6_) δ 1.41 (3H, d, *J* = 10.0 Hz), 1.65 (3H, d, *J* = 10.0 Hz), 2.93 (3H, t, *J* = 10.0 Hz), 3.80 (1H, d, *J* = 10.0 Hz), 3.98 (1H, d, *J* = 10.0 Hz), 4.19 (1H, d, *J* = 10.0 Hz), 4.41 (1H, d, *J* = 10.0 Hz), 4.46 (1H, m), 5.93 (1H, m), 5.26 (1H, *s*), 5.38 (1H, m), 5.72 (1H, m), 5.94 (1H, *s*), 6.54–6.67 (3H, m), 6.92–7.24 (13H, m), 7.36 (1H, t, *J* = 5.0 Hz), 7.84 (1H, d, *J* = 5.0 Hz), 8.04 (1H, d, *J* = 5.0 Hz) ppm; ^13^C NMR (125 MHz, DMSO-d_6_) δ 13.4, 18.0, 36.6, 37.7, 41.0, 42.3, 58.2, 59.8, 61.4, 64.2, 84.5, 89.3, 108.7, 109.4, 121.9, 123.8, 124.2, 124.6, 125.9, 126.1, 126.7, 127.3, 127.5, 127.8, 128.6, 129.1, 129.8, 130.7 133.1, 136.8, 137.6, 142.5, 143.9, 176.1, 179.1, 196.5 ppm; Anal. Calcd. for C_41_H_38_N_2_O_5_: C, 77.09; H, 6.00; N, 4.39; Found: C, 76.88; H, 6.08; N, 4.19%.

#### 2ʹ-Benzoyl-1,1ʹʹ-di((E)-but-2-en-1-yl)-5ʹ-ethoxy-4ʹ-hydroxy-4ʹ-phenyldispiro[indoline-3,1ʹ-cyclopentane-3ʹ,3ʹʹ-indoline]-2,2ʹʹ-*dione* (5f)

White solid; yield (79 mg, 81%); m.p. 162–164 °C; ^1^H NMR (500 MHz, DMSO-d_6_) δ 0.72 (3H, t, *J* = 5.0 Hz), 1.41 (3H, d, *J* = 10.0 Hz), 1.64 (3H, d, *J* = 10.0 Hz), 2.95 (1H, q, *J* = 10.0 Hz), 3.23 (1H, q, *J* = 5.0 Hz), 3.80 (1H, d, *J* = 10.0 Hz), 3.95 (1H, d, *J* = 10.0 Hz), 4.19 (1H, d, *J* = 10.0 Hz), 4.42 (1H, d, *J* = 10.0 Hz), 4.4.67 (1H, m), 4.93 (1H, m), 5.26 (1H, *s*), 5.39 (1H, m), 5.73 (1H, m), 6.03 (1H, *s*), 6.56–6.69 (3H, m), 6.94–7.24 (13H, m), 7.36 (1H, t, *J* = 5.0 Hz), 7.84 (1H, d, *J* = 5.0 Hz), 8.02 (1H, d, *J* = 5.0 Hz) ppm; ^13^C NMR (125 MHz, DMSO-d_6_) δ 13.5, 15.6, 18.0, 36.6, 37.6, 41.0, 42.2, 58.4, 61.4, 64.2, 67.6, 84.6, 87.8, 108.7, 109.3, 121.9, 123.7, 124.3, 124.6, 125.9, 126.1, 126.5, 126.7, 127.3, 127.5, 127.8, 128.6, 129.1, 129.8, 130.7 133.1, 136.8, 137.6, 142.6, 143.9, 176.1, 179.2, 196.6 ppm; Anal. Calcd. for C_42_H_40_N_2_O_5_: C, 77.28; H, 6.18; N, 4.29; Found: C, 77.05; H, 6.32; N, 4.10%.

#### 2ʹ-Benzoyl-5ʹ-ethoxy-4ʹ-hydroxy-4ʹ-phenyl-1,1ʹʹ-dimethyldispiro[indoline-3,1ʹ-cyclopentane-3ʹ,3ʹʹ-indoline]-2,2ʹʹ-*dione* (5g)

White solid; yield (70 mg, 85%); m.p. 266–268 °C; ^1^H NMR (500 MHz, DMSO-d_6_) δ 0.69 (3H, t, *J* = 10.0 Hz), 2.79 (3H, s), 2.96 (1H, q, *J* = 10.0 Hz), 3.11 (3H, s), 3.19 (1H, q, J = 10.0 Hz), 5.23 (1H, s), 6.02 (1H, s), 6.58 (1H, d, J = 10.0 Hz), 6.75 (2H, d, J = 10.0 Hz), 6.90–7.13 (10H, m), 7.18 (2H, t, J = 10.0 Hz), 7.26 (1H, t J = 10.0 Hz), 7.35 (1H, t, J = 10.0 Hz), 7.83 (1H, d, J = 5.0 Hz), 8.08 (1H, d, J = 5.0 Hz) ppm; 13C NMR (125 MHz, DMSO-d6) δ 15.7, 26.2, 27.1, 58.6, 61.6, 64.6, 67.4, 84.6, 87.4, 108.0, 108.8, 121.9, 123.8, 125.7, 126.0, 126.4, 126.7, 127.2, 127.3, 127.8, 128.4, 129.1, 130.6, 133.1, 136.8, 137.5, 143.3, 144.6, 176.6, 179.7, 196.6 ppm.

### Supplementary Information

Supplementary Information.

## Data Availability

All data generated or analyzed during this study are included in this article [and its Supplementary Information files]. Crystallographic model data is available through the CCDC under identifier 2327735.
