# Supplementary material for: Regio- and diastereoselective synthesis of thioxothiazolidin-indolin-2-ones, oxoindolin-carbamodithioate hybrids, and their base-catalyzed conversion into dispirocyclopentanebisoxindoles
Source: Sci Rep. 2024 Jun 20;14:14222. doi: 10.1038/s41598-024-65087-0 (PMC11190151; doi:10.1038/s41598-024-65087-0)
Supplement: Supplementary file 1 — Supplementary Information. [file 41598_2024_65087_MOESM1_ESM.pdf]

Regio- and diastereoselective synthesis of thioxothiazolidin-indolin-2-ones, oxoindolin-carbamodithioate hybrids, and their base-catalyzed conversion into dispirocyclopentanebisoxindoles

Bagher Aghamiri, F. Matloubi Moghaddam*, Sara Badpa and Leila Kavoosi

Laboratory of Organic Synthesis and Natural Products, Department of Chemistry, Sharif University of Technology, Azadi Street, PO Box 111559516, Tehran, Iran. Email: matloubi@sharif.edu

Table of Contents

**1. General remarks**	**Page 2**
**2. Experimental Procedure**	**Page 2**
**3. Characterization Data of Products**	**Page 3**
**4. ^{1}H NMR, ^{13}C NMR Spectra of Products**	**Page 20**
**5. D_2O exchange of Product 3d**	**Page 55**
**6. X-ray crystallography analysis data**	**Page 56**
**7. Crystal data and structure refinement for the compound 3c**	**Page 57**

1. General remarks.

All solvents and starting materials were purchased from Merck and Sigma-Aldrich used without any additional purification. Analytical TLC was carried out using Merck 0.2 mm silica gel 60 F-254 Al-plates. ^{1}H NMR and ^{13}C NMR spectra were recorded on a Bruker Avance DRX-500 machine using DMSO-d6 as solvent and TMS as an internal standard at room temperature (DMSO-d_6 ^{1}H-NMR: δ (ppm) =2.50 ppm; ^{13}C-NMR: δ (ppm) =39.9 ppm; $CDCl_3$ ^{1}H-NMR: δ (ppm) = 7.26 and ^{13}C-NMR: δ (ppm) = 77.00 ppm). Chemical shifts were reported in ppm scale. FT-IR spectra of samples were obtained on ABB Bomem MB100 spectrometer with potassium bromide (KBr) pellets. Melting points were determined using an Electrothermal 9100 apparatus and are uncorrected. Elemental analysis was done by LECO Truspec.

2. Experimental procedure for the synthesis of compounds 3, 4, 5.

To a solution of isatin derivatives (**1a-1x**, 0.3mmol) in alcoholic solvents (2.0 ml), phosphonium ylide (**2**, 0.3mmol) was added and stirred at room temperature for 30 min to afford the corresponding isatin chalcone. Upon completion of the reaction, the reaction color changes from orange to red. In the next step, carbon disulfide (0.3 mmol) and primary (1°) amines (0.3mmol) were added to the reaction mixture and stirred for more 1.5 h to form products **3a-3x** (the general procedure used for synthesizing the products **3a-3x** was also employed for compounds **4a-4d** except using secondary (2°) amines in place of primary (1°) amines). Upon completion of the reaction, the reaction color changes from red to pale yellow. In this stage, if we added KOH (15 mg, 0.8 equiv.) to the reaction mixture, products **3** or **4** easily converted to dispirocyclopentanebisoxindoles (**5a-5f**) after 30-90 min of stirring (first, the reaction color changes from pale yellow to red, and then from red to white). After completion of the reaction, the organic products were simply filtered off and the precipitate was washed with EtOH. The pure products were dried in air and directly characterized by ^{1}H NMR, ^{13}C NMR, elemental and FT-IR analysis. In addition, the structure of compound **3c** was confirmed by X-ray crystallographic analysis.

3. Characterization Data of Products

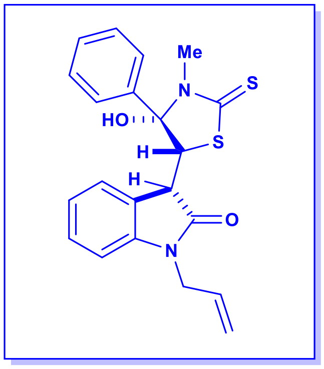

1-allyl-3-(4-hydroxy-3-methyl-4-phenyl-2-thioxothiazolidin-5-yl) indolin-2-one (3a)

white solid; yield (96 mg, 81 %); m.p. 178-180 °C; ^{1}H NMR (500 MHz, $CDCl_3$) δ 3.09 (3H, s), 4.35 (1H, d.d., *J*= 5.0 Hz), 4.44 (1H, d, *J*= 5.0 Hz), 4.46 (1H, d, *J*= 5.0 Hz), 4.53 (1H, d.d, *J*= 5.0 Hz), 5.33 (2H, quintet, *J*= 15.0 Hz), 5.87 (1H, m), 6.93 (1H, d, *J*= 10.0 Hz), 7.08 (2H, t, *J*= 5.0 Hz), 7.31 -7.59 (6H, m), 8.15 (1H, s) ppm; ^{13}C NMR (125 MHz, $CDCl_3$) δ 32.7, 43.2, 46.9, 56.8, 100.2, 110.2, 118.6, 123.7, 123.8, 124.8, 125.4, 129.2, 129.3, 129.4, 130.2, 141.8, 143.5, 176.5, 191.4 ppm; IR (KBr) ν= 3300-3080, 1675, 1640, 1609 cm^{-1}; Anal. Calcd. for $C_{21}H_{20}N_2O_2S_2$: C, 63.61; H, 5.08; N, 7.06; Found: C, 63.45; H, 5.16; N, 6.75 %.

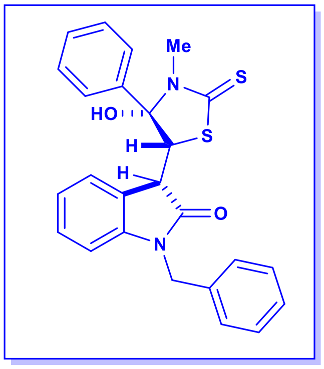

1-benzyl-3-(4-hydroxy-3-methyl-4-phenyl-2-thioxothiazolidin-5-yl) indolin-2-one (3b)

white solid; yield (112 mg, 84 %); m.p. 160-162 °C; ^{1}H NMR (500 MHz, $CDCl_3$) δ 3.12 (3H, s), 4.46 (1H, d, *J*= 5.0 Hz), 4.52 (1H, d, *J*= 5.0 Hz), 4.79 (1H, d, *J*= 10.0 Hz), 5.23 (1H, d, *J*= 10.0 Hz), 6.81 (1H, d, *J*= 10.0 Hz), 7.05 (2H, t, *J*= 5.0 Hz), 7.23 -7.62 (11H, m), 8.19 (1H, s) ppm; ^{13}C NMR (125 MHz, $CDCl_3$) δ 32.7, 44.8, 47.0, 56.9, 100.2, 110.4, 123.7, 123.8, 124.7, 125.4, 127.6, 128.1, 129.1, 129.2, 129.3, 129.4, 134.5, 141.8, 143.5, 176.9, 191.4 ppm; IR (KBr) ν= 3300-3161, 1675, 1614 cm^{-1}; Anal. Calcd. for $C_{25}H_{22}N_2O_2S_2$: C, 67.24; H, 4.97; N, 6.27; Found: C, 67.01; H, 5.11; N, 6.08 %.

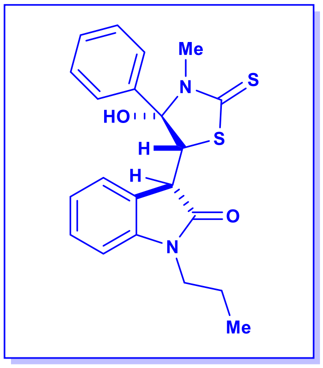

3-(4-hydroxy-3-methyl-4-phenyl-2-thioxothiazolidin-5-yl)-1-propylindolin-2-one (3c)

white solid; yield (102 mg, 86 %); m.p. 172-174 °C; ^{1}H NMR (500 MHz, $CDCl_3$) δ 1.04 (3H, t, *J*= 10.0 Hz), 1.78 (2H, m), 3.09 (3H, s), 3.69 (1H, m), 3.86 (1H, m), 4.42 (1H, d, *J*= 5.0 Hz), 4.43 (1H, d, *J*= 5.0 Hz), 6.96 (1H, d, *J*= 10.0 Hz), 7.08 (2H, t, *J*= 5.0 Hz), 7.34 -7.60 (6H, m), 8.27 (1H, s) ppm; ^{13}C NMR (125 MHz, $CDCl_3$) δ 11.6, 20.8, 32.7, 42.6, 46.8, 56.8, 100.2, 109.6, 123.6, 123.8, 124.7, 124.8, 125.6, 129.2, 129.4, 141.9, 143.9, 176.7, 191.4 ppm; IR (KBr) ν= 3300-3050, 1670, 1609 cm^{-1}; Anal. Calcd. for $C_{21}H_{22}N_2O_2S_2$: C, 63.29; H, 5.56; N, 7.03; Found: C, 62.97; H, 5.68; N, 6.87 %

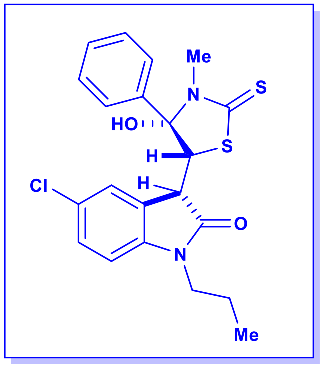

5-chloro-3-(4-hydroxy-3-methyl-4-phenyl-2-thioxothiazolidin-5-yl)-1-propylindolin-2-one (3d)

white solid; yield (100 mg, 77 %); m.p. 150-152 °C; ^{1}H NMR (500 MHz, $CDCl_3$) δ 1.03 (3H, t, *J*= 5.0 Hz), 1.76 (2H, m), 3.08 (3H, s), 3.66 (1H, m), 3.84 (1H, m), 4.37 (1H, d, *J*= 5.0 Hz), 4.42 (1H, d, *J*= 5.0 Hz), 6.88 (1H, d, *J*= 10.0 Hz), 7.08 (1H, s), 7.33 -7.57 (6H, m), 8.06 (1H, s) ppm; ^{13}C NMR (125 MHz, $CDCl_3$) δ 11.5, 20.8, 32.7, 42.8, 46.9, 56.6, 100.1, 100.2, 110.5, 124.3, 124.7, 127.2, 129.3, 129.4, 129.5, 141.6, 142.5, 176.2, 191.1 ppm; IR (KBr) ν = 3300-3056, 1675, 1609 cm^{-1}; Anal. Calcd. for $C_{21}H_{21}ClN_2O_2S_2$: C, 58.25; H, 4.89; N, 6.47; Found: C, 58.01; H, 5.04; N, 6.21 %

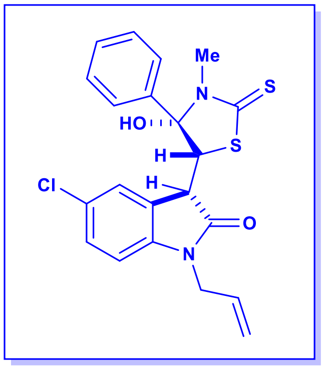

1-allyl-5-chloro-3-(4-hydroxy-3-methyl-4-phenyl-2-thioxothiazolidin-5-yl) indolin-2-one (3e)

white solid; yield (107 mg, 83 %); m.p. 154-156 °C; ^{1}H NMR (500 MHz, $CDCl_3$) δ 3.08 (3H, s), 4.32 (1H, d.d, *J*= 5.0 Hz), 4.39 (1H, d, *J*= 5.0 Hz), 4.47 (1H, d, *J*= 5.0 Hz), 4.54 (1H, d.d, *J*= 5.0 Hz), 5.33 (2H, quintet, *J*= 15.0 Hz), 5.84 (1H, m), 6.86 (1H, d, *J*= 10.0 Hz), 7.09 (1H, s), 7.31 -7.58 (6H, m), 7.95 (1H, s) ppm; ^{13}C NMR (125 MHz, $CDCl_3$) δ 32.8, 43.3, 46.9, 56.6, 100.2, 111.2, 118.9, 124.3, 124.7, 127.1, 129.3, 129.4, 129.9, 141.5, 156.7, 176.5, 191.0 ppm; IR (KBr) ν= 3300-3086, 1672, 1643, 1612 cm^{-1}; Anal. Calcd. for $C_{21}H_{19}ClN_2O_2S_2$: C, 58.53; H, 4.44; N, 6.50; Found: C, 58.22; H, 4.51; N, 6.35 %.

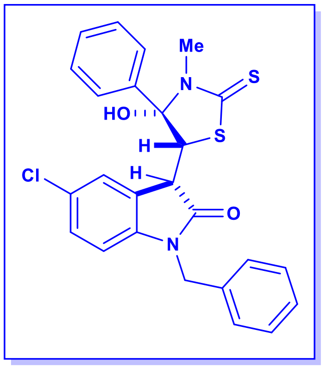

1-benzyl-5-chloro-3-(4-hydroxy-3-methyl-4-phenyl-2-thioxothiazolidin-5-yl) indolin-2-one (3f)

white solid; yield (114 mg, 79 %); m.p. 128-130 °C; ^{1}H NMR (500 MHz, $CDCl_3$) δ 3.12 (3H, s), 4.41 (1H, d, *J*= 5.0 Hz), 4.54 (1H, d, *J*= 5.0 Hz), 4.75 (1H, d, *J*= 10.0 Hz), 5.23 (1H, d, *J*= 10.0 Hz), 6.73 (1H, d, *J*= 10.0 Hz), 7.03 (1H, s), 7.21 -7.60 (11H, m), 7.99 (1H, s) ppm; ^{13}C NMR (125 MHz, $CDCl_3$) δ 32.8, 44.9, 47.1, 56.7, 100.2, 111.3, 124.2, 124.7, 127.1, 127.6, 128.3, 129.2, 129.3, 129.4, 129.5, 129.5, 134.0, 141.5, 142.0, 176.5, 191.0 ppm; IR (KBr) ν= 3350-3100, 1678, 1615 cm^{-1}; Anal. Calcd. for $C_{25}H_{21}ClN_2O_2S_2$: C, 62.42; H, 4.40; N, 5.82; Found: C, 62.15; H, 4.53; N, 5.65%.

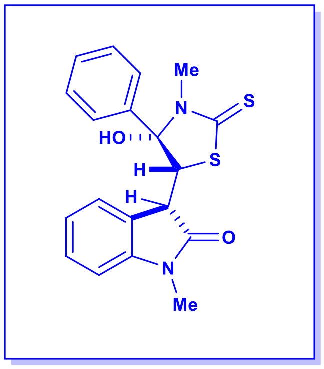

3-(4-hydroxy-3-methyl-4-phenyl-2-thioxothiazolidin-5-yl)-1-methylindolin-2-one (3g)

white solid; yield (101 mg, 91 %); m.p. 170-172 °C; ^{1}H NMR (500 MHz, $CDCl_3$) δ 3.09 (3H, s), 3.33 (3H, s), 4.43 (1H, d, *J*= 5.0 Hz), 4.45 (1H, d, *J*= 5.0 Hz), 6.94 (1H, d, *J*= 10.0 Hz), 7.09 (2H, t, *J*= 5.0 Hz), 7.36 -7.59 (6H, m), 8.26 (1H, s) ppm; ^{13}C NMR (125 MHz, $CDCl_3$) δ 27.0, 32.8, 46.9, 56.7, 100.2, 109.3, 123.6, 123.9, 124.7, 125.3, 129.2, 129.3, 129.5, 142.0, 144.3, 176.6, 191.5 ppm; IR (KBr) ν= 3400-3150, 1676, 1604 cm^{-1}; Anal. Calcd. for $C_{19}H_{18}N_2O_2S_2$: C, 61.60; H, 4.90; N, 7.56; Found: C, 61.35; H, 4.96; N, 7.41 %

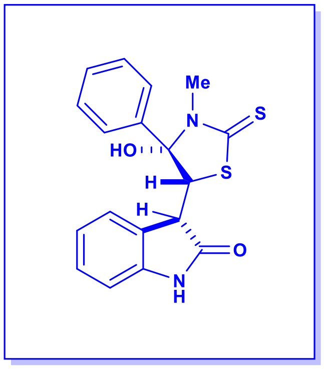

4-hydroxy-3-methyl-4-phenyl-2-thioxothiazolidin-5-yl)indolin-2-one (3h)

white solid; yield (93 mg, 87 %); m.p. 164-166 °C; ^{1}H NMR (500 MHz, $CDCl_3$) δ 3.09 (3H, s), 4.44 (1H, d, *J*= 5.0 Hz), 4.46 (1H, d, *J*= 5.0 Hz), 6.98 (1H, d, *J*= 10.0 Hz), 7.07 (2H, t, *J*= 5.0 Hz), 7.32 -7.59 (6H, m), 7.88 (1H, s), 8.10 (1H, s) ppm; ^{13}C NMR (125 MHz, $CDCl_3$) δ 32.8, 47.3, 56.7, 100.2, 109.6, 123.8, 124.0, 124.7, 125.9, 129.3, 129.4, 129.5, 141.2, 141.7, 178.4, 191.7 ppm; IR (KBr) ν= 3350, 3300-3150, 1673, 1610 cm^{-1}; Anal. Calcd. for $C_{18}H_{16}N_2O_2S_2$: C, 60.65; H, 4.52; N, 7.86; Found: C, 60.53; H, 4.60; N, 7.75 %

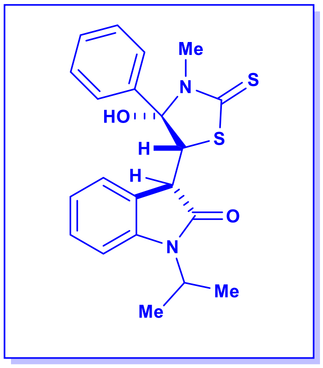

3-(4-hydroxy-3-methyl-4-phenyl-2-thioxothiazolidin-5-yl)-1-isopropylindolin-2-one (3i)

white solid; yield (110 mg, 92%); m.p. 180-182 °C; ^{1}H NMR (500 MHz, $CDCl_3$) δ 1.56 (6H, d, *J*= 5.0 Hz), 3.09 (3H, s), 4.36 (1H, d, *J*= 5.0 Hz), 4.38 (1H, d, *J*= 5.0 Hz), 4.63 (1H, septet, *J*= 5.0 Hz), 7.06 (2H, t, *J*= 5.0 Hz), 7.10 (1H, d, , *J*= 10.0 Hz), 7.32 -7.58 (6H, m), 8.32 (1H, s) ppm; ^{13}C NMR (125 MHz, $CDCl_3$) δ 19.3, 19.6, 32.7, 45.4, 46.7, 57.4, 100.2, 110.8, 123.3, 123.9, 124.8, 125.9, 129.3, 129.3, 141.8, 143.2, 176.5, 191.5 ppm; Anal. Calcd. for $C_{21}H_{22}N_2O_2S_2$: C, 63.29; H, 5.56; N, 7.03; Found: C, 63.13; H, 5.64; N, 6.85 %

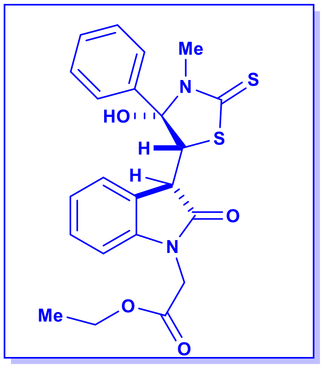

ethyl 2-(3-(4-hydroxy-3-methyl-4-phenyl-2-thioxothiazolidin-5-yl)-2-oxoindolin-1-yl)acetate (3j)

white solid; yield (126 mg, 95%); m.p. 142-144 °C; ^{1}H NMR (500 MHz, $CDCl_3$) δ 1.31 (3H, t, *J*= 5.0 Hz), 3.08 (3H, s), 4.28 (2H, q, *J*= 5.0 Hz), 4.42 (1H, d, *J*= 15.0 Hz), 4.47 (1H, d, *J*= 5.0 Hz), 4.53 (1H, d, *J*= 5.0 Hz), 4.68 (1H, d, *J*= 15.0 Hz), 6.83 (1H, d, *J*= 10.0 Hz), 7.12 (2H, t, *J*= 5.0 Hz), 7.30 -7.61 (6H, m), 7.88 (1H, s) ppm; ^{13}C NMR (125 MHz, $CDCl_3$) δ 14.3, 32.8, 42.1, 46.8, 56.6, 62.3, 100.2, 109.3, 123.9, 124.2, 124.8, 125.1, 129.3, 129.3, 129.5, 141.8, 143.0, 166.8, 176.9, 191.6 ppm; Anal. Calcd. for $C_{22}H_{22}N_2O_4S_2$: C, 59.71; H, 5.01; N, 6.33; Found: C, 59.63; H, 5.11; N, 6.21 %

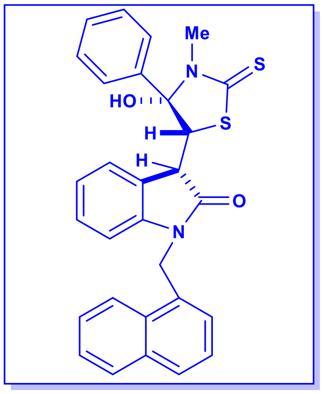

3-(4-hydroxy-3-methyl-4-phenyl-2-thioxothiazolidin-5-yl)-1-(naphthalen-1-ylmethyl) indolin-2-one (3k)

white solid; yield (119 mg, 80%); m.p. 176-178 °C; ^{1}H NMR (500 MHz, $CDCl_3$) δ 3.11 (3H, s), 4.52 (1H, d, *J*= 5.0 Hz), 4.61 (1H, d, *J*= 5.0 Hz), 5.44 (1H, d, *J*= 10.0 Hz), 5.57 (1H, d, *J*= 10.0 Hz), 6.74(1H, d, *J*= 10.0 Hz), 7.05-7.21 (3H,m), 7.44-7.69 (9H, m), 7.84 (1H, d, *J*= 10.0 Hz), 7.94 (1H, d, *J*= 10.0 Hz), 8.11 (1H, d, *J*= 10.0 Hz) 8.22 (1H, s) ppm; ^{13}C NMR (125 MHz, $CDCl_3$) δ 32.8, 42.7, 47.2, 56.9, 100.3, 110.6, 122.6, 123.7, 124.0, 124.7, 124.8, 125.5, 126.0, 126.1, 126.9, 128.6, 129.1, 129.2, 129.3, 129.4, 129.5, 130.9, 133.9, 141.8, 143.8, 177.1, 191.3 ppm; Anal. Calcd. for $C_{29}H_{24}N_2O_2S_2$: C, 70.13; H, 4.87; N, 5.64; Found: C, 69.97; H, 5.04; N, 5.49%

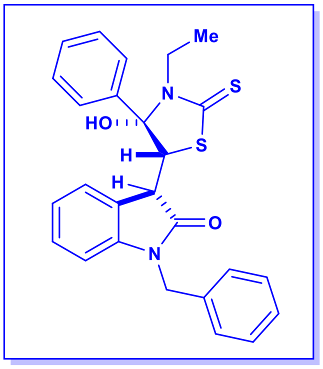

1-benzyl-3-(3-ethyl-4-hydroxy-4-phenyl-2-thioxothiazolidin-5-yl) indolin-2-one (3l)

white solid; yield (114 mg, 83 %); m.p. 152-154 °C; ^{1}H NMR (500 MHz, $CDCl_3$) δ 1.29 (3H, t, *J*= 5.0 Hz), 3.35 (1H, q, *J*= 10.0 Hz), 3.94 (1H, q, *J*= 5.0 Hz), 4.42 (1H, d, *J*= 5.0 Hz), 4.52 (1H, d, *J*= 5.0 Hz), 4.79 (1H, d, *J*= 15.0 Hz), 5.25 (1H, d, *J*= 15.0 Hz), 6.82 (1H, d, *J*= 10.0 Hz), 7.06 (2H, t, *J*= 5.0 Hz), 7.18 -7.65 (11H, m), 8.26 (1H, s) ppm; ^{13}C NMR (125 MHz, $CDCl_3$) δ 13.4, 42.3, 44.7, 47.1, 57.0, 100.9, 110.3, 123.7, 123.8, 124.8, 125.5, 127.6, 128.1, 129.1, 129.2, 129.3, 129.4, 134.5, 142.6, 143.6, 177.0, 190.8 ppm; Anal. Calcd. for $C_{26}H_{24}N_2O_2S_2$: C, 67.80; H, 5.25; N, 6.08; Found: C, 67.61; H, 5.37; N, 5.93 %.

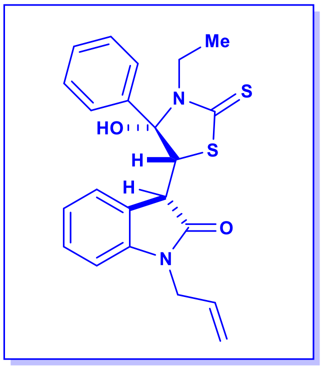

1-allyl-3-(3-ethyl-4-hydroxy-4-phenyl-2-thioxothiazolidin-5-yl) indolin-2-one (3m)

white solid; yield (93 mg, 76 %); m.p. 136-138 °C; ^{1}H NMR (500 MHz, $CDCl_3$) δ 1.26 (3H, t, *J*= 10.0 Hz), 3.32 (1H, q, *J*= 10.0 Hz), 3.90 (1H, q, *J*= 10.0 Hz), 4.37 (1H, d.d, *J*= 5.0 Hz), 4.41 (1H, d, *J*= 5.0 Hz), 4.46 (1H, d.d, *J*= 5.0 Hz), 4.51 (1H, d, *J*= 5.0 Hz), 5.33 (2H, quintet, *J*= 15.0 Hz), 5.85 (1H, m), 6.92 (1H, d, *J*= 10.0 Hz), 7.09 (2H, t, *J*= 5.0 Hz), 7.34 -7.62 (6H, m), 8.20 (1H, s) ppm; ^{13}C NMR (125 MHz, $CDCl_3$) δ 13.4, 42.3, 43.1, 47.0, 56.9, 100.9, 110.1, 118.5, 123.7, 123.8, 124.8, 125.4, 129.1, 129.3, 129.4, 130.2, 142.6, 157.5, 176.5, 190.8 ppm; Anal. Calcd. for $C_{22}H_{22}N_2O_2S_2$: C, 64.36; H, 5.40; N, 6.82; Found: C, 64.27; H, 5.51; N, 6.68 %.

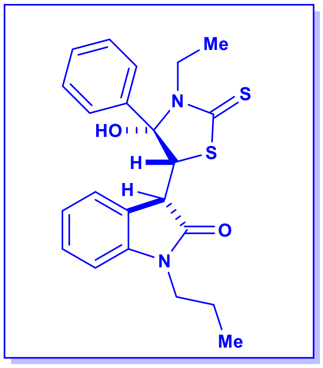

3-(3-ethyl-4-hydroxy-4-phenyl-2-thioxothiazolidin-5-yl)-1-propylindolin-2-one (3n)

white solid; yield (91 mg, 74 %); m.p. 140-142 °C; ^{1}H NMR (500 MHz, $CDCl_3$) δ 1.04 (3H, t, *J*= 10.0 Hz), 1.26 (3H, t, *J*= 10.0 Hz), 1.79 (2H, m), 3.32 (1H, m), 3.72 (1H, m), 3.89 (2H, m), 4.39 (1H, d, *J*= 5.0 Hz), 4.41 (1H, d, *J*= 5.0 Hz), 6.95 (1H, d, *J*= 10.0 Hz), 7.07 (2H, t, *J*= 5.0 Hz), 7.26 -7.62 (6H, m), 8.34 (1H, s) ppm; ^{13}C NMR (125 MHz, $CDCl_3$) δ 11.6, 13.4, 20.8, 40.2, 42.6, 47.0, 56.9, 100.9, 109.6, 123.6, 123.8, 124.8, 125.6, 129.1, 129.2, 129.4, 142.7, 144.0, 176.7, 190.9 ppm; Anal. Calcd. for $C_{22}H_{24}N_2O_2S_2$: C, 64.15; H, 5.86; N, 6.79; Found: C, 63.81; H, 5.92; N, 6.58 %

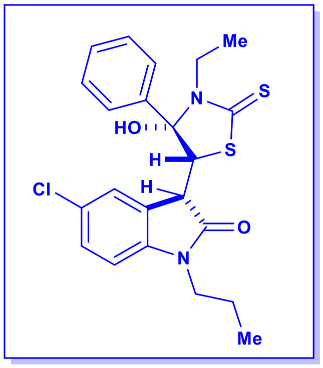

5-chloro-3-(3-ethyl-4-hydroxy-4-phenyl-2-thioxothiazolidin-5-yl)-1-propylindolin-2-one (3o)

white solid; yield (114 mg, 85 %); m.p. 134-136 °C; ^{1}H NMR (500 MHz, CDCl$_3$) δ 1.03 (3H, t, *J*= 10.0 Hz), 1.25 (3H, t, *J*= 10.0 Hz), 1.78 (2H, m), 3.30 (1H, t, *J*= 5.0 Hz), 3.68 (1H, t, *J*= 5.0 Hz), 3.88 (2H, m), 4.32 (1H, , d, *J*= 5.0 Hz), 4.42 (1H, d, *J*= 5.0 Hz), 6.88 (1H, d, *J*= 10.0 Hz), 7.07 (1H, s), 7.32 -7.60 (6H, m), 8.12 (1H, s) ppm; ^{13}C NMR (125 MHz, CDCl$_3$) δ 11.5, 13.3, 20.8, 42.3, 42.7, 47.0, 56.7, 100.9, 110.5, 121.4, 124.3, 124.7, 129.2, 129.4, 142.4, 156.2, 176.3, 190.5 ppm; Anal. Calcd. for $C_{22}H_{23}ClN_2O_2S_2$: C, 59.11; H, 5.19; N, 6.27; Found: C, 58.93; H, 5.24; N, 6.12 %

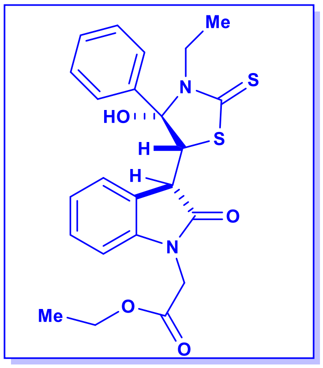

ethyl 2-(3-(3-ethyl-4-hydroxy-4-phenyl-2-thioxothiazolidin-5-yl)-2-oxoindolin-1-yl)acetate (3p)

white solid; yield (103 mg, 75 %); m.p. 138-140 °C; ^{1}H NMR (500 MHz, CDCl$_3$) δ 1.26 (3H, t, *J*= 5.0 Hz), 1.32 (3H, t, *J*= 5.0 Hz), 3.30 (1H, q, *J*= 5.0 Hz), 3.91 (1H, q, *J*= 5.0 Hz), 4.28 (2H, q, *J*= 5.0 Hz), 4.40 (1H, d, *J*= 15.0 Hz), 4.44 (1H, d, *J*= 5.0 Hz), 4.53 (1H, d, *J*= 5.0 Hz), 4.73 (1H, d, *J*= 15.0 Hz), 6.83 (1H, d, *J*= 10.0 Hz), 7.10 (2H, t, *J*= 5.0 Hz), 7.33 -7.61 (6H, m), 7.91 (1H, s) ppm; ^{13}C NMR (125 MHz, CDCl$_3$) δ 13.4, 14.3, 42.1, 42.3, 47.0, 56.6, 62.3, 100.9, 109.3, 123.9, 124.1, 124.8, 125.1, 129.2, 129.3, 129.5, 142.6, 143.1, 166.9, 176.9, 191.0 ppm; Anal. Calcd. for $C_{23}H_{24}N_2O_4S_2$: C, 60.51; H, 5.30; N, 6.14; Found: C, 60.38; H, 5.42; N, 5.97 %

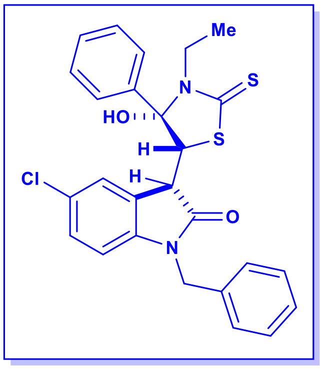

1-benzyl-5-chloro-3-(3-ethyl-4-hydroxy-4-phenyl-2-thioxothiazolidin-5-yl) indolin-2-one (3q)

white solid; yield (121 mg, 82 %); m.p. 198-200 °C; ^{1}H NMR (500 MHz, $CDCl_3$) δ 1.57 (3H, t, *J*= 5.0 Hz), 3.53 (2H, q, *J*= 10.0 Hz), 3.94 (1H, d, *J*= 5.0 Hz), 4.16 (1H, d, *J*= 5.0 Hz), 4.99 (2H, s), 6.66 (1H, d, *J*= 10.0 Hz), 7.14 (1H, d, *J*= 5.0 Hz), 7.27 -7.63 (10H, m), 8.02 (1H,s) 8.04 (1H, s) ppm; ^{13}C NMR (125 MHz, $CDCl_3$) δ 39.9, 41.3, 44.2, 110.0, 125.0, 127.3, 127.8, 127.9, 128.0, 128.3, 128.3, 128.8, 129.0, 130.8, 133.7, 135.5, 142.1, 177.4, 196.5 ppm; Anal. Calcd. for $C_{26}H_{23}ClN_2O_2S_2$: C, 63.08; H, 4.68; N, 5.66; Found: C, 62.81; H, 4.76; N, 5.41 %

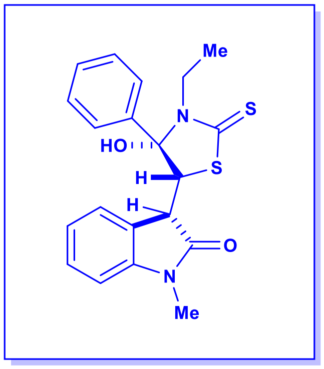

3-(3-ethyl-4-hydroxy-4-phenyl-2-thioxothiazolidin-5-yl)-1-methylindolin-2-one (3r)

white solid; yield (106 mg, 92 %); m.p. 140-142 °C; ^{1}H NMR (500 MHz, $CDCl_3$) δ 1.27 (3H, t, *J*= 5.0 Hz), 3.30 (1H, m), 3.34 (3H, s), 3.90 (1H, m), 4.42 (1H, d, *J*= 5.0 Hz), 4.43 (1H, d, *J*= 5.0 Hz), 6.94 (1H, d, *J*= 10.0 Hz), 7.09 (2H, m), 7.38 -7.62 (6H, m), 8.30 (1H, s) ppm; ^{13}C NMR (125 MHz, $CDCl_3$) δ 13.4, 27.0, 42.3, 47.1, 56.7, 101.0, 109.3, 123.6, 123.8, 124.8, 125.3, 129.1, 129.3, 129.5, 142.8, 144.3, 176.7, 190.9 ppm; Anal. Calcd. for $C_{20}H_{20}N_2O_2S_2$: C, 62.47; H, 5.24; N, 7.29; Found: C, 62.21; H, 5.33; N, 7.10 %

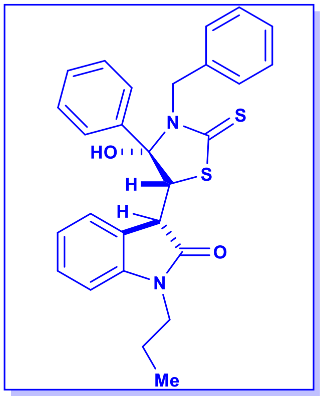

3-(-3-benzyl-4-hydroxy-4-phenyl-2-thioxothiazolidin-5-yl)-1-propylindolin-2-one (3s)

white solid; yield (125 mg, 88 %); m.p. 130-132 °C; ^{1}H NMR (500 MHz, $CDCl_3$) δ 1.07 (3H, t, *J*= 10.0 Hz), 1.81 (2H, m), 3.70 (1H, t, *J*= 10.0 Hz), 3.87(1H, t, *J*= 10.0 Hz), 4.36 (1H, d, *J*= 5.0 Hz), 4.48 (1H, d, *J*= 5.0 Hz), 4.52 (1H, d, *J*= 10.0 Hz), 5.12 (1H, d, *J*= 10.0 Hz), 6.95 (1H, d, *J*= 10.0 Hz), 7.07 (2H, t, *J*= 5.0 Hz), 7.20 -7.59 (11H, m), 8.25 (1H, s) ppm; ^{13}C NMR (125 MHz, $CDCl_3$) δ 11.7, 20.9, 42.6, 46.7, 50.2, 57.0, 101.0, 109.6, 123.6, 123.8, 125.0, 125.5, 127.0, 128.0, 128.4, 129.0, 129.2, 129.5, 136.7, 142.6, 143.9, 176.5, 193.0 ppm; Anal. Calcd. for $C_{27}H_{26}N_2O_2S_2$: C, 68.33; H, 5.52; N, 5.90; Found: C, 68.10; H, 5.61; N, 5.76 %.

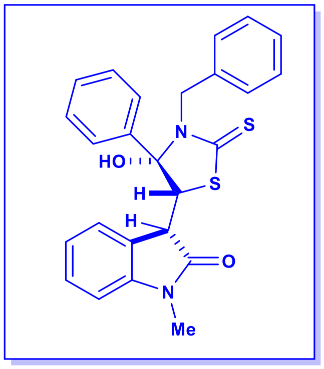

3-(3-benzyl-4-hydroxy-4-phenyl-2-thioxothiazolidin-5-yl)-1-methylindolin-2-one (3t)

white solid; yield (122 mg, 91 %); m.p. 140-142 °C; ^{1}H NMR (500 MHz, $CDCl_3$) δ 3.34 (3H, s), 4.38 (1H, d, , *J*= 5.0 Hz), 4.48 (1H, d, *J*= 10.0 Hz), 4.51 (1H, d, *J*= 5.0 Hz), 5.14 (1H, d, *J*= 10.0 Hz), 6.93 (1H, d, *J*= 10.0 Hz), 7.09 (2H, t, *J*= 5.0 Hz), 7.21-7.59 (11H, m), 8.17 (1H, s) ppm; ^{13}C NMR (125 MHz, $CDCl_3$) δ 27.1, 46.7, 50.3, 56.8, 101.1, 109.3, 123.7, 123.8, 125.0, 127.0, 128.0, 128.3, 129.0, 129.2, 129.6, 132.0, 136.6, 142.7, 144.3, 176.5, 193.1 ppm; Anal. Calcd. for $C_{25}H_{22}N_2O_2S_2$: C, 67.24; H, 4.97; N, 6.27; Found: C, 66.98; H, 5.11; N, 6.03 %.

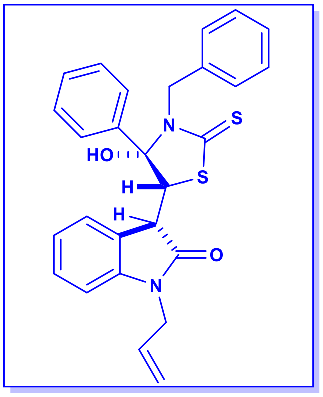

1-allyl-3-(3-benzyl-4-hydroxy-4-phenyl-2-thioxothiazolidin-5-yl) indolin-2-one (3u)

white solid; yield (123 mg, 87 %); m.p. 132-134°C; ^{1}H NMR (500 MHz, $CDCl_3$) δ 4.41 (2H, d, *J*= 5.0 Hz), 4.48 (1H, d.d, *J*= 5.0 Hz), 4.50 (1H, d, *J*= 5.0 Hz), 4.52 (1H, d.d, *J*= 5.0 Hz), 5.14 (1H, d, *J*= 10.0 Hz), 5.35 (2H, quintet, *J*= 10.0 Hz), 5.90 (1H, m), 6.93 (1H, d, *J*= 5.0 Hz), 7.08 (2H, t, *J*= 5.0 Hz), 7.20 -7.60 (11H, m), 8.09 (1H, s) ppm; ^{13}C NMR (125 MHz, $CDCl_3$) δ 43.1, 46.7, 50.2, 57.0, 101.0, 110.2, 118.6, 123.7, 125.0, 125.3, 127.0, 128.0 , 128.4, 129.0, 129.2, 129.4, 130.4, 136.6, 142.5, 143.5, 176.3, 193.0 ppm; Anal. Calcd. for $C_{27}H_{24}N_2O_2S_2$: C, 68.62; H, 5.12; N, 5.93; Found: C, 68.43; H, 5.26; N, 5.78 %.

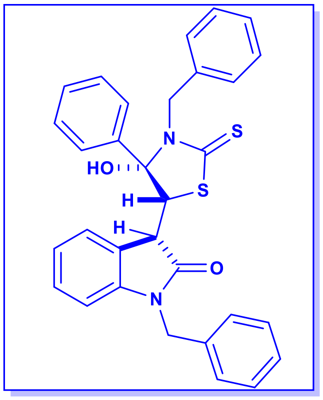

1-benzyl-3-(3-benzyl-4-hydroxy-4-phenyl-2-thioxothiazolidin-5-yl) indolin-2-one (3v)

white solid; yield (124 mg, 79 %); m.p. 154-156 °C; ^{1}H NMR (500 MHz, $CDCl_3$) δ 4.46 (1H, d, *J*= 5.0 Hz), 4.49 (1H, d, *J*= 5.0 Hz), 4.51 (1H, d, *J*= 5.0 Hz), 4.80 (1H, d, *J*= 10.0 Hz), 5.20 (2H, d, *J*= 10.0 Hz), 6.82 (1H, d, *J*= 10.0 Hz), 7.05 (2H, t, *J*= 5.0 Hz), 7.19 -7.62 (16H, m), 8.10 (1H, s) ppm; ^{13}C NMR (125 MHz, $CDCl_3$) δ 44.7, 46.8, 50.3, 57.0, 101.1, 110.2, 123.7, 123.8, 124.9, 125.4, 127.0, 127.6, 128.0, 128.1, 128.5, 129.0, 129.2, 129.2, 129.4, 134.7, 136.7, 142.6, 143.5, 176.7, 193.0 ppm; Anal. Calcd. for $C_{31}H_{26}N_2O_2S_2$: C, 71.24; H, 5.01; N, 5.36; Found: C, 71.02; H, 5.12; N, 5.10 %.

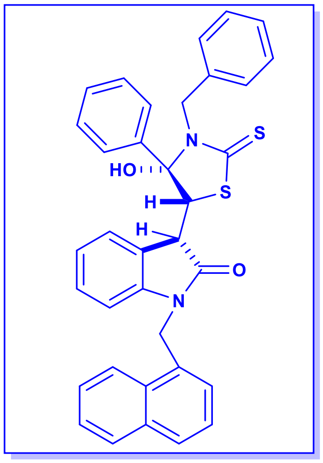

3-(3-benzyl-4-hydroxy-4-phenyl-2-thioxothiazolidin-5-yl)-1-(naphthalen-1-ylmethyl) indolin-2-one (3w)

white solid; yield (132 mg, 77%); m.p. 140-142 °C; ^{1}H NMR (500 MHz, $CDCl_3$) δ 4.49 (1H, d, *J*= 5.0 Hz), 4.52 (1H, d, *J*= 5.0 Hz), 4.56 (1H, d, *J*= 10.0 Hz), 5.19 (1H, d, *J*= 10.0 Hz), 5.43 (1H, d, *J*= 10.0 Hz), 5.58 (1H, d, *J*= 10.0 Hz), 6.75(1H, d, *J*= 10.0 Hz), 7.05-7.69 (17H, m), 7.86 (1H, d, *J*= 10.0 Hz), 7.95 (1H, d, *J*= 10.0 Hz), 8.14 (1H, d, *J*= 10.0 Hz) 8.16 (1H, s) ppm; ^{13}C NMR (125 MHz, $CDCl_3$) δ 42.8, 47.0, 50.3, 56.9, 100.0, 101.1, 110.6, 122.7, 123.7, 123.9, 124.9, 125.0, 125.9, 126.2, 127.0, 127.0, 128.0, 128.3, 128.7, 129.1, 129.2, 129.3, 129.3, 129.5, 130.9, 133.9, 136.6, 142.6, 143.8, 176.9, 192.9 pm; Anal. Calcd. for $C_{35}H_{28}N_2O_2S_2$: C, 73.40; H, 4.93; N, 4.89; Found: C, 73.27; H, 5.09; N, 4.71 %

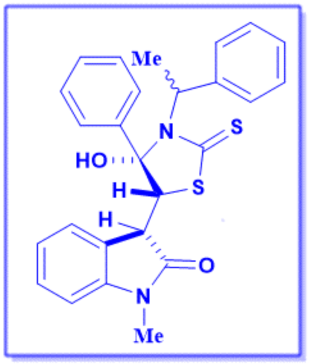

4-hydroxy-4-phenyl-3-(1-phenylethyl)-2-thioxothiazolidin-5-yl)-1-methylindolin-2-one (3x)

white solid; yield (78 mg, 57 %); ^{1}H NMR (500 MHz, $CDCl_3$) δ 3.02 (3H, s), 3.33 (3H, d, *J*= 5.0 Hz), 3.91 (1H, q, *J*= 5.0 Hz), 5.94 (1H, m), 6.25 (1H, d, *J*= 5.0 Hz), 6.52 (1H, d, *J*= 5.0 Hz), 6.85 (1H, d, *J*= 10.0 Hz), 7.01 (1H, t, *J*= 5.0 Hz), 7.14 (2H, m), 7.37 (2H, t, *J*= 10.0 Hz), 7.46 (4H, m), 7.67 (1H, d, *J*= 5.0 Hz), 7.80 (1H, d, *J*= 10.0 Hz), 7.87 (1H, d, *J*= 5.0 Hz) 7.90 (1H, s) ppm; ^{13}C NMR (125 MHz, $CDCl_3$) δ 26.0, 26.8, 46.1, 52.8, 56.9, 65.8, 86.5, 107.3, 108.7, 121.6, 123.7, 126.2, 127.7, 128.5, 128.7, 133.6, 135.4, 137.9, 143.2, 143.9, 177.3, 193.7.

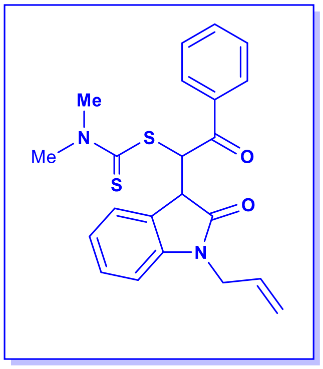

1-(1-allyl-2-oxoindolin-3-yl)-2-oxo-2-phenylethyl dimethylcarbamodithioate (4a)

white solid; yield (102 mg, 83 %); m.p. 114-116 °C; ^{1}H NMR (500 MHz, $CDCl_3$) δ 3.38 (3H, s), 3.62 (3H, s), 3.92 (1H, d, *J*= 5.0 Hz), 4.47 (2H, d.d, *J*= 5.0 Hz), 5.35 (2H, d.d, *J*= 5.0 Hz), 5.97 (1H, quintet, *J*= 5.0 Hz), 6.84 (2H, d, *J*= 5.0 Hz), 7.01 (1H, d, *J*= 5.0 Hz), 7.25 (1H, d, *J*= 5.0 Hz), 7.43 -7.58 (4H, m), 8.02 (2H, d , *J*= 10.0 Hz) ppm; ^{13}C NMR (125 MHz, $CDCl_3$) δ 35.2, 42.5, 46.2, 57.5, 109.2, 117.8, 122.9, 126.6, 127.8, 128.5, 128.7, 128.9, 131.2, 131.7, 132.6, 133.9, 136.4, 137.7, 176.1, 191.3, 194.1 ppm; Anal. Calcd. for $C_{22}H_{22}N_2O_2S_2$: C, 64.36; H, 5.40; N, 6.82; Found: C, 64.24; H, 5.48; N, 6.67 %.

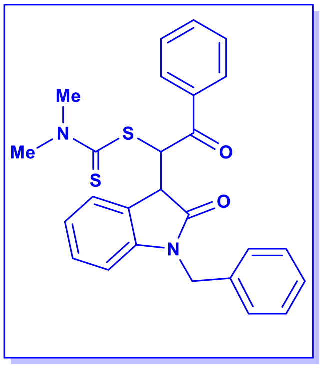

1-(1-benzyl-2-oxoindolin-3-yl)-2-oxo-2-phenylethyl dimethylcarbamodithioate (4b)

white solid; yield (102 mg, 74 %); m.p. 140-142 °C; ^{1}H NMR (500 MHz, $CDCl_3$) δ 3.38 (3H, s), 3.63 (3H, s), 4.01 (1H, d, *J*= 5.0 Hz), 5.05 (2H, d, *J*= 10.0 Hz), 6.71 (1H, d, *J*= 5.0 Hz), 6.88 (1H, d, *J*= 5.0 Hz), 6.98 (1H, t, *J*= 5.0 Hz), 7.16 (1H, t, *J*= 5.0 Hz), 7.34 -7.59 (9H, m), 8.05 (2H, d , *J*= 10.0 Hz) ppm; ^{13}C NMR (125 MHz, $CDCl_3$) δ 41.7, 44.1, 46.3, 46.5, 57.5, 94.4, 109.1, 122.4, 123.9, 126.5, 127.5, 127.6, 128.5, 128.7, 129.0, 133.6, 134.9, 136.2, 143.6, 176.3, 194.2, 194.2 ppm; Anal. Calcd. for $C_{26}H_{24}N_2O_2S_2$: C, 67.80; H, 5.25; N, 6.08; Found: C, 67.57; H, 5.37; N, 5.92%.

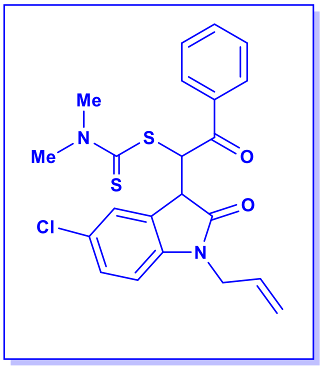

1-(1-allyl-5-chloro-2-oxoindolin-3-yl)-2-oxo-2-phenylethyl dimethylcarbamodithioate (4c)

white solid; yield (108 mg, 81 %); m.p. 110-112 °C; ^{1}H NMR (500 MHz, $CDCl_3$) δ 3.39 (3H, s), 3.64 (3H, s), 3.90 (1H, d, *J*= 5.0 Hz), 4.45 (2H, d.d, *J*= 5.0 Hz), 5.34 (2H, d.d, *J*= 5.0 Hz), 5.95 (1H, quintet, *J*= 5.0 Hz), 6.79 (2H, d, *J*= 5.0 Hz), 7.20 (1H,d, *J*= 5.0 Hz), 7.45 -7.60 (4H, m), 7.97 (2H, d , *J*= 10.0 Hz) ppm; ^{13}C NMR (125 MHz, $CDCl_3$) δ 41.6, 42.6, 46.2, 46.6, 57.5, 100.0, 109.9, 117.8, 124.2, 127.9, 128.4, 128.9, 129.3, 131.3, 132.2, 133.8, 134.7, 164.6, 184.5, 193.9 ppm; Anal. Calcd. for $C_{22}H_{21}ClN_2O_2S_2$: C, 59.38; H, 4.76; N, 6.30; Found: C, 59.22; H, 4.84; N, 6.21 %.

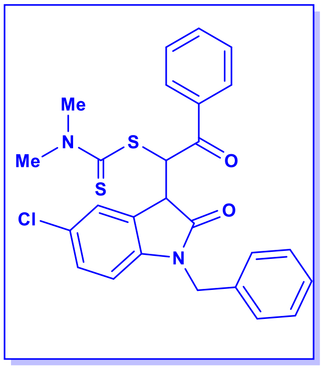

1-(1-benzyl-5-chloro-2-oxoindolin-3-yl)-2-oxo-2-phenylethyl dimethylcarbamodithioate (4d)

white solid; yield (119 mg, 80 %); m.p. 132-134 °C; ^{1}H NMR (500 MHz, $CDCl_3$) δ 3.39 (3H, s), 3.99 (3H, s), 3.99 (1H, d, *J*= 5.0 Hz), 5.03 (2H, d, *J*= 10.0 Hz), 6.61 (1H, d, *J*= 5.0 Hz), 6.85 (1H, d, *J*= 5.0 Hz), 7.12(1H, d, *J*= 5.0 Hz), 7.35 -7.60 (9H, m), 8.01 (2H, d , *J*= 10.0 Hz) ppm; ^{13}C NMR (125 MHz, $CDCl_3$) δ 41.7, 44.2, 46.4, 46.7, 57.5, 110.0, 124.3, 127.5, 127.7, 128.4, 128.8, 128.8, 129.0, 133.8, 134.6, 135.8, 142.2, 176.0, 193.9, 194.0 ppm; Anal. Calcd. for $C_{26}H_{23}ClN_2O_2S_2$: C, 63.08; H, 4.68; N, 5.66; Found: C, 62.81; H, 4.81; N, 5.49 %.

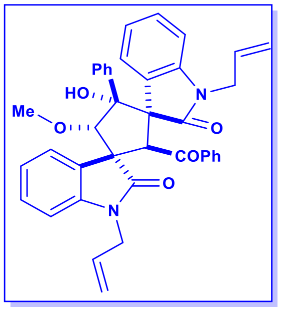

1,1''-diallyl-2'-benzoyl-4'-hydroxy-5'-methoxy-4'-phenyldispiro[indoline-3,1'-cyclopentane-3',3''-indoline]-2,2''-dione (5a)

white solid; yield (79 mg, 86 %); m.p. 258-260 °C; ^{1}H NMR (500 MHz, DMSO-d$_6$) δ 2.95 (3H, s), 3.93 (2H, d.d, *J*= 15.0 Hz), 4.21 (1H, d, *J*= 15.0 Hz), 4.53 (2H, d.d, *J*= 15.0 Hz), 4.80 (1H, d, *J*= 15.0 Hz), 5.14 (1H, quintet, *J*= 10.0 Hz), 5.21 (2H, t, *J*= 15.0 Hz), 5.27 (1H, *s*), 5.77 (1H, quintet, *J*= 10.0 Hz), 5.96 (1H, *s*), 6.57 (1H, d, *J*= 5.0 Hz), 6.63 (1H, d, *J*= 5.0 Hz), 6.68 (1H, s), 6.98 - 7.23 (13H, m), 7.374 (1H, t, *J*= 5.0 Hz), 7.86 (1H, d, *J*= 5.0 Hz), 8.06 (1H, d, *J*= 5.0 Hz) ppm; ^{13}C NMR (125 MHz, DMSO-d$_6$) δ 41.9, 43.1, 58.3, 60.0, 60.7, 61.6, 84.5, 89.3, 100.0, 108.8, 109.4, 116.9, 118.4, 122.0, 123.9, 125.0, 125.9, 126.2, 126.6, 127.3, 127.6, 127.9, 129.1, 130.6, 131.6, 131.9, 133.3, 136.8, 137.6, 142.5, 143.8, 176.3, 179.3, 196.6 ppm; Anal. Calcd. for $C_{39}H_{34}N_2O_5$: C, 76.70; H, 5.61; N, 4.59; Found: C, 76.51; H, 5.76; N, 4.34 %;

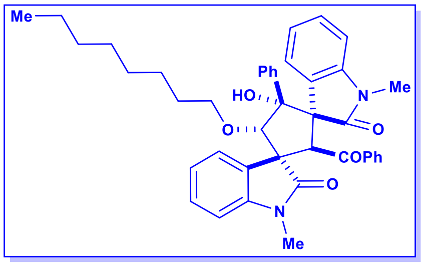

2'-benzoyl-4'-hydroxy-1,1''-dimethyl-5'-(octyloxy)-4'-phenyldispiro[indoline-3,1'-cyclopentane-3',3''-indoline]-2,2''-dione (5b)

white solid; yield (88 mg, 89 %); m.p. 222-224 °C; ^{1}H NMR (500 MHz, DMSO-d6) δ 0.79-1.19 (15H, m), 2.79 (3H,s), 2.85 (1H, t, *J* = 10.0 Hz), 3.08 (3H,s), 3.17 (1H, t, *J* = 10.0 Hz), 5.22 (1H, s), 5.99 (1H, s), 6.56 (1H, d, *J* = 5.0 Hz), 6.75 (2H, t, *J* = 5.0 Hz), 6.97 -7.49 (14H, m), 7.82 (1H, d, *J* = 5.0 Hz), 8.09 (1H, d, *J* = 5.0 Hz) ppm; ^{13}C NMR (125 MHz, DMSO-d6) δ 14.4, 22.5, 25.8, 26.2, 27.1, 28.9, 29.0, 29.6, 31.6, 58.5, 61.6, 64.6, 84.6, 87.5, 108.0, 108.8, 112.7, 122.0, 123.8, 125.7, 126.1, 126.4, 126.7, 127.2, 127.3, 127.8, 128.4, 129.1, 130.6, 131.6, 133.2, 136.8, 137.5, 143.3, 144.5, 176.6, 179.7, 196.6 ppm; Anal. Calcd. for $C_{42}H_{44}N_2O_5$: C, 76.80; H, 6.75; N, 4.27; Found: C, 76.64; H, 6.83; N, 4.08 %.

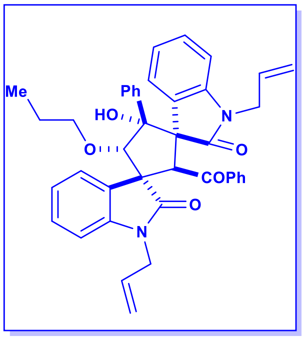

1,1''-diallyl-2'-benzoyl-4'-hydroxy-4'-phenyl-5'-propoxydispiro[indoline-3,1'-cyclopentane-3',3''-indoline]-2,2''-dione (5c)

white solid; yield (76 mg, 79 %); m.p. 166-168 °C; ^{1}H NMR (500 MHz, DMSO-d$_6$) δ 0.49 (3H, t, *J*= 10.0 Hz), 1.10 (2H, m), 2.88 (1H, q, *J*= 10.0 Hz), 3.13 (1H, q , *J*= 10.0 Hz), 3.97 (2H, d.d, *J*= 15.0 Hz), 4.21 (1H, d, *J*= 15.0 Hz), 4.51 (2H, d.d, *J*= 15.0 Hz), 5.81 (1H, d, *J*= 15.0 Hz), 5.16 (1H, quintet, *J*= 10.0 Hz), 5.22 (1H, t, *J*= 15.0 Hz), 5.28 (1H, t, *J*= 10.0 Hz), 5.75 (1H, quintet, *J*= 10.0 Hz), 6.04 (1H, *s*), 6.59 (1H, d, *J*= 5.0 Hz), 6.64 (1H, d, *J*= 5.0 Hz), 6.71 (1H, s), 6.98 -7.24 (13H, m), 7.37 (1H, t, *J*= 5.0 Hz), 7.86 (1H, d, *J*= 5.0 Hz), 8.04 (1H, d, *J*= 5.0 Hz) ppm; ^{13}C NMR (125 MHz, DMSO-d$_6$) δ 10.7, 22.9, 41.9, 43.2, 58.4, 61.6, 64.5, 73.8, 84.5, 87.9, 100.0, 101.0, 108.8, 109.3, 116.9, 119.0, 122.0, 123.8, 125.9, 126.2, 126.7, 127.3, 127.5, 127.8, 128.7, 129.1, 130.5, 131.5, 131.9, 133.2, 136.8, 137.6, 142.6, 143.7, 176.3, 179.4, 196.6 ppm; Anal. Calcd. for $C_{41}H_{38}N_2O_5$: C, 77.09; H, 6.00; N, 4.39; Found: C, 76.89; H, 6.14; N, 4.21 %

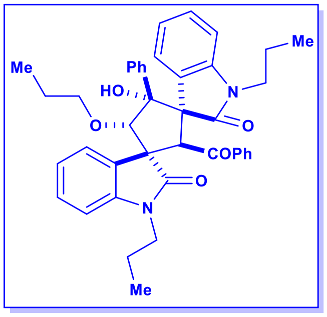

4'-hydroxy-2'-methyl-4'-phenyl-5'-propoxy-1,1''-dipropyldispiro[indoline-3,1'-cyclopentane-3',3''-indoline]-2,2''-dione (5d)

white solid; yield (72 mg, 75 %); m.p. 210-212 °C; ^{1}H NMR (500 MHz, DMSO-d$_6$) δ 0.49 (3H, t, *J*= 10.0 Hz), 0.55 (3H, t, *J* = 10.0 Hz), 0.93 (3H, t, *J*= 10.0 Hz), 1.00 (2H, m), 1.11 (2H, m), 1.47-1.66 (2H, m), 2.86-3.15 (2H, m), 3.20-3.38 (2H, m), 3.50-3.77 (2H, m), 5.23 (1H, s), 6.03 (1H, s), 6.66 (1H, d, *J* = 10.0 Hz), 6.76 (1H, s), 6.79 (2H, d, *J*= 10.0 Hz), 6.96 (2H, d, *J*= 5.0 Hz), 7.02-7.19 (9H, m), 7.25 (1H, t, *J*= 10.0 Hz), 7.36 (1H, t, *J*= 10.0 Hz), 7.84 (1H, d, *J*= 5.0 Hz), 8.04 (1H, d, *J*= 5.0 Hz) ppm; ^{13}C NMR (125 MHz, DMSO-d$_6$) δ 10.7, 11.6, 11.9, 20.4, 21.0, 23.0, 41.3, 42.5, 58.4, 61.5, 64.2, 73.8, 84.6, 87.9, 100.0, 108.2, 109.0, 121.7, 123.6, 125.9, 126.1, 126.6, 127.2, 127.4, 128.6, 129.1, 130.7, 133.1, 136.9, 137.7, 143.1, 144.4, 176.4, 179.6, 196.6 ppm; Anal. Calcd. for $C_{41}H_{42}N_2O_5$: C, 76.61; H, 6.59; N, 4.36; Found: C, 76.43; H, 5.71; N, 4.19

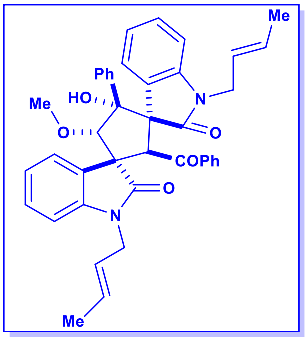

2'-benzoyl-1,1''-di(but-2-en-1-yl)-4'-hydroxy-5'-methoxy-4'-phenyldispiro[indoline-3,1'-cyclopentane-3',3''-indoline]-2,2''-dione (5e)

white solid; yield (79 mg, 83 %); m.p. 206-208 °C; ^{1}H NMR (500 MHz, DMSO-d$_6$) δ 1.41 (3H, d, *J*= 10.0 Hz), 1.65 (3H, d, *J*= 10.0 Hz), 2.93 (3H, t, *J*= 10.0 Hz), 3.80 (1H, d, *J*= 10.0 Hz), 3.98 (1H, d, *J*= 10.0 Hz), 4.19 (1H, d, *J*= 10.0 Hz), 4.41 (1H, d, *J*= 10.0 Hz), 4.46 (1H, m), 5.93 (1H, m), 5.26 (1H, *s*), 5.38 (1H, m), 5.72 (1H, m), 5.94 (1H, *s*), 6.54-6.67 (3H, m), 6.92 -7.24 (13H, m), 7.36 (1H, t, *J*= 5.0 Hz), 7.84 (1H, d, *J*= 5.0 Hz), 8.04 (1H, d, *J*= 5.0 Hz) ppm; ^{13}C NMR (125 MHz, DMSO-d$_6$) δ 13.4, 18.0, 36.6, 37.7, 41.0, 42.3, 58.2, 59.8, 61.4, 64.2, 84.5, 89.3, 108.7, 109.4, 121.9, 123.8, 124.2, 124.6, 125.9, 126.1, 126.7, 127.3, 127.5, 127.8, 128.6, 129.1, 129.8, 130.7 133.1, 136.8, 137.6, 142.5, 143.9, 176.1, 179.1, 196.5 ppm; Anal. Calcd. for $C_{41}H_{38}N_2O_5$: C, 77.09; H, 6.00; N, 4.39; Found: C, 76.88; H, 6.08; N, 4.19 %

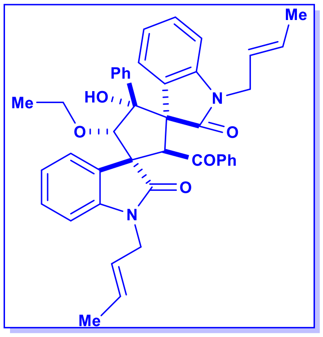

2'-benzoyl-1,1"-di((E)-but-2-en-1-yl)-5'-ethoxy-4'-hydroxy-4'-phenyldispiro[indoline-3,1'-cyclopentane-3',3"-indoline]-2,2"-dione (5f)

white solid; yield (79 mg, 81 %); m.p. 162-164 °C; ^{1}H NMR (500 MHz, DMSO-d$_6$) δ 0.72 (3H, t, *J*= 5.0 Hz), 1.41 (3H, d, *J*= 10.0 Hz), 1.64 (3H, d, *J*= 10.0 Hz), 2.95 (1H, q, *J*= 10.0 Hz), 3.23 (1H, q, *J*= 5.0 Hz), 3.80 (1H, d, *J*= 10.0 Hz), 3.95 (1H, d, *J*= 10.0 Hz), 4.19 (1H, d, *J*= 10.0 Hz), 4.42 (1H, d, *J*= 10.0 Hz), 4.4.67 (1H, m), 4.93 (1H, m), 5.26 (1H, *s*), 5.39 (1H, m), 5.73 (1H, m), 6.03 (1H, *s*), 6.56-6.69 (3H, m), 6.94 -7.24 (13H, m), 7.36 (1H, t, *J*= 5.0 Hz), 7.84 (1H, d, *J*= 5.0 Hz), 8.02 (1H, d, *J*= 5.0 Hz) ppm; ^{13}C NMR (125 MHz, DMSO-d$_6$) δ 13.5, 15.6, 18.0, 36.6, 37.6, 41.0, 42.2, 58.4, 61.4, 64.2, 67.6, 84.6, 87.8, 108.7, 109.3, 121.9, 123.7, 124.3, 124.6, 125.9, 126.1, 126.5, 126.7, 127.3, 127.5, 127.8, 128.6, 129.1, 129.8, 130.7 133.1, 136.8, 137.6, 142.6, 143.9, 176.1, 179.2, 196.6 ppm; Anal. Calcd. for $C_{42}H_{40}N_2O_5$: C, 77.28; H, 6.18; N, 4.29; Found: C, 77.05; H, 6.32; N, 4.10%

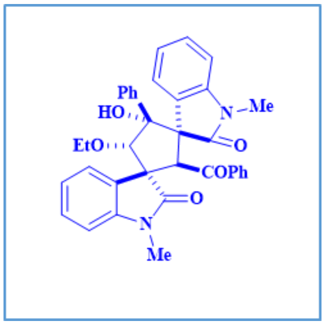

2'-benzoyl-5'-ethoxy-4'-hydroxy-4'-phenyl-1,1"-dimethyldispiro[indoline-3,1'-cyclopentane-3',3"-indoline]-2,2"-dione (5g)

white solid; yield (70 mg, 85%); m.p. 266-268 °C; ^{1}H NMR (500 MHz, DMSO-d$_6$) δ 0.69 (3H, t, *J*= 10.0 Hz), 2.79 (3H, s), 2.96 (1H, q, *J*= 10.0 Hz), 3.11 (3H, s), 3.19 (1H, q, *J*= 10.0 Hz), 5.23 (1H, s), 6.02 (1H, s), 6.58 (1H, d, *J* = 10.0 Hz), 6.75 (2H, d, *J* = 10.0 Hz), 6.90 -7.13 (10H, m), 7.18 (2H, t, *J*= 10.0 Hz), 7.26 (1H, t *J*= 10.0 Hz), 7.35 (1H, t, *J*= 10.0 Hz), 7.83 (1H, d, *J*= 5.0 Hz), 8.08(1H, d, *J*= 5.0 Hz) ppm; ^{13}C NMR (125 MHz, DMSO-d$_6$) δ 15.7, 26.2, 27.1, 58.6, 61.6, 64.6, 67.4, 84.6, 87.4, 108.0, 108.8, 121.9, 123.8, 125.7, 126.0, 126.4, 126.7, 127.2, 127.3, 127.8, 128.4, 129.1, 130.6, 133.1, 136.8, 137.5, 143.3, 144.6, 176.6, 179.7, 196.6 ppm.

4. ^{1}H NMR, ^{13}C NMR Spectra of Products 3a-3x, 4a-4d, 5a-5f.

^{1}H NMR (500 MHz, $CDCl_3$), ^{13}C NMR (125 MHz, $CDCl_3$)

1-allyl-3-(4-hydroxy-3-methyl-4-phenyl-2-thioxothiazolidin-5-yl) indolin-2-one (3a)

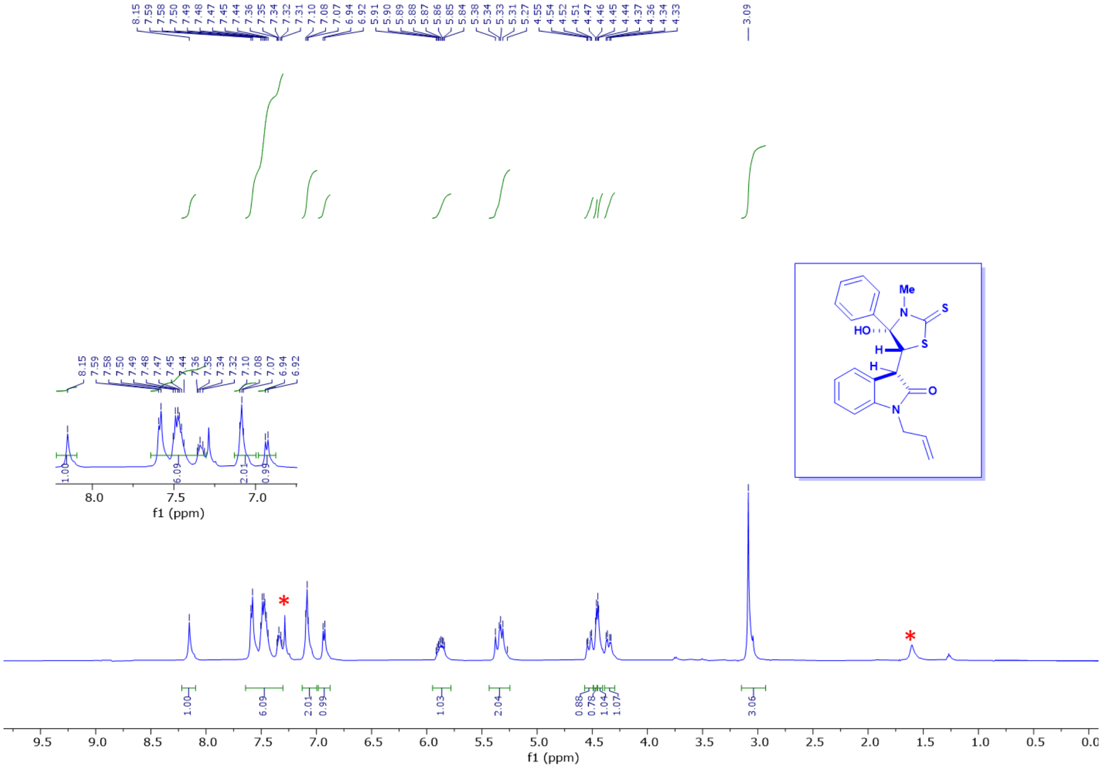

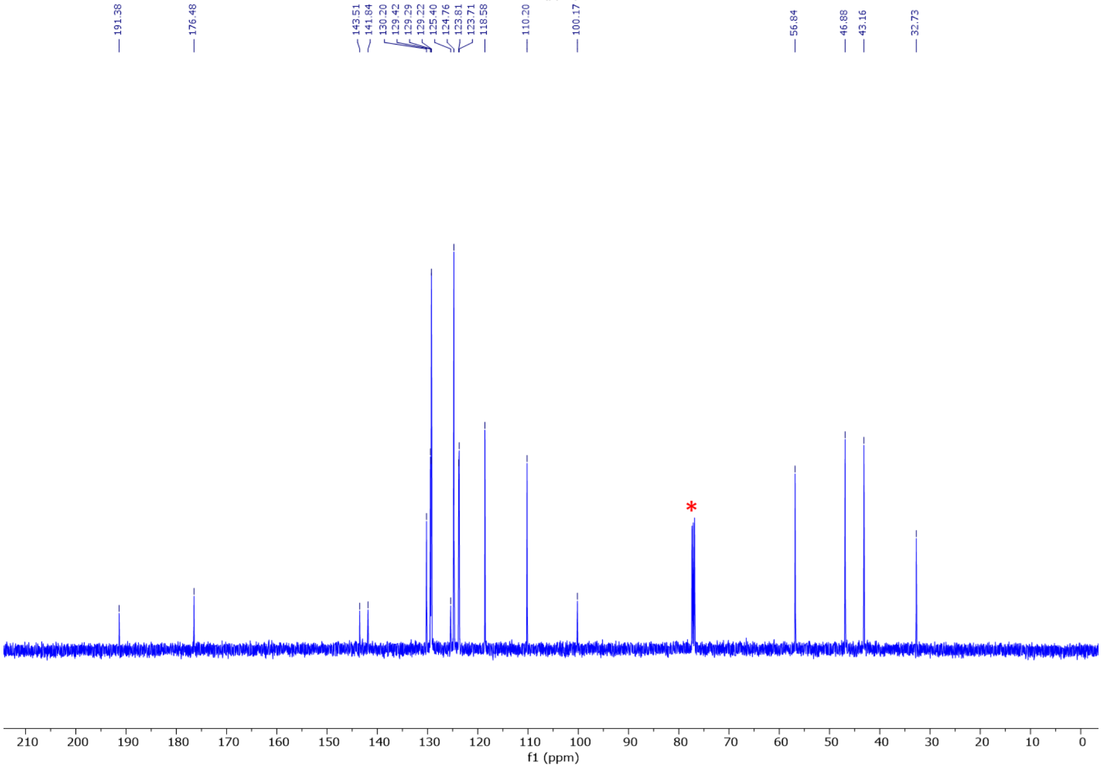

^{1}H NMR (500 MHz, $CDCl_3$), ^{13}C NMR (125 MHz, $CDCl_3$)

1-benzyl-3-(4-hydroxy-3-methyl-4-phenyl-2-thioxothiazolidin-5-yl) indolin-2-one (3b)

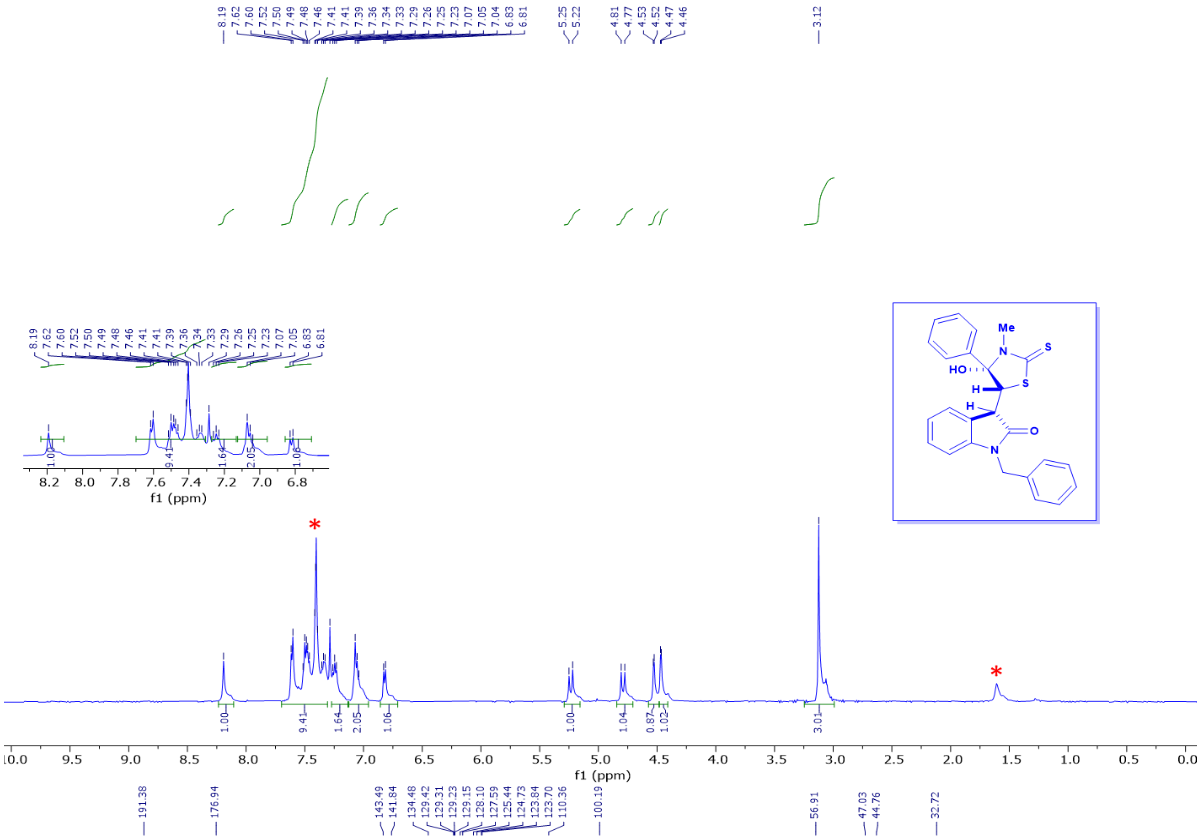

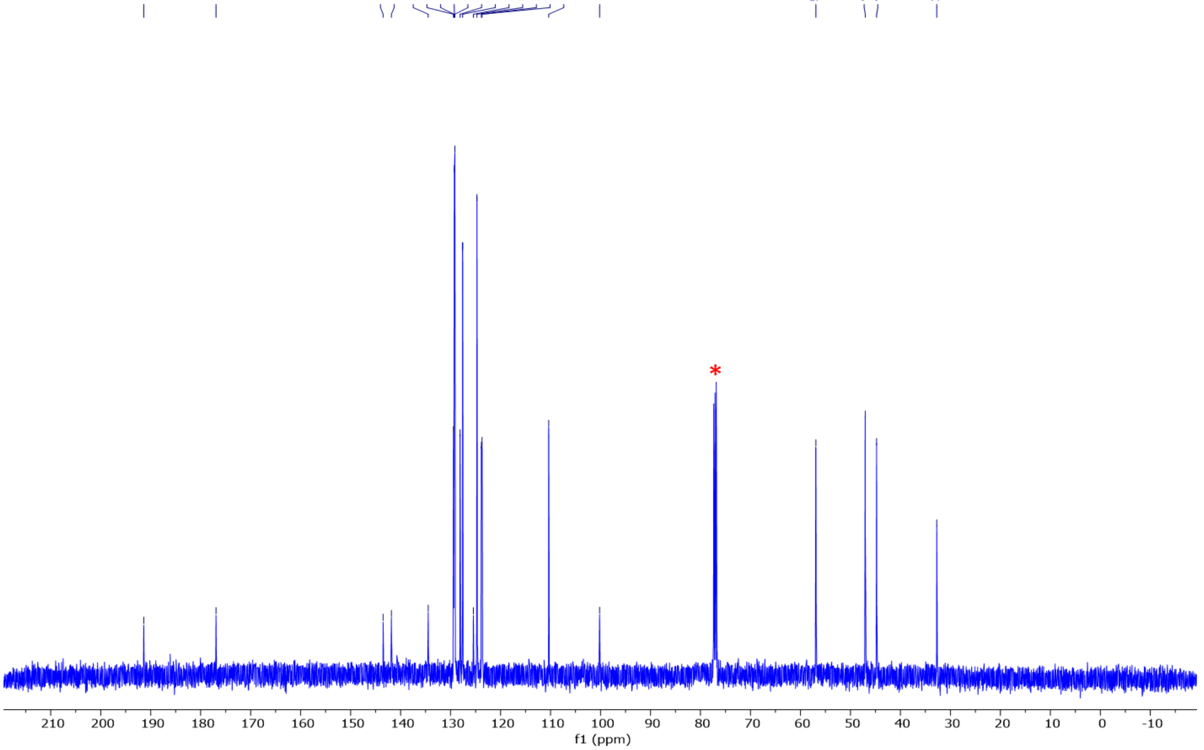

^{1}H NMR (500 MHz, $CDCl_3$), ^{13}C NMR (125 MHz, $CDCl_3$)

3-(4-hydroxy-3-methyl-4-phenyl-2-thioxothiazolidin-5-yl)-1-propylindolin-2-one (3c)

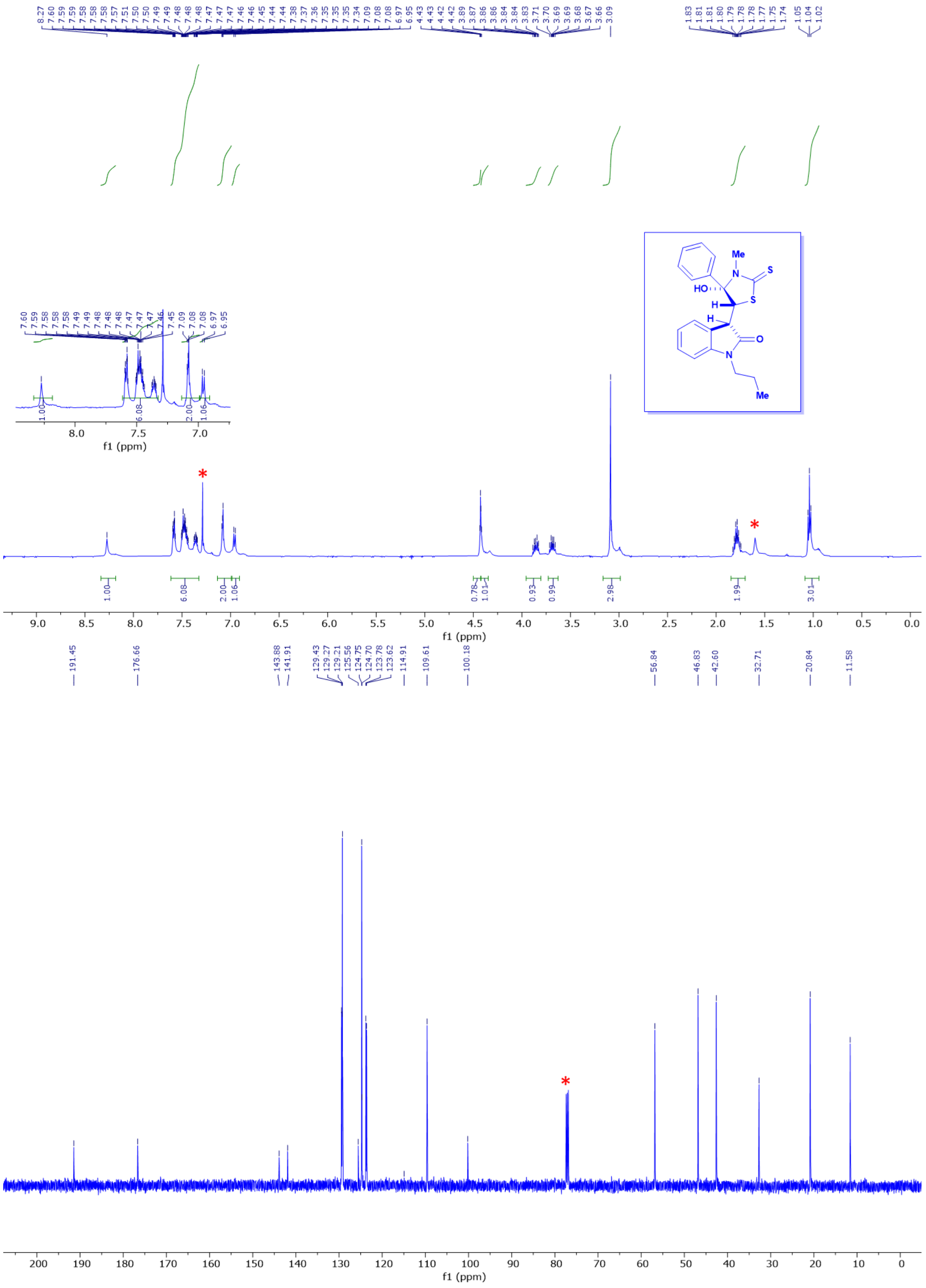

^{1}H NMR (500 MHz, $CDCl_3$), ^{13}C NMR (125 MHz, $CDCl_3$)

5-chloro-3-(4-hydroxy-3-methyl-4-phenyl-2-thioxothiazolidin-5-yl)-1-propylindolin-2-one (3d)

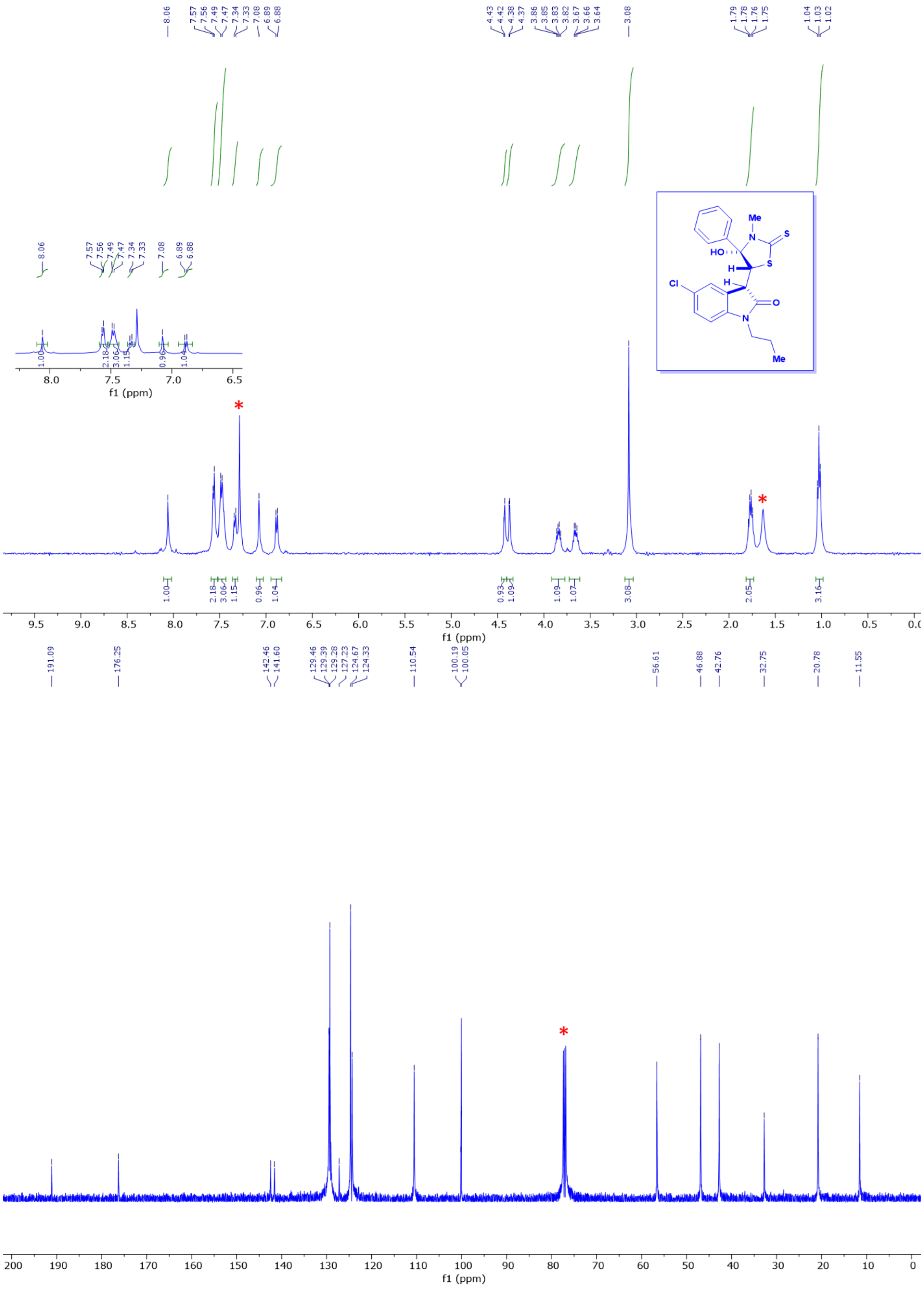

^{1}H NMR (500 MHz, $CDCl_3$), ^{13}C NMR (125 MHz, $CDCl_3$)

1-allyl-5-chloro-3-(4-hydroxy-3-methyl-4-phenyl-2-thioxothiazolidin-5-yl)indolin-2-one (3e)

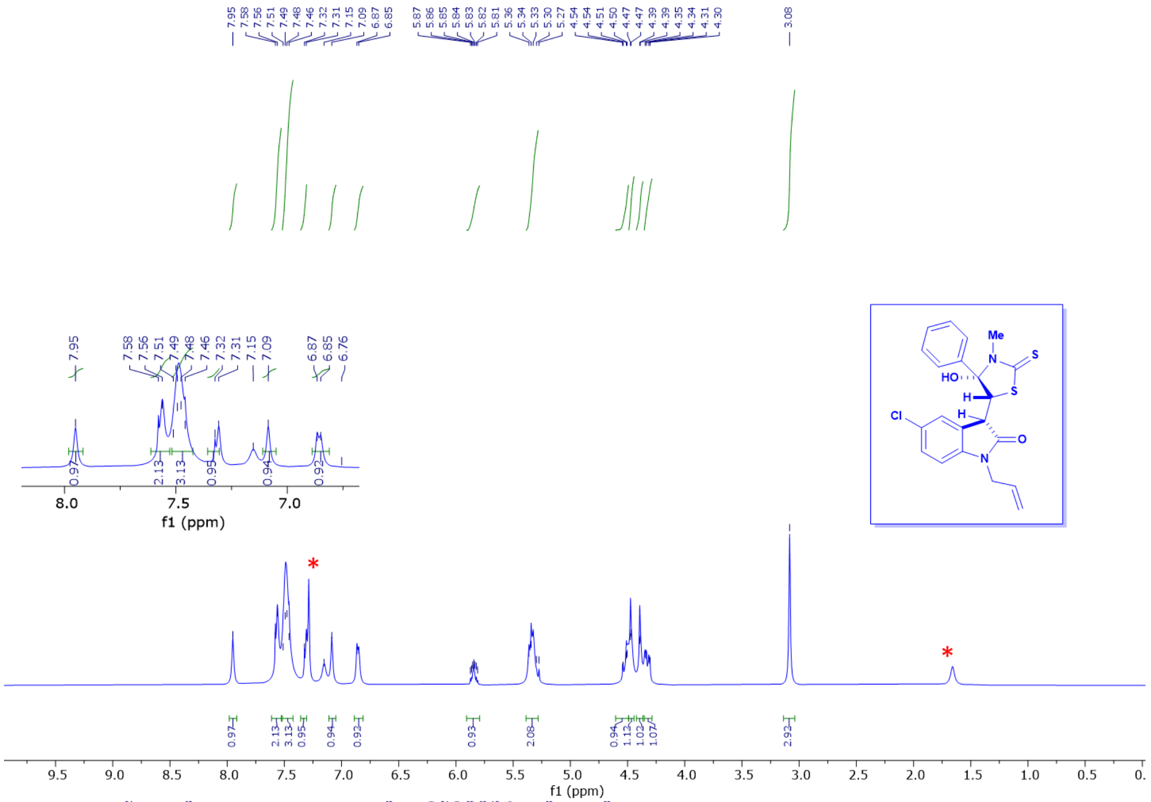

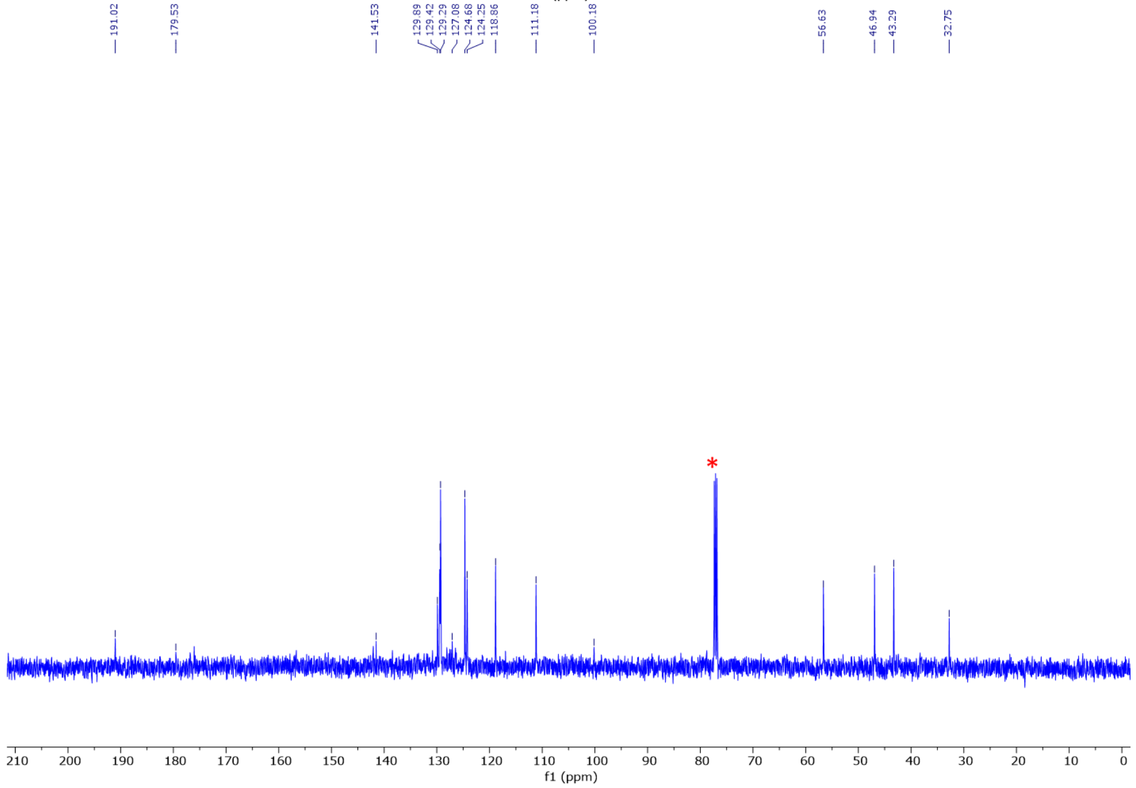

^{1}H NMR (500 MHz, $CDCl_3$), ^{13}C NMR (125 MHz, $CDCl_3$)

1-benzyl-5-chloro-3-(4-hydroxy-3-methyl-4-phenyl-2-thioxothiazolidin-5-yl)indolin-2-one (3f)

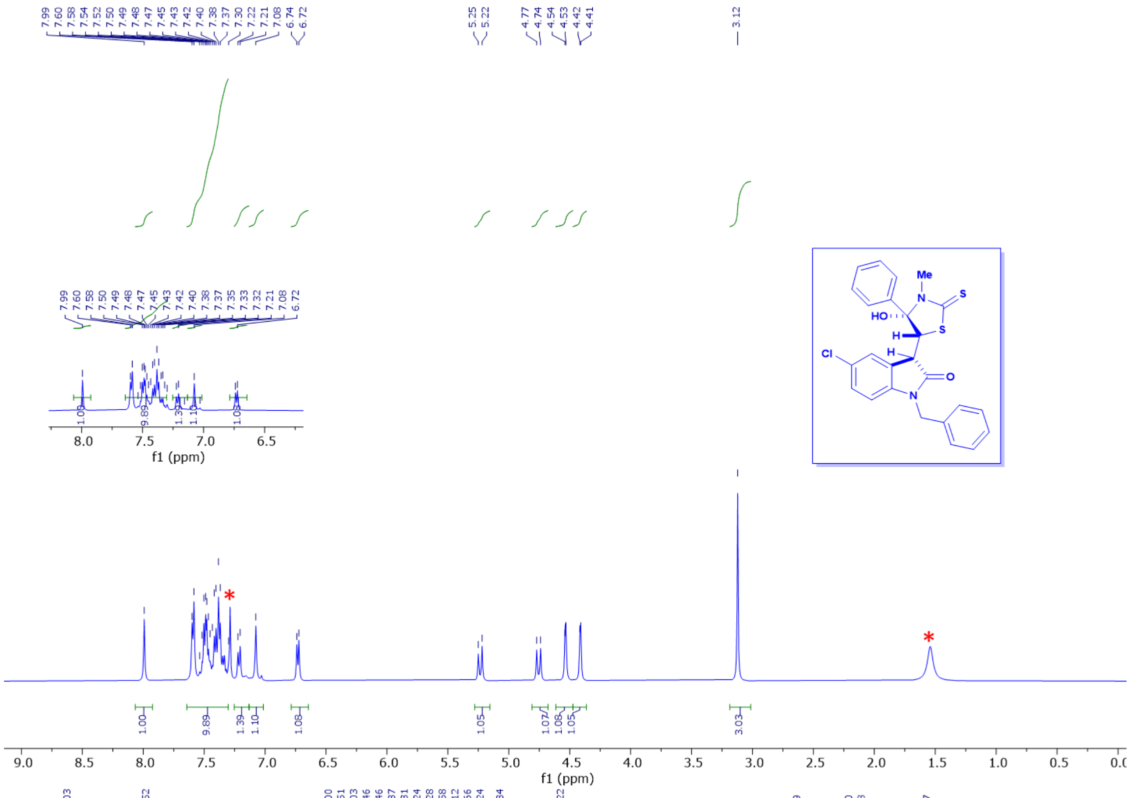

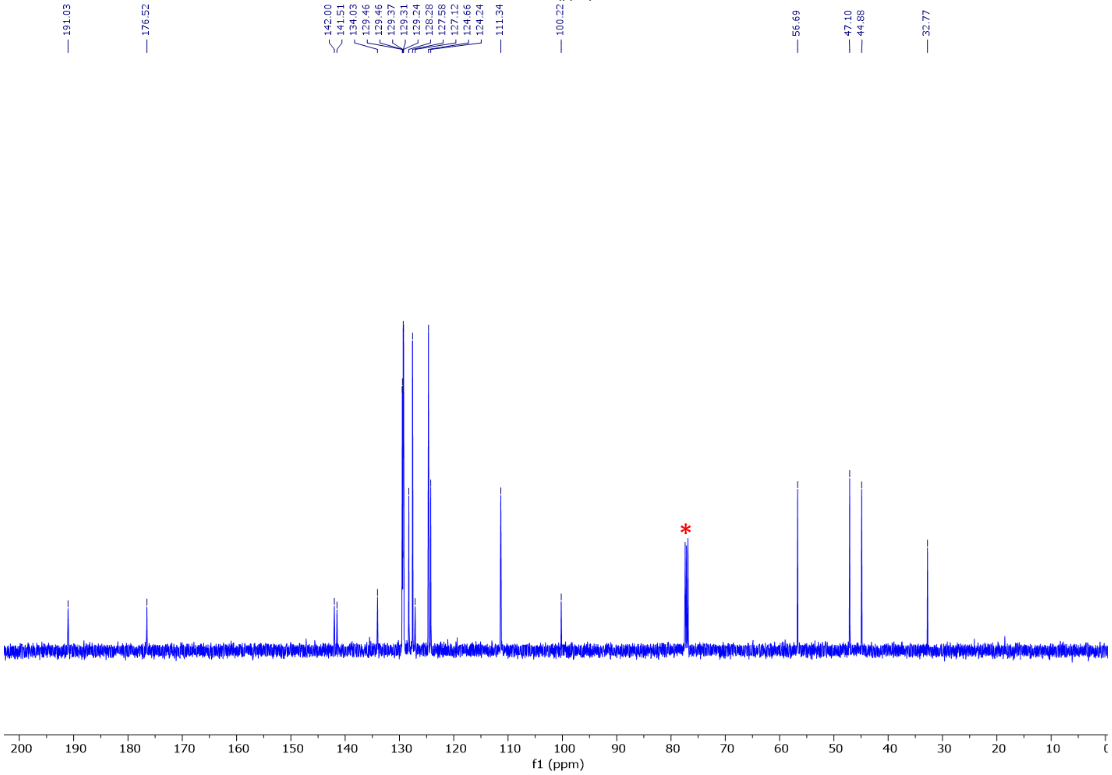

^{1}H NMR (500 MHz, $CDCl_3$), ^{13}C NMR (125 MHz, $CDCl_3$)

3-(4-hydroxy-3-methyl-4-phenyl-2-thioxothiazolidin-5-yl)-1-methylindolin-2-one (3g)

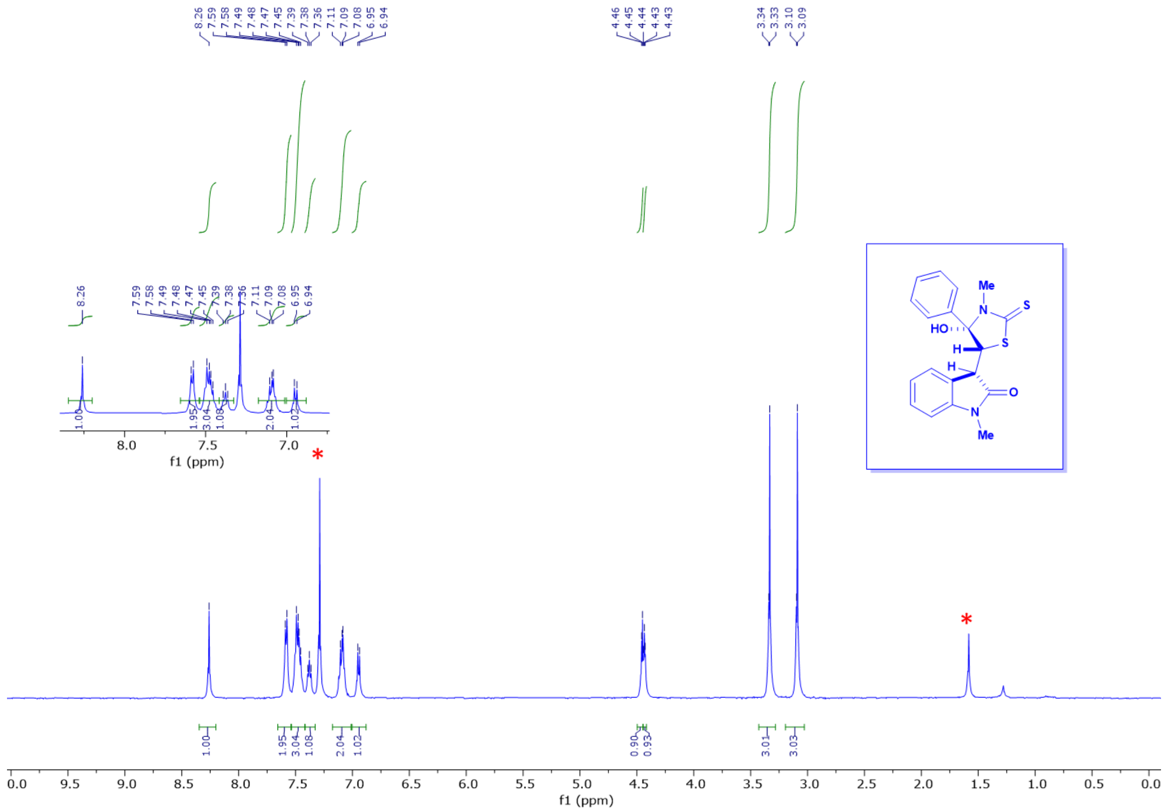

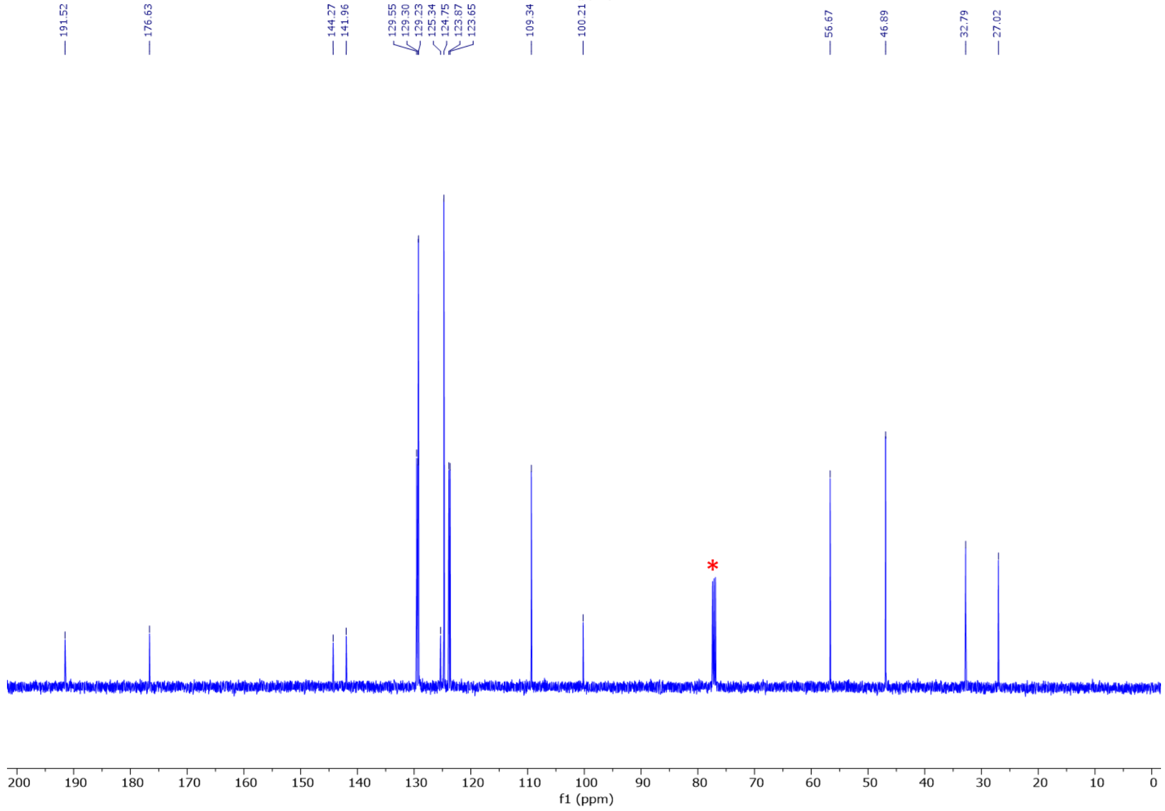

^{1}H NMR (500 MHz, $CDCl_3$), ^{13}C NMR (125 MHz, $CDCl_3$)

4-hydroxy-3-methyl-4-phenyl-2-thioxothiazolidin-5-yl)indolin-2-one (3h)

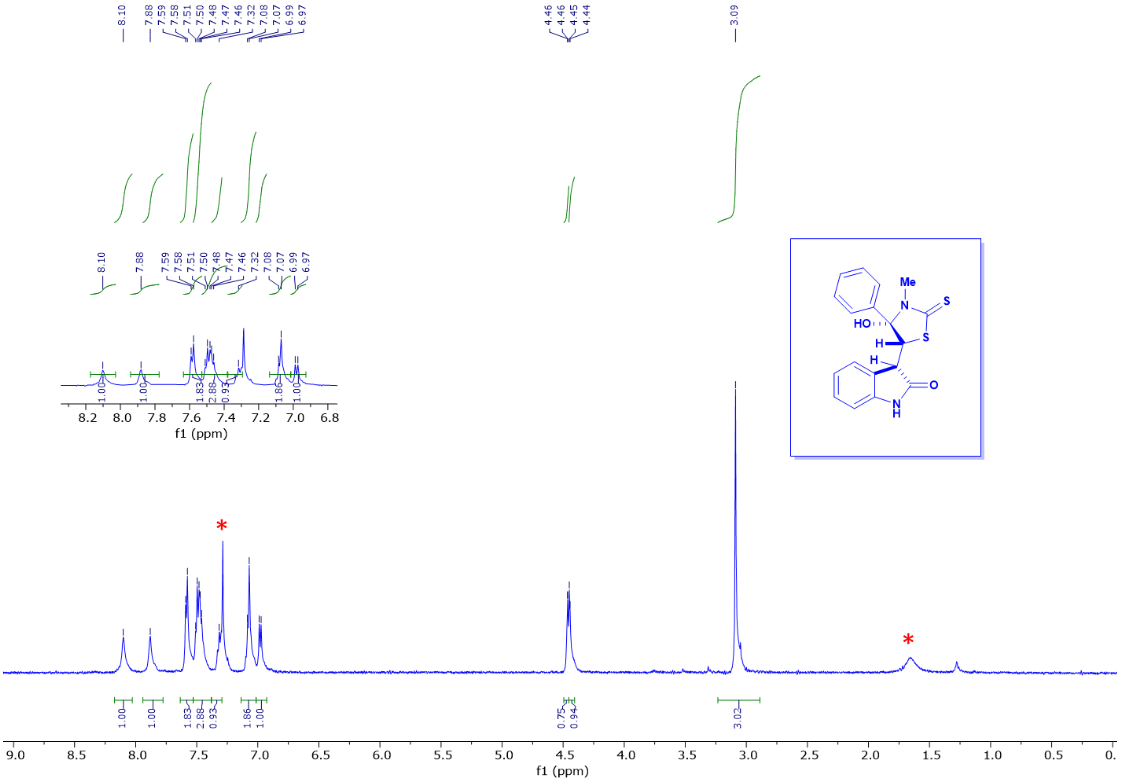

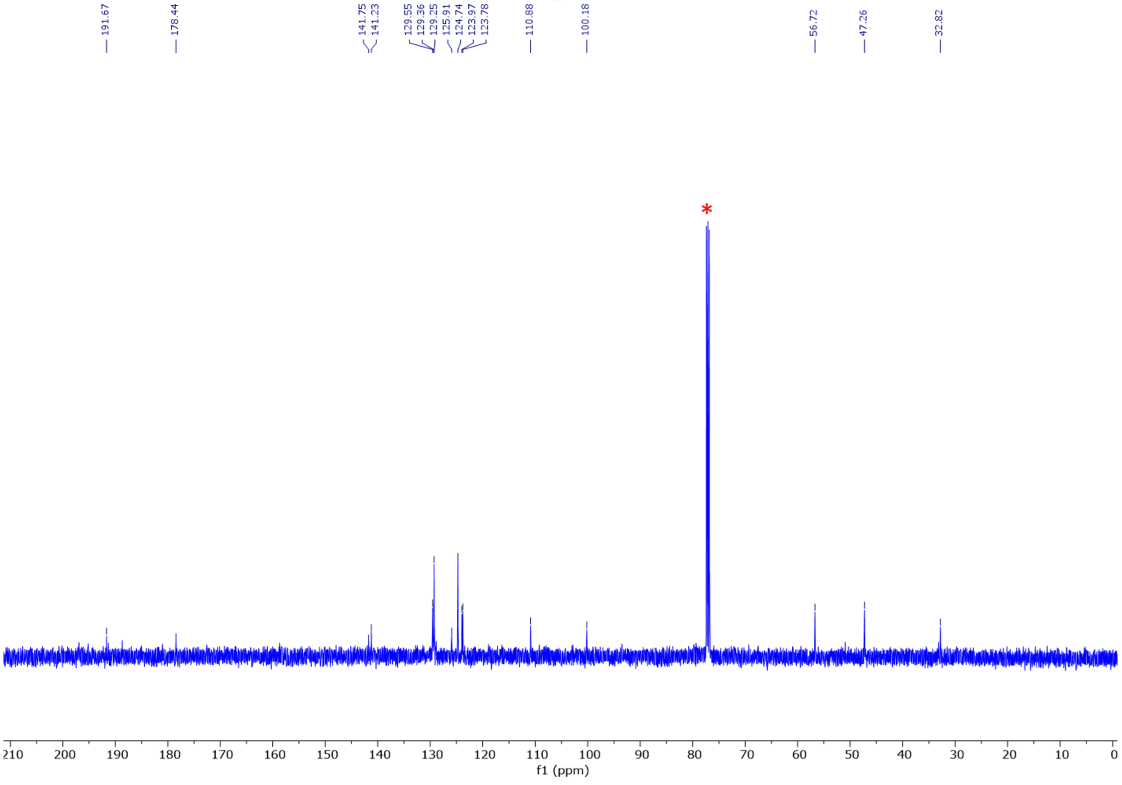

^{1}H NMR (500 MHz, $CDCl_3$), ^{13}C NMR (125 MHz, $CDCl_3$)

3-(4-hydroxy-3-methyl-4-phenyl-2-thioxothiazolidin-5-yl)-1-isopropylindolin-2-one (3i)

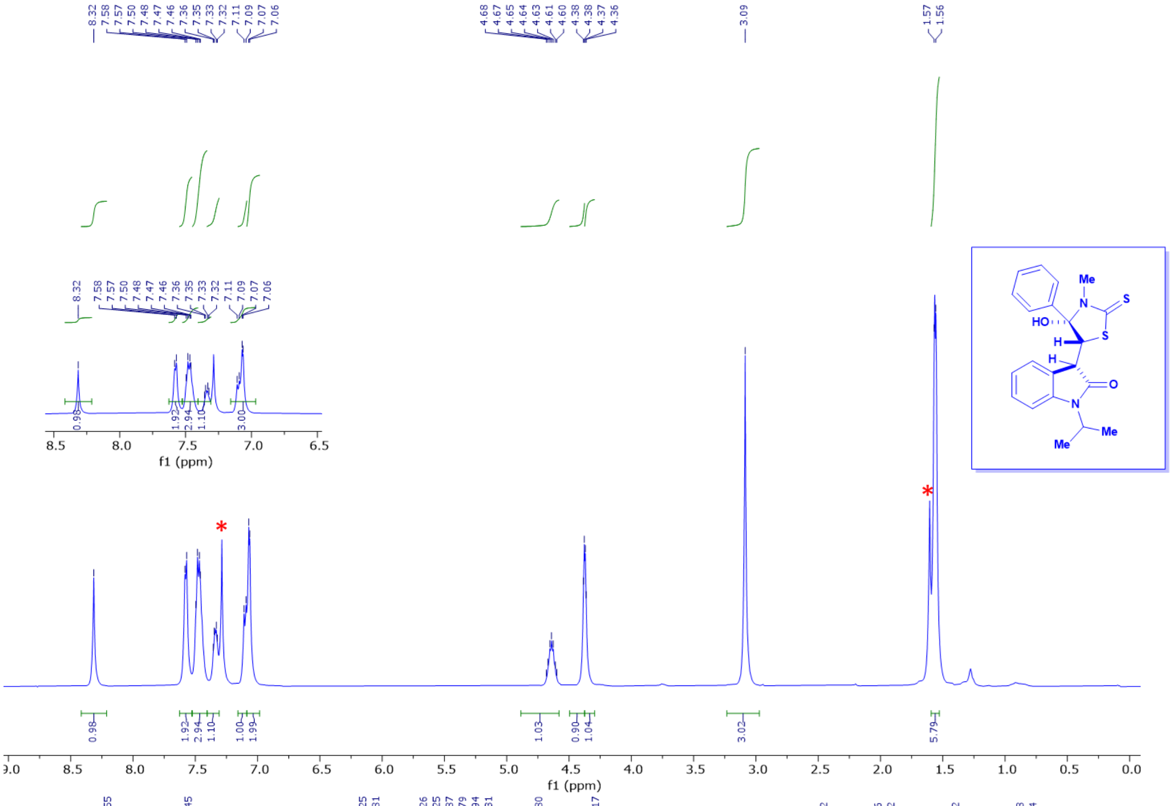

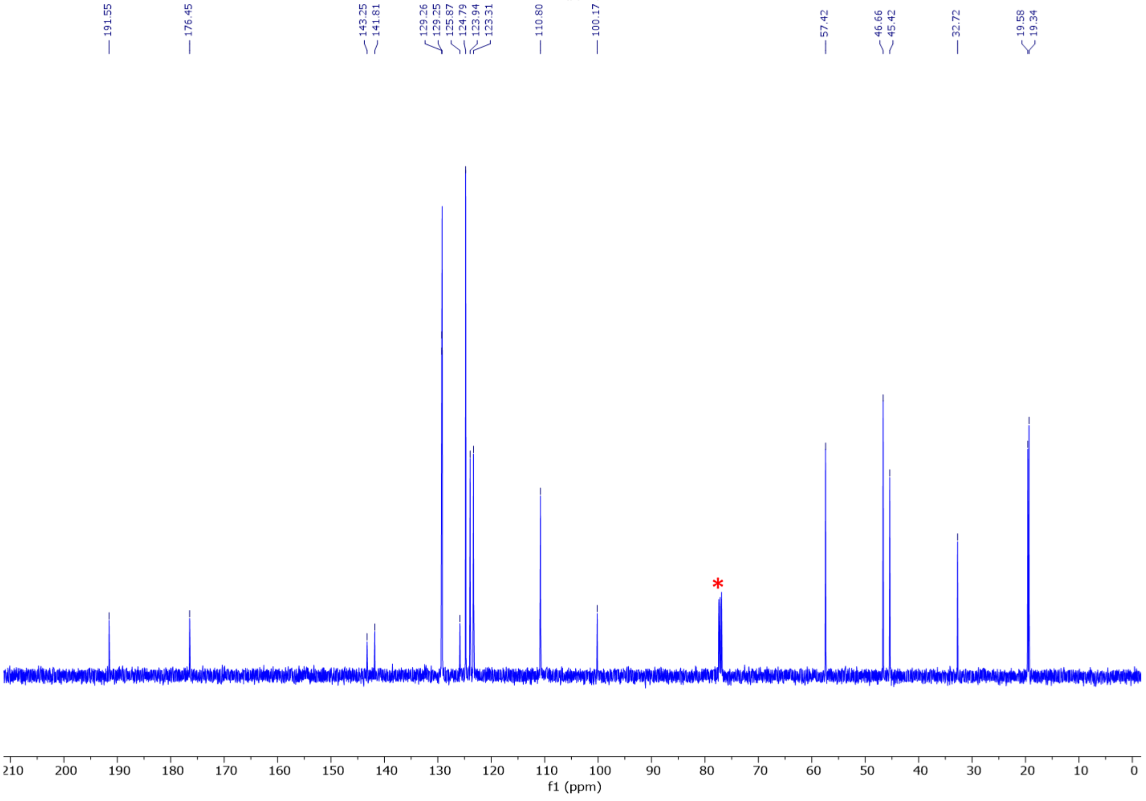

^{1}H NMR (500 MHz, $CDCl_3$), ^{13}C NMR (125 MHz, $CDCl_3$)

ethyl 2-(3-(4-hydroxy-3-methyl-4-phenyl-2-thioxothiazolidin-5-yl)-2-oxoindolin-1-yl)acetate (3j)

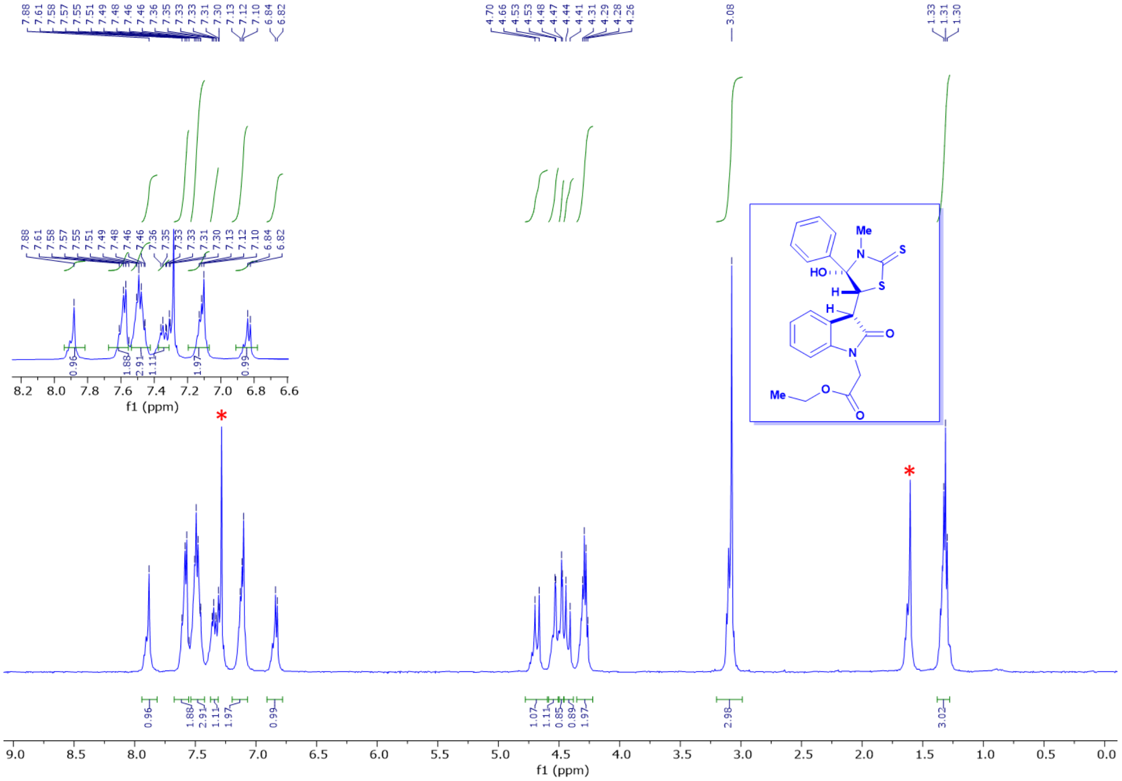

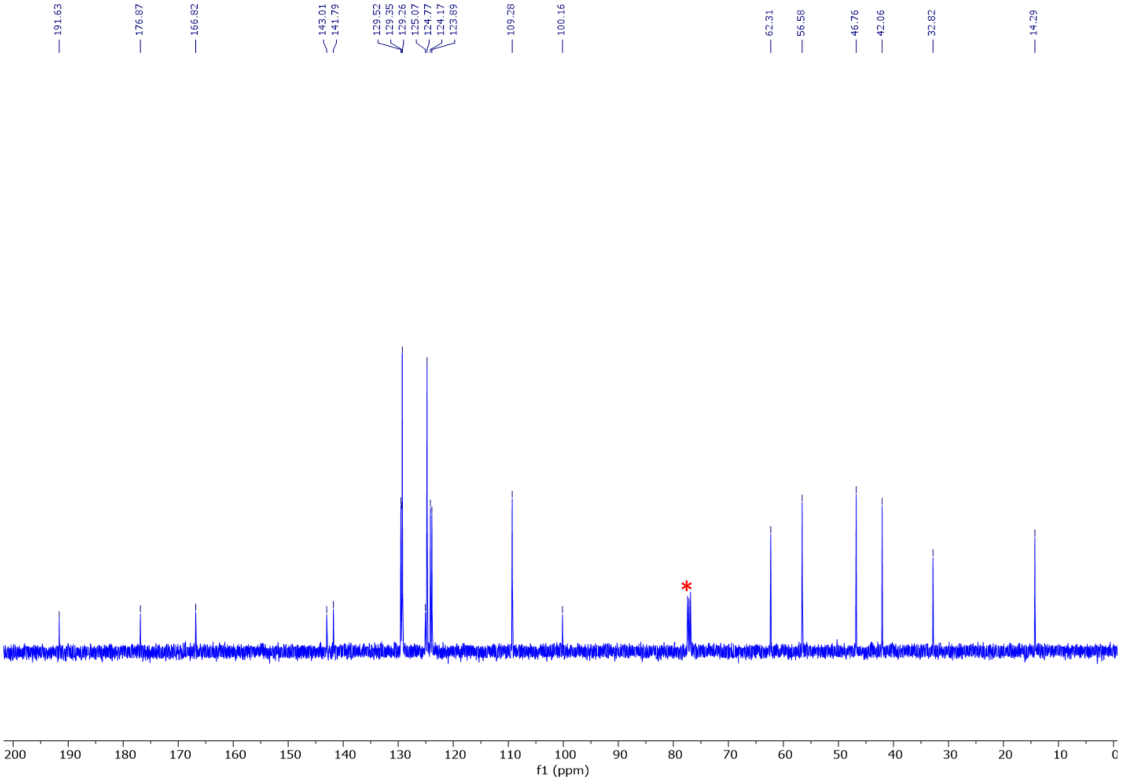

^{1}H NMR (500 MHz, $CDCl_3$), ^{13}C NMR (125 MHz, $CDCl_3$)

3-(4-hydroxy-3-methyl-4-phenyl-2-thioxothiazolidin-5-yl)-1-(naphthalen-1-ylmethyl)indolin-2-one (3k)

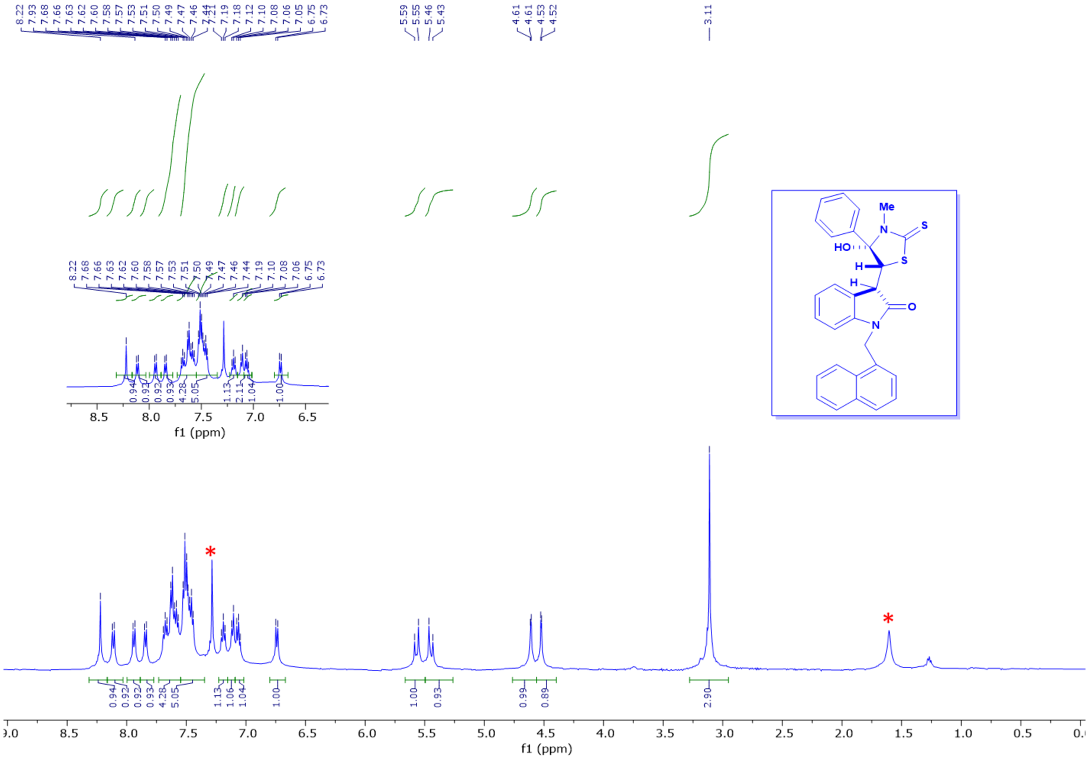

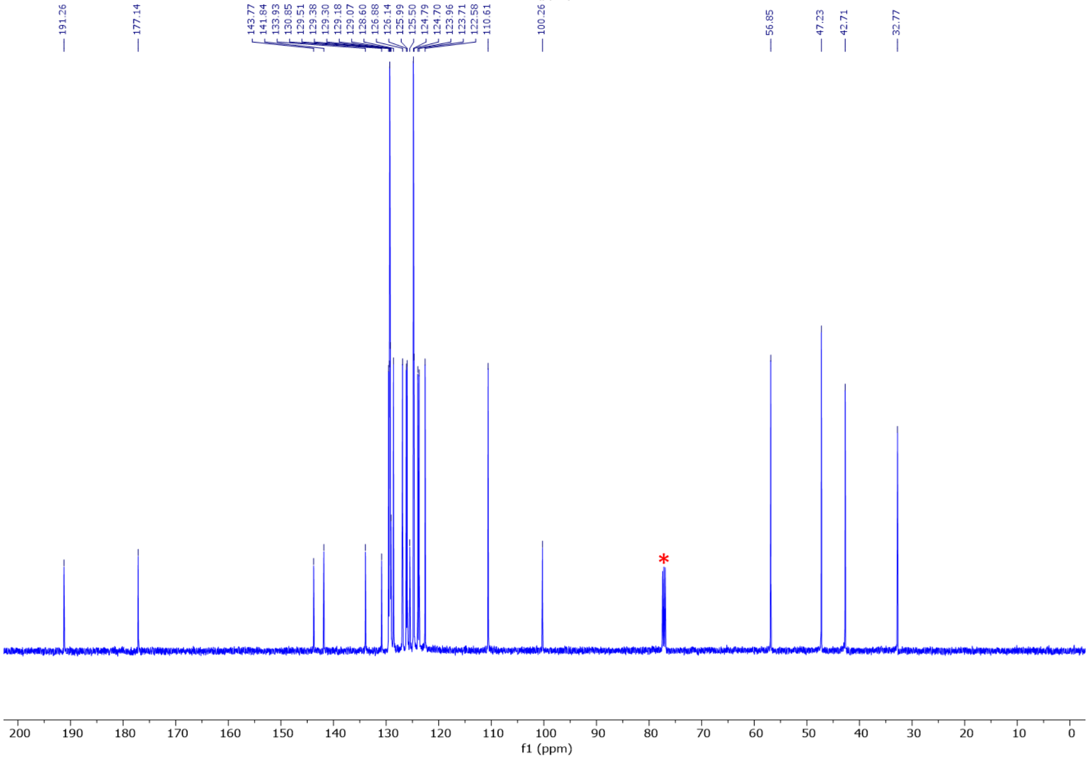

^{1}H NMR (500 MHz, $CDCl_3$), ^{13}C NMR (125 MHz, $CDCl_3$)

1-benzyl-3-(3-ethyl-4-hydroxy-4-phenyl-2-thioxothiazolidin-5-yl) indolin-2-one (3l)

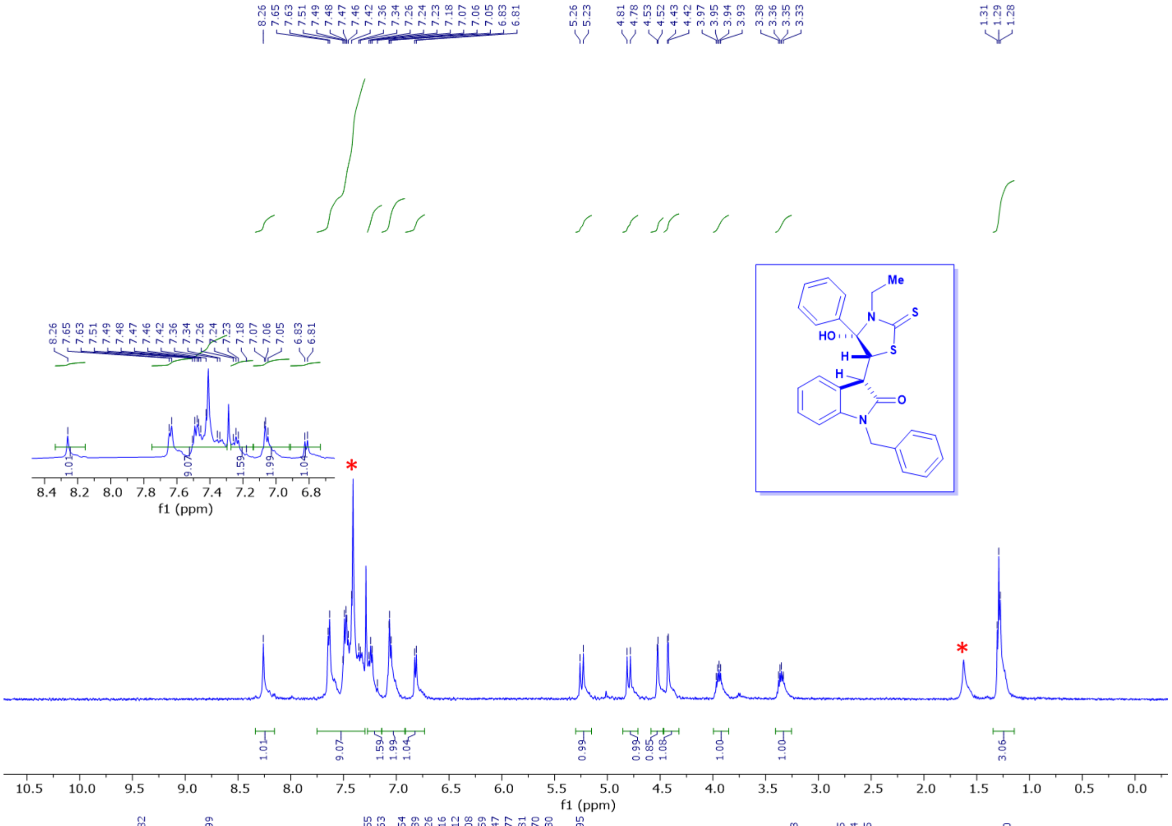

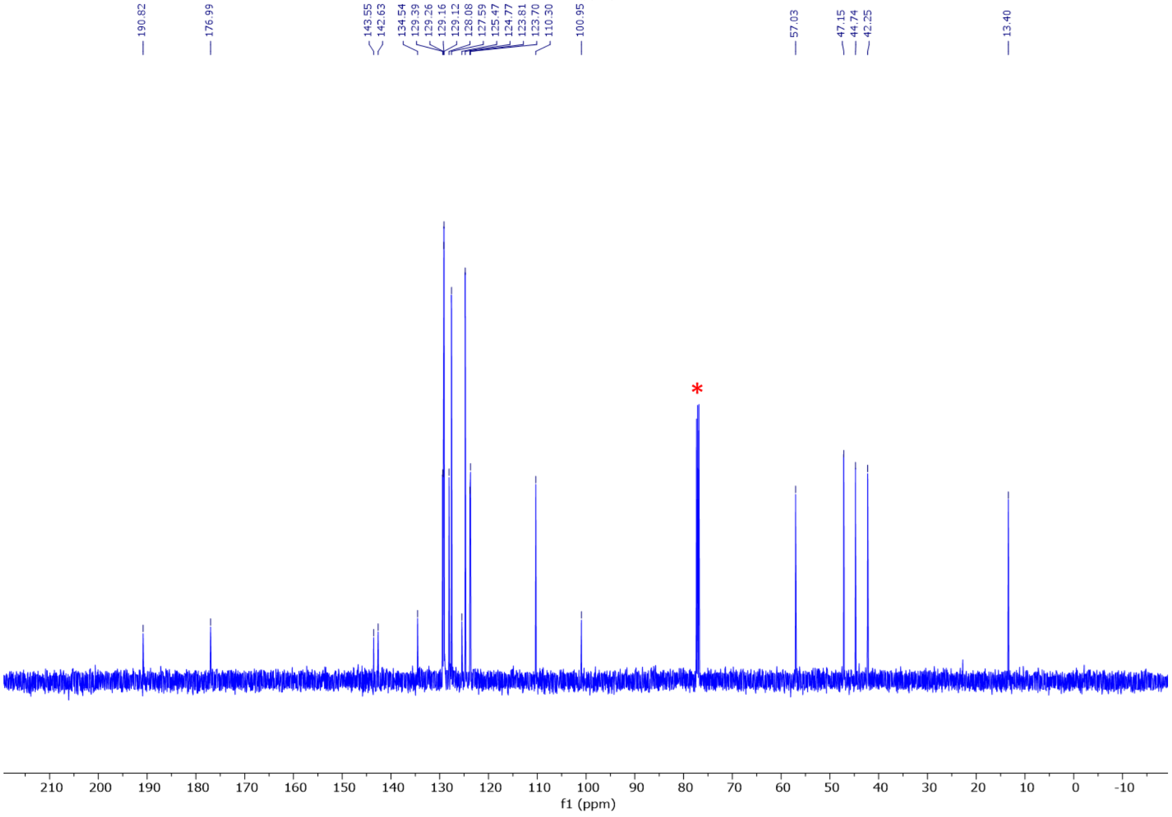

^{1}H NMR (500 MHz, $CDCl_3$), ^{13}C NMR (125 MHz, $CDCl_3$)

1-allyl-3-(3-ethyl-4-hydroxy-4-phenyl-2-thioxothiazolidin-5-yl)indolin-2-one (3m)

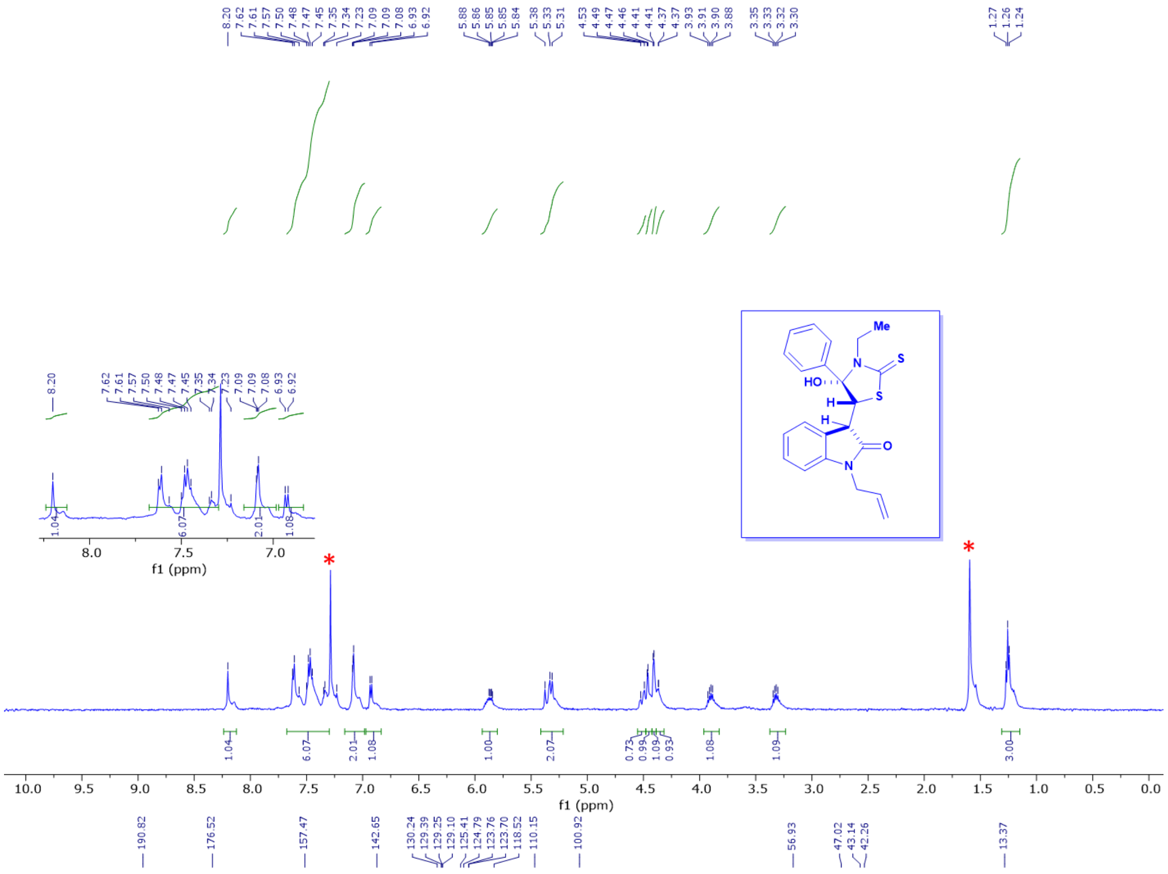

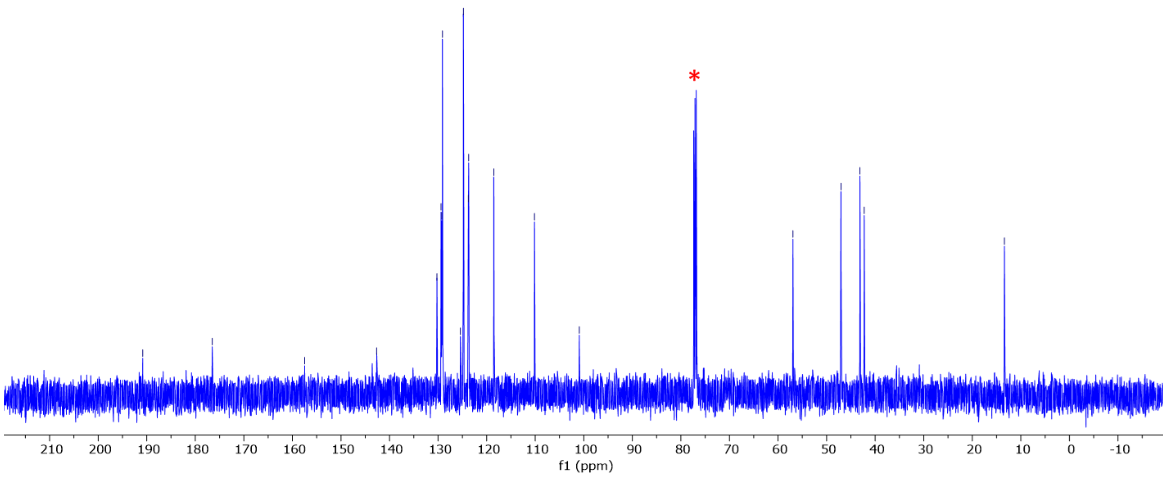

^{1}H NMR (500 MHz, $CDCl_3$), ^{13}C NMR (125 MHz, $CDCl_3$)

3-(3-ethyl-4-hydroxy-4-phenyl-2-thioxothiazolidin-5-yl)-1-propylindolin-2-one (3n)

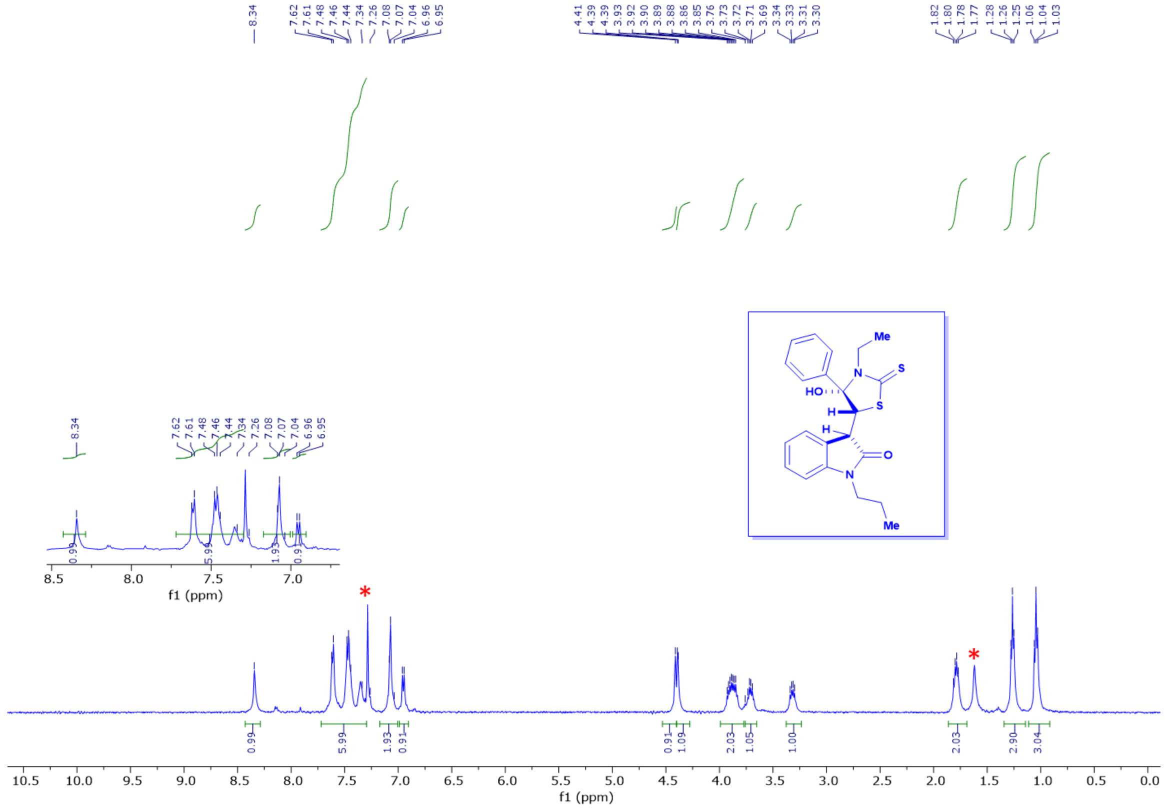

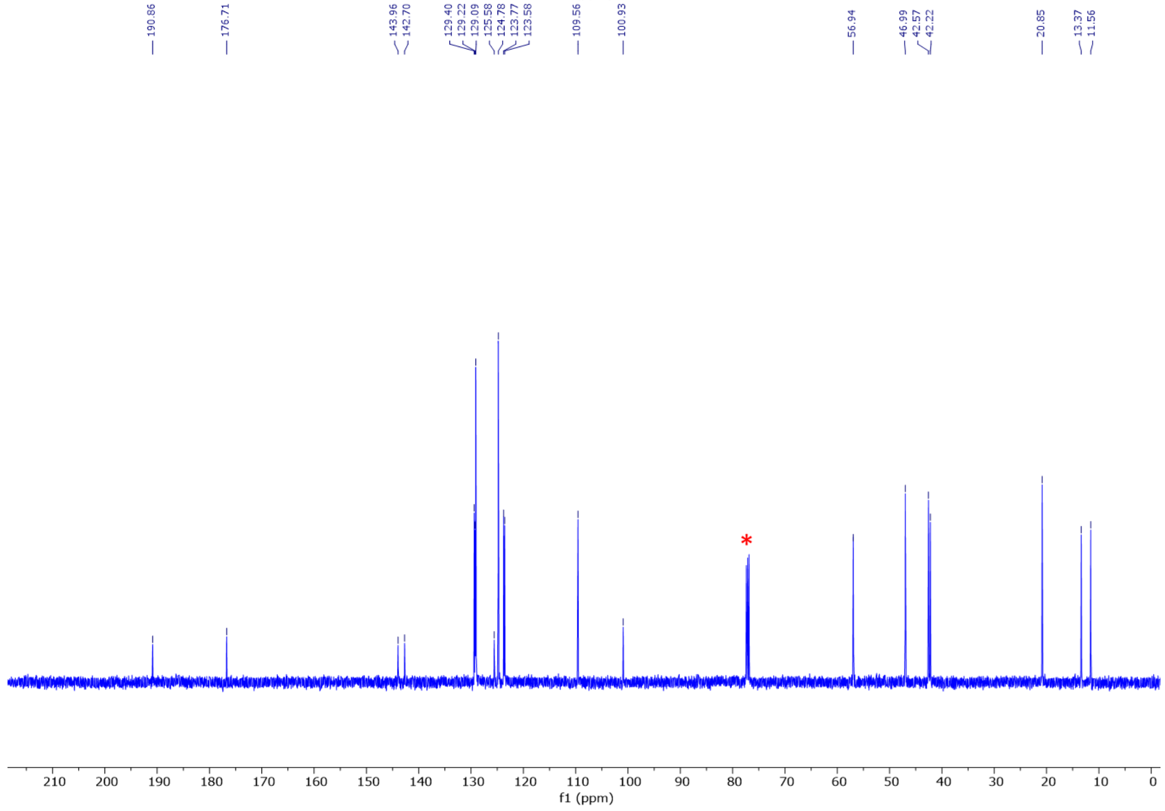

^{1}H NMR (500 MHz, $CDCl_3$), ^{13}C NMR (125 MHz, $CDCl_3$)

5-chloro-3-(3-ethyl-4-hydroxy-4-phenyl-2-thioxothiazolidin-5-yl)-1-propylindolin-2-one (3o)

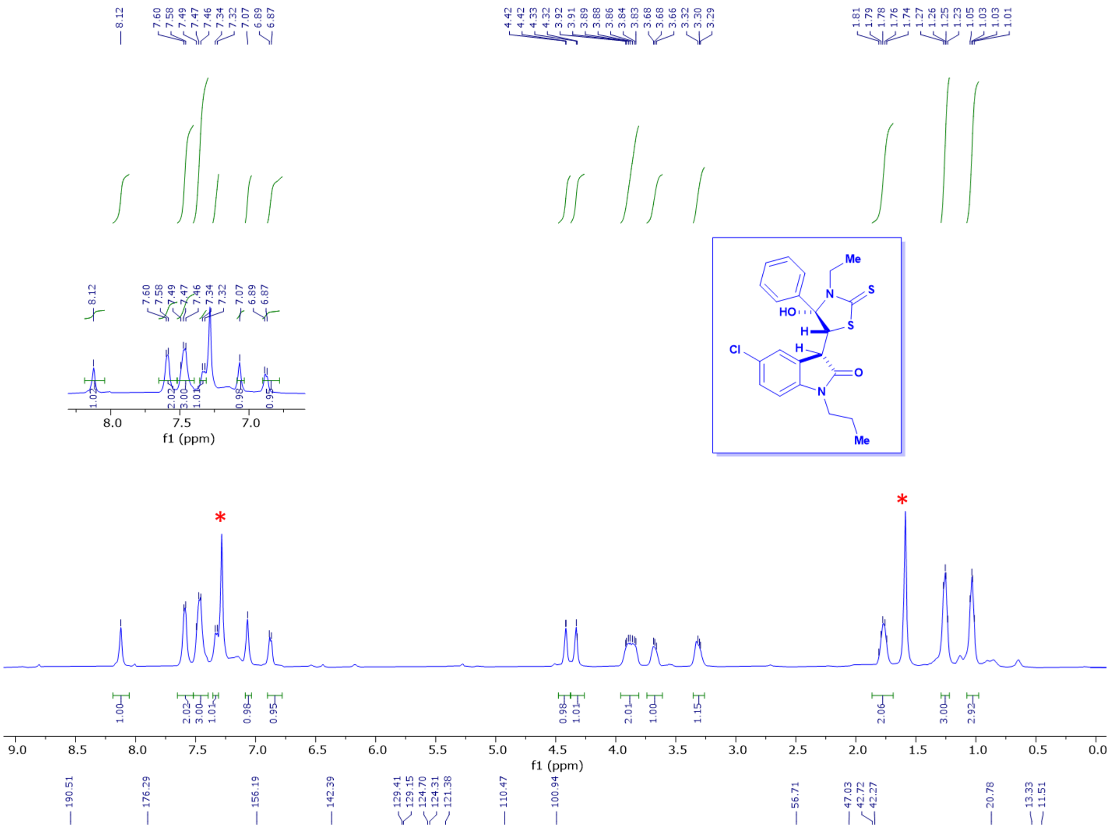

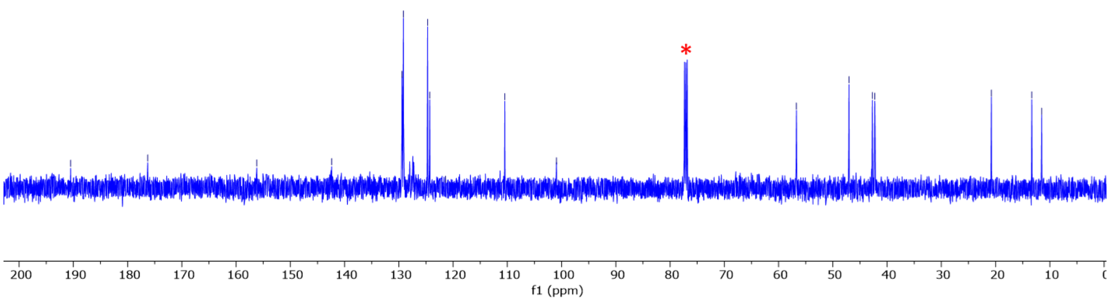

^{1}H NMR (500 MHz, $CDCl_3$), ^{13}C NMR (125 MHz, $CDCl_3$)

ethyl 2-(3-(3-ethyl-4-hydroxy-4-phenyl-2-thioxothiazolidin-5-yl)-2-oxoindolin-1-yl)acetate (3p)

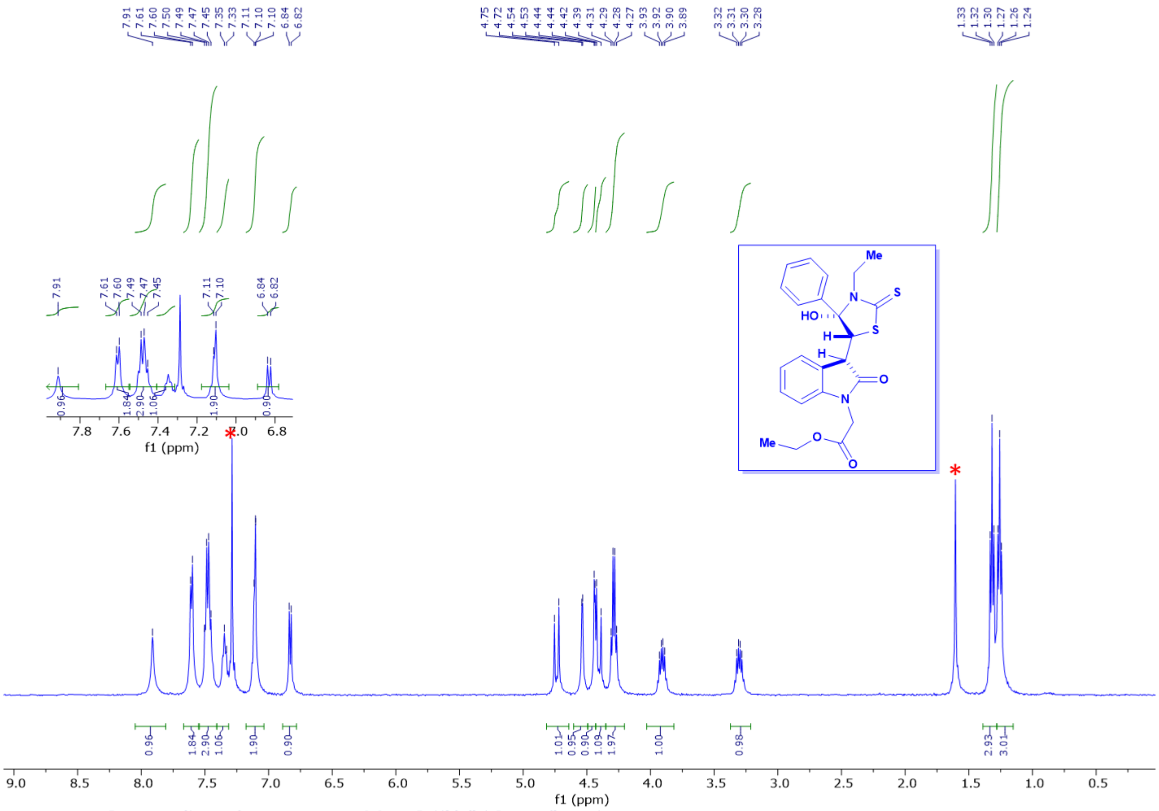

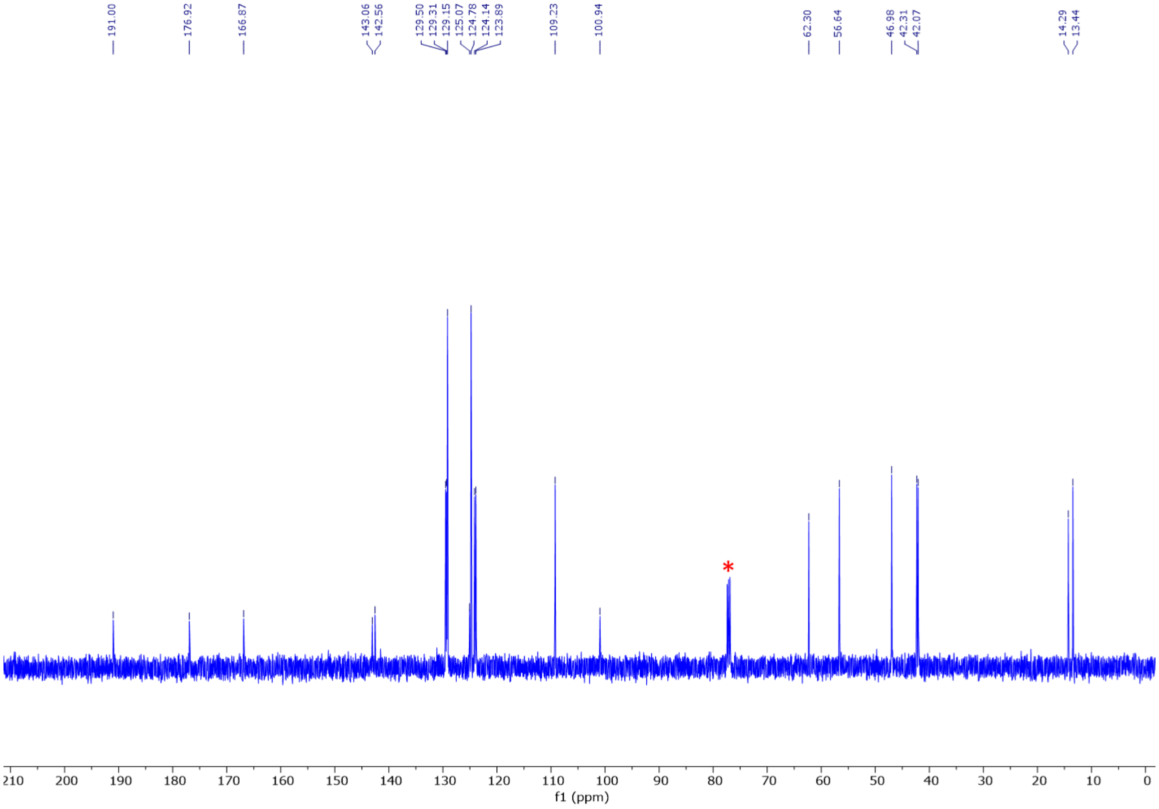

^{1}H NMR (500 MHz, $CDCl_3$), ^{13}C NMR (125 MHz, $CDCl_3$)

1-benzyl-5-chloro-3-(3-ethyl-4-hydroxy-4-phenyl-2-thioxothiazolidin-5-yl)indolin-2-one (3q)

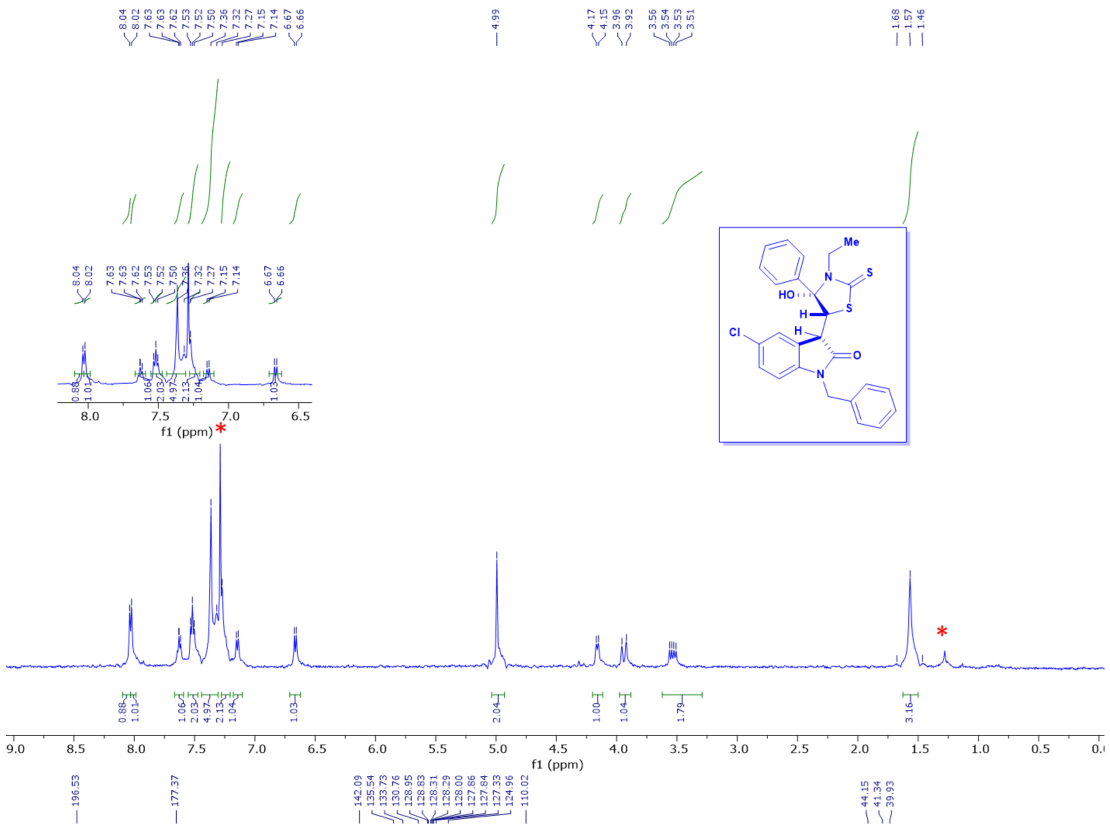

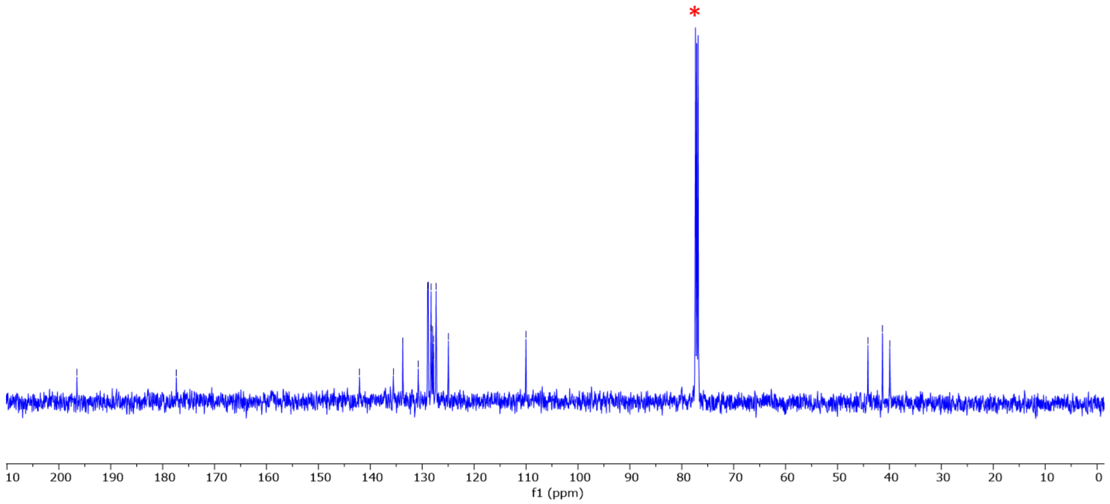

^{1}H NMR (500 MHz, $CDCl_3$), ^{13}C NMR (125 MHz, $CDCl_3$)

3-(3-ethyl-4-hydroxy-4-phenyl-2-thioxothiazolidin-5-yl)-1-methylindolin-2-one (3r)

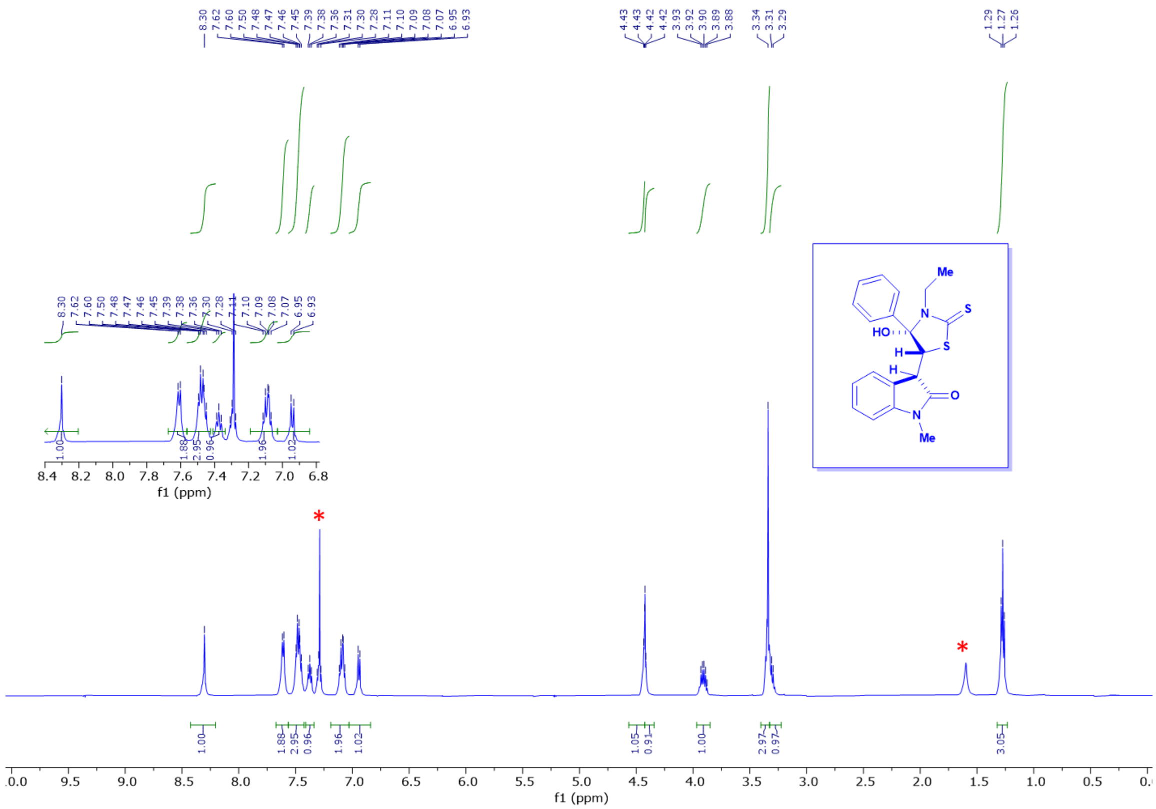

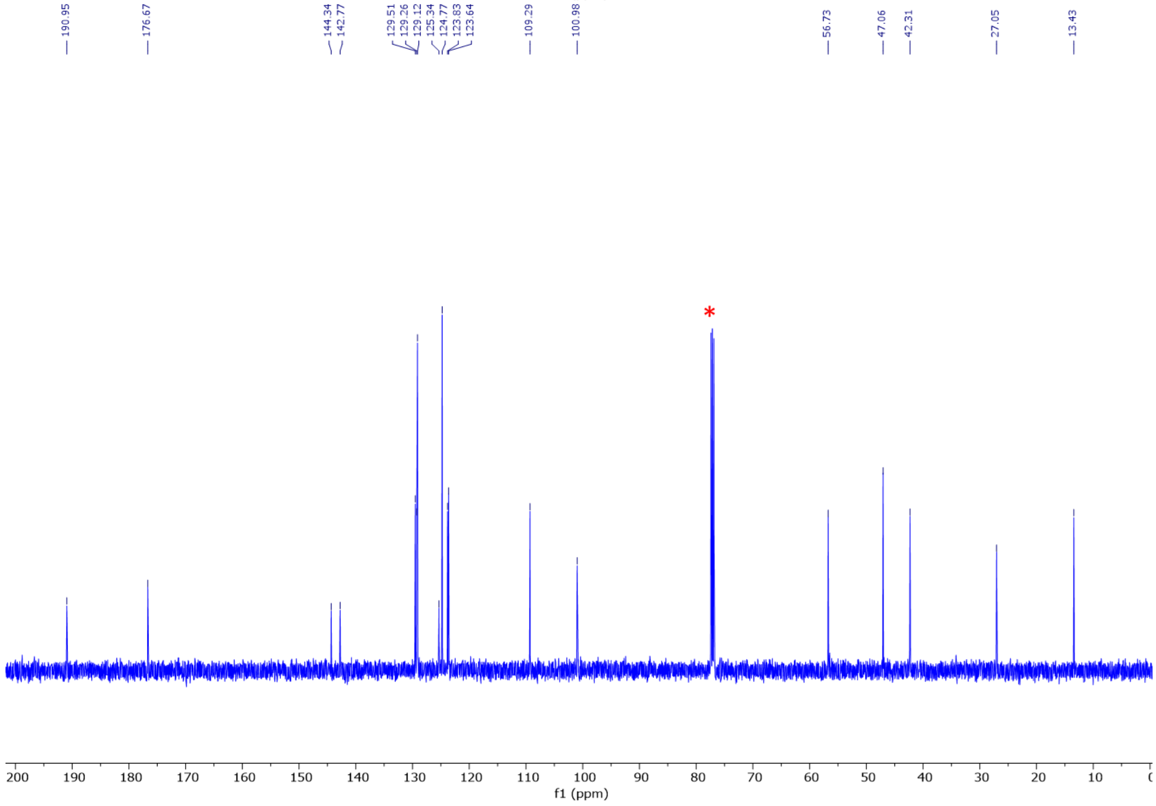

^{1}H NMR (500 MHz, $CDCl_3$), ^{13}C NMR (125 MHz, $CDCl_3$)

3-(-3-benzyl-4-hydroxy-4-phenyl-2-thioxothiazolidin-5-yl)-1-propylindolin-2-one (3s)

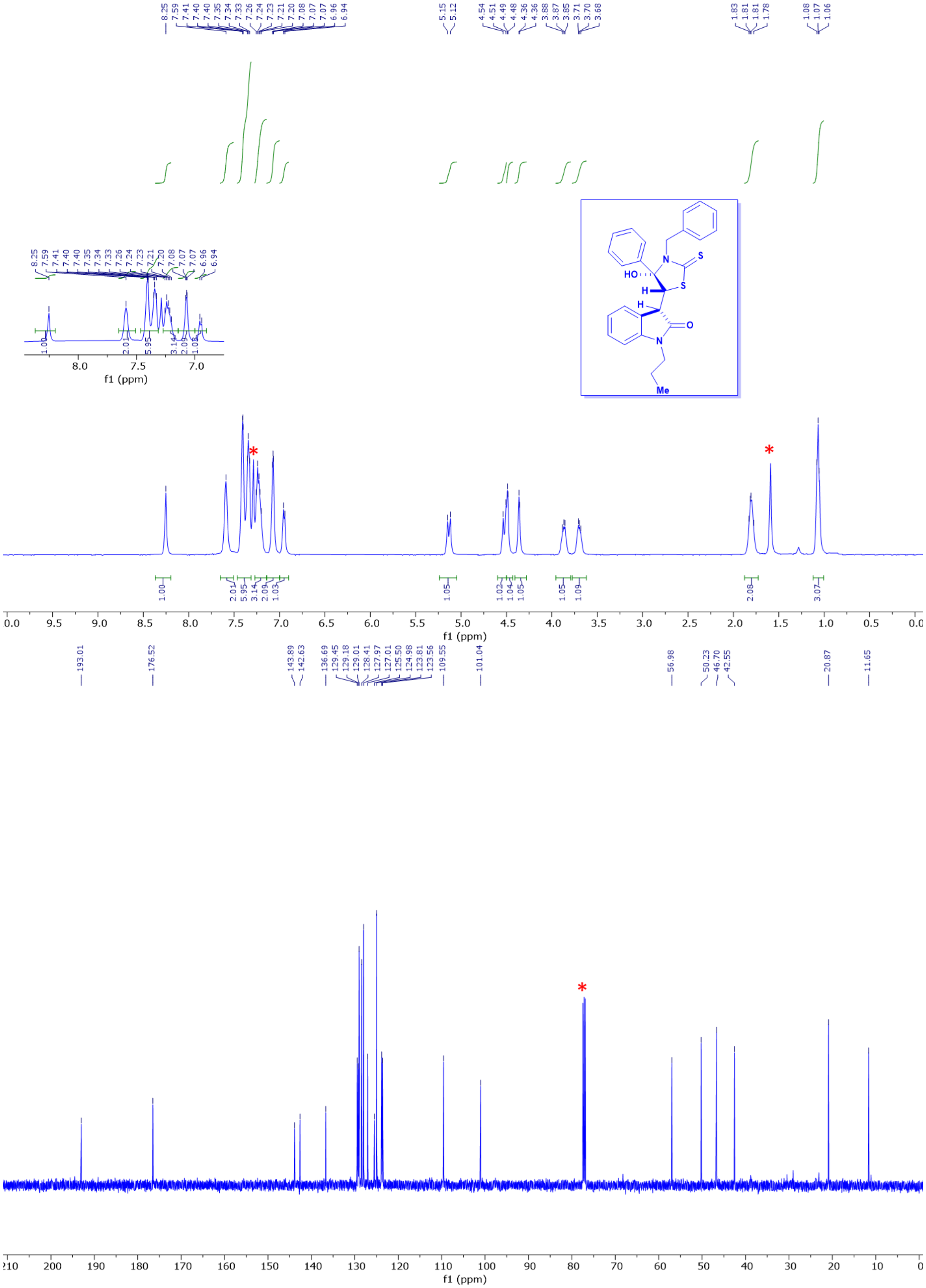

^{1}H NMR (500 MHz, $CDCl_3$), ^{13}C NMR (125 MHz, $CDCl_3$)

3-(3-benzyl-4-hydroxy-4-phenyl-2-thioxothiazolidin-5-yl)-1-methylindolin-2-one (3t)

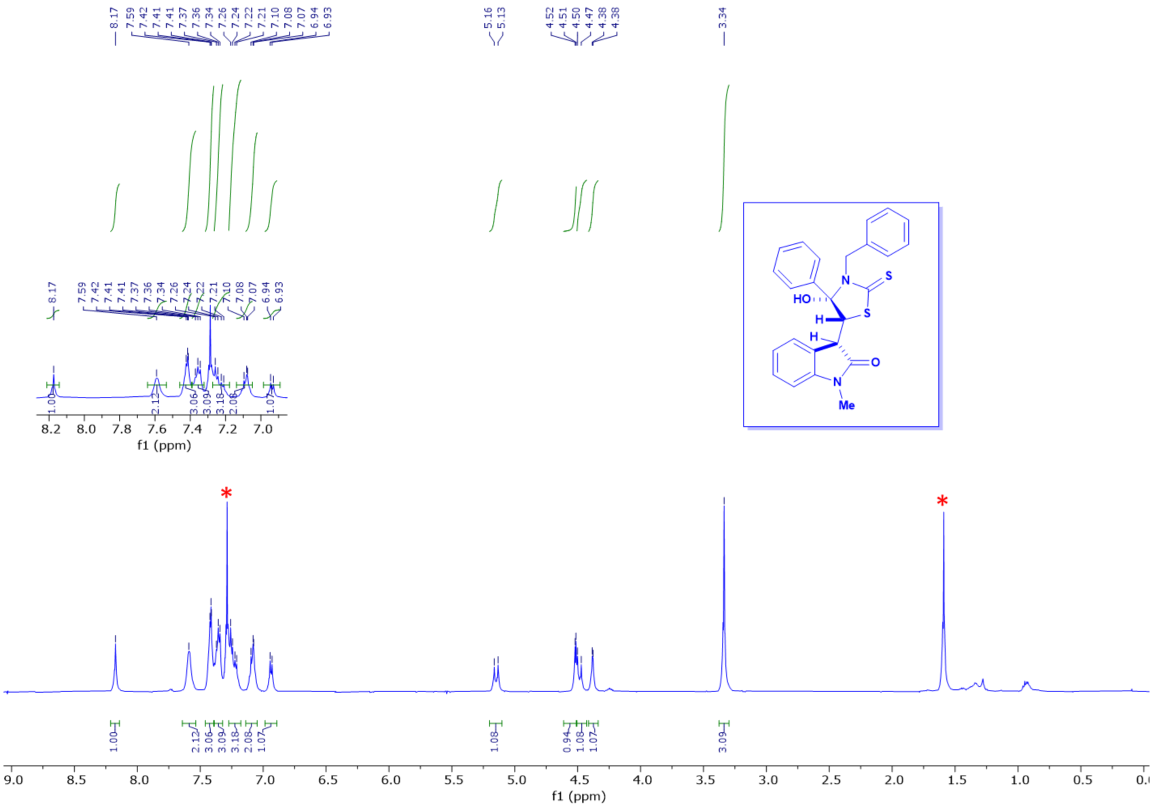

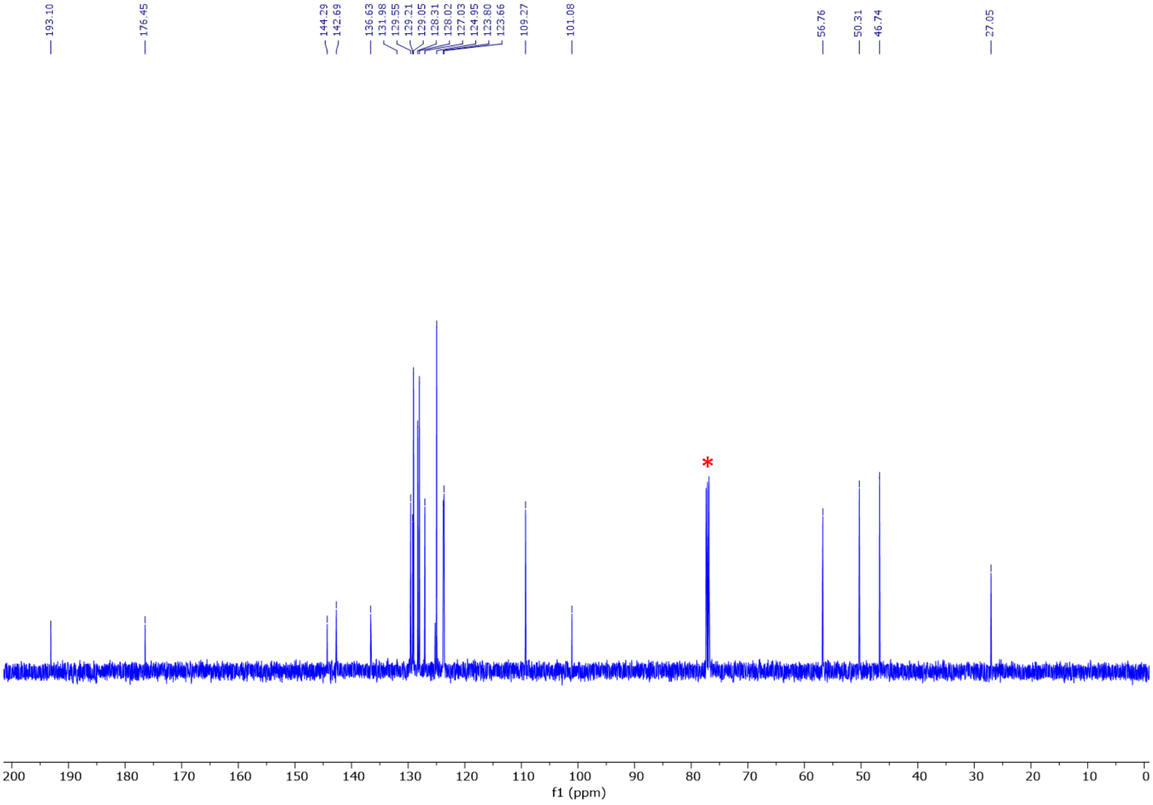

^{1}H NMR (500 MHz, $CDCl_3$), ^{13}C NMR (125 MHz, $CDCl_3$)

1-allyl-3-(3-benzyl-4-hydroxy-4-phenyl-2-thioxothiazolidin-5-yl)indolin-2-one (3u)

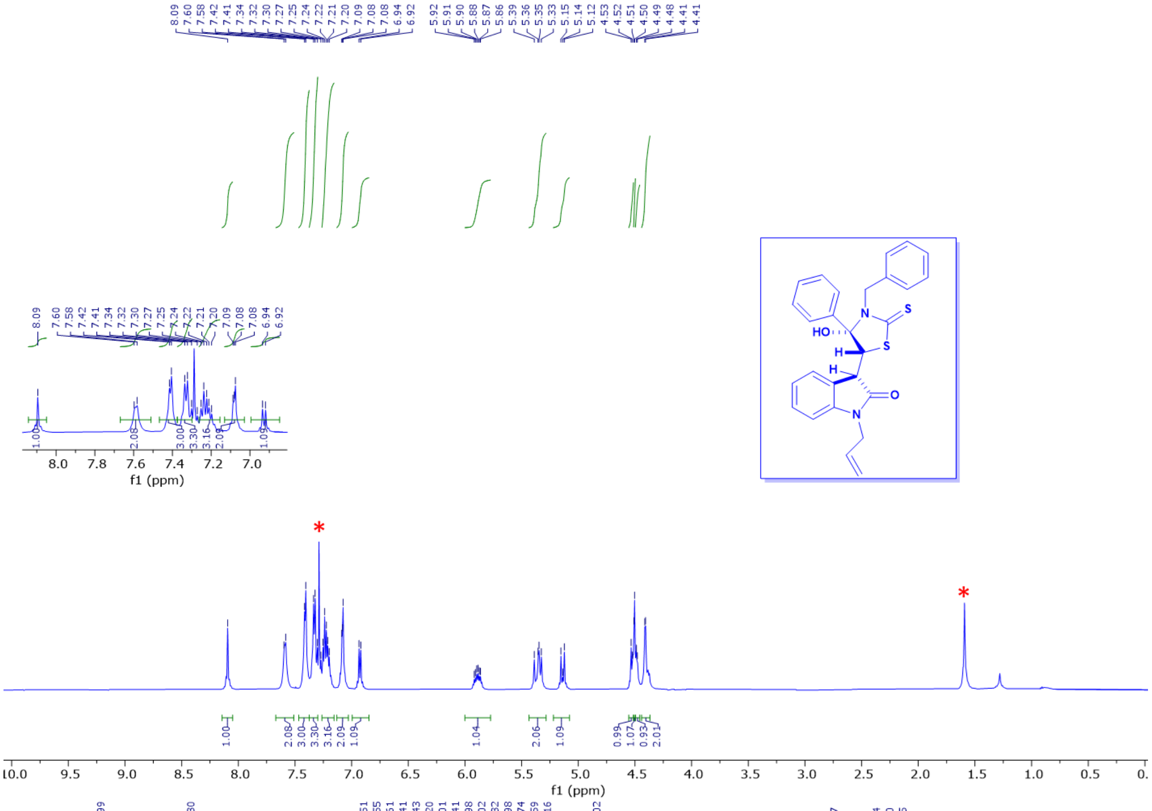

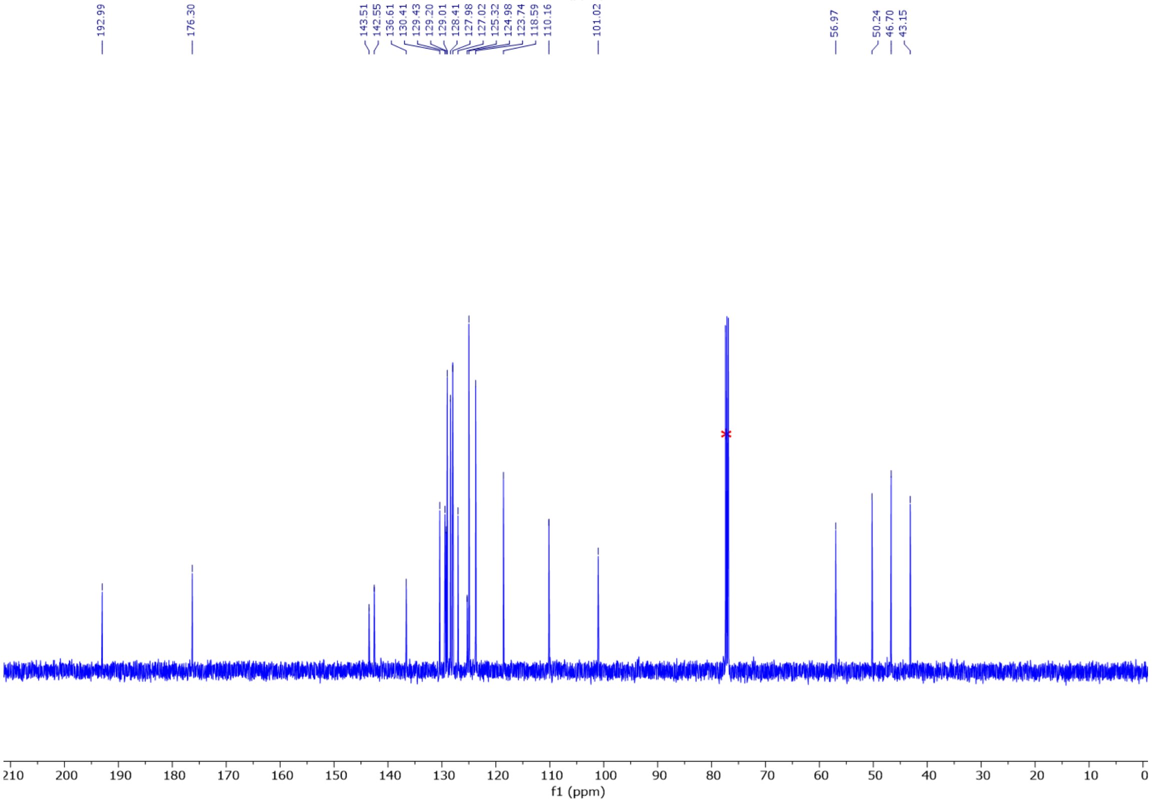

^{1}H NMR (500 MHz, $CDCl_3$), ^{13}C NMR (125 MHz, $CDCl_3$)

1-benzyl-3-(3-benzyl-4-hydroxy-4-phenyl-2-thioxothiazolidin-5-yl)indolin-2-one (3v)

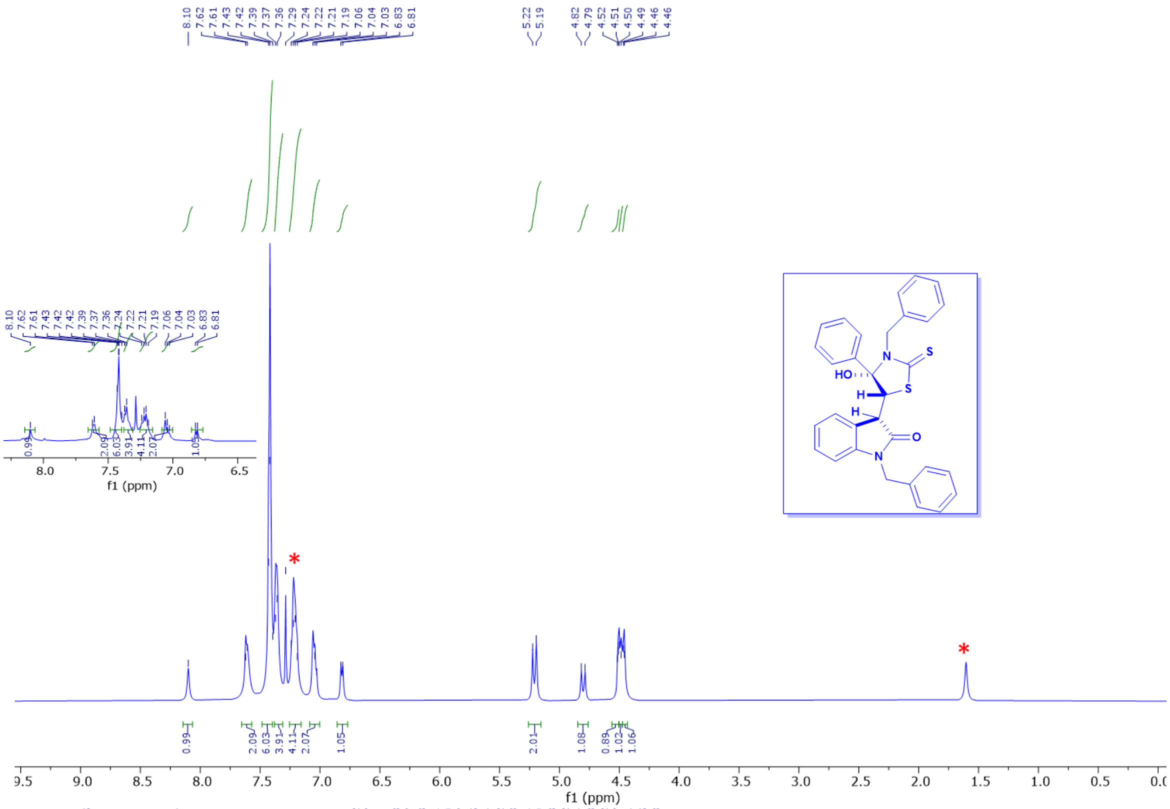

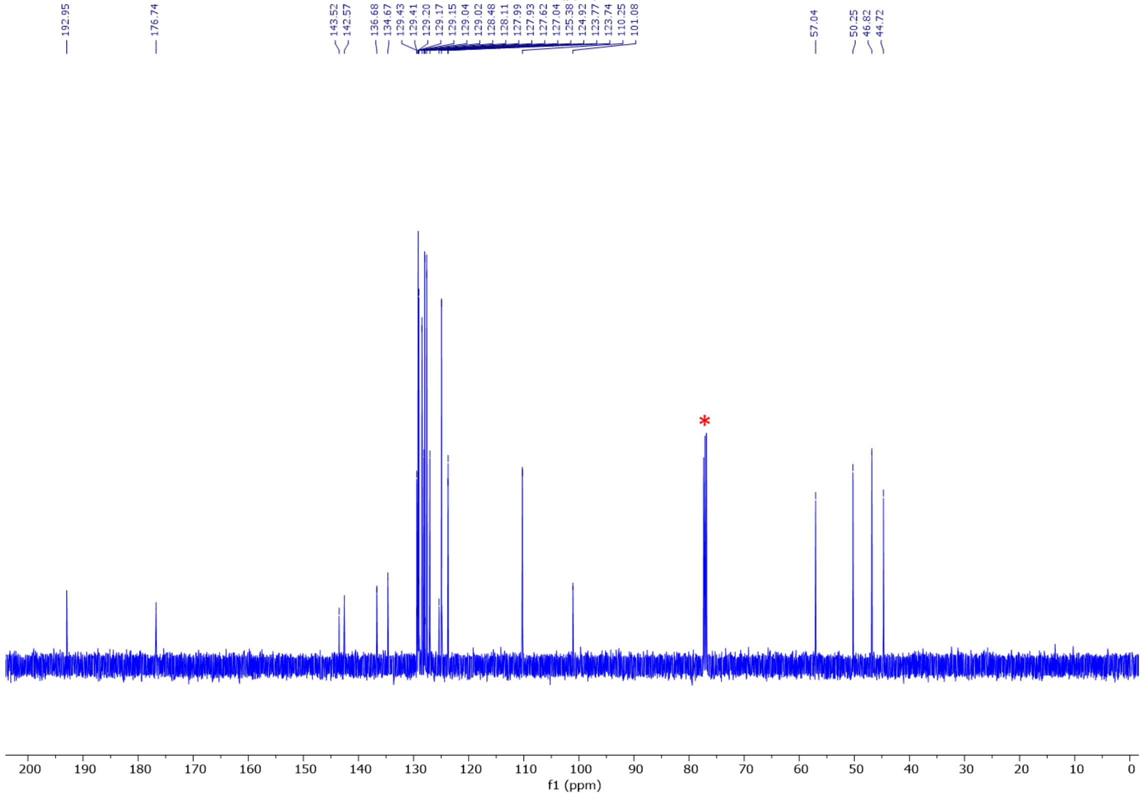

^{1}H NMR (500 MHz, $CDCl_3$), ^{13}C NMR (125 MHz, $CDCl_3$)

3-(3-benzyl-4-hydroxy-4-phenyl-2-thioxothiazolidin-5-yl)-1-(naphthalen-1-ylmethyl)indolin-2-one (3w)

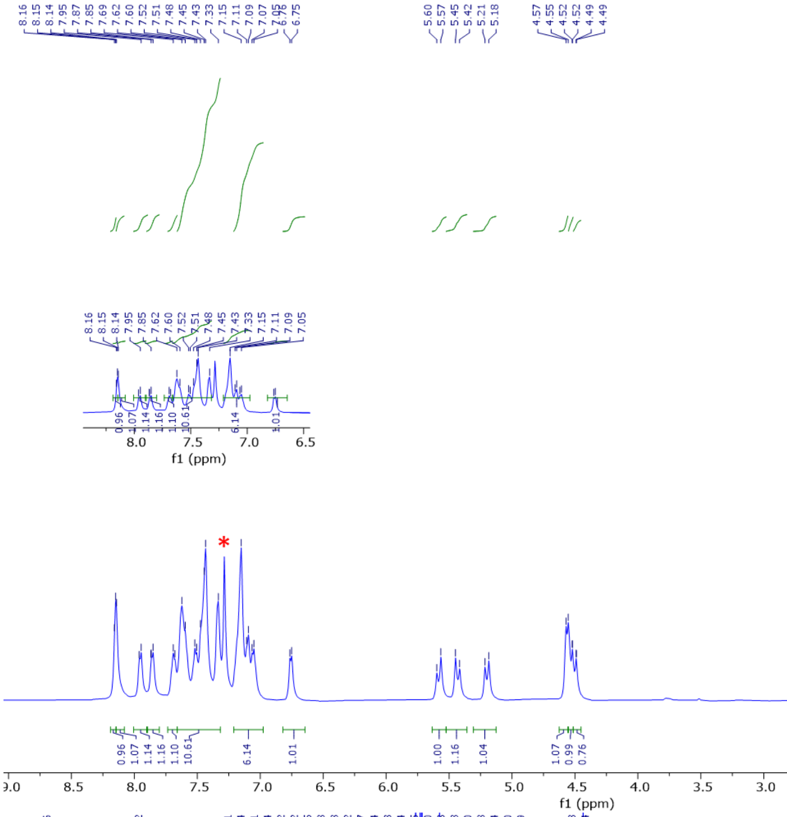

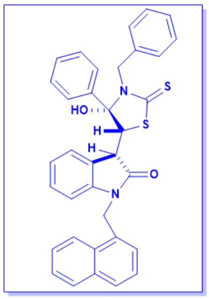

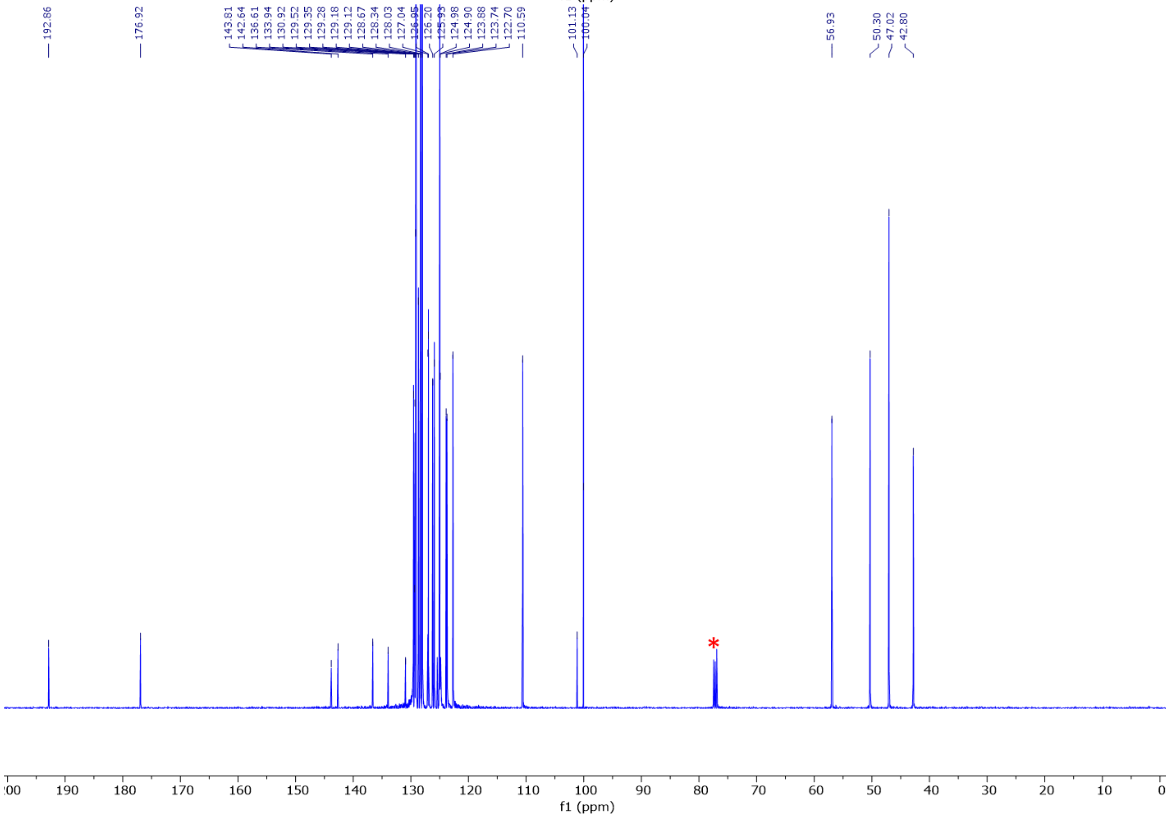

^{1}H NMR (500 MHz, $CDCl_3$), ^{13}C NMR (125 MHz, $CDCl_3$)

4-hydroxy-4-phenyl-3-(1-phenylethyl)-2-thioxothiazolidin-5-yl)-1-methylindolin-2-one (3x)

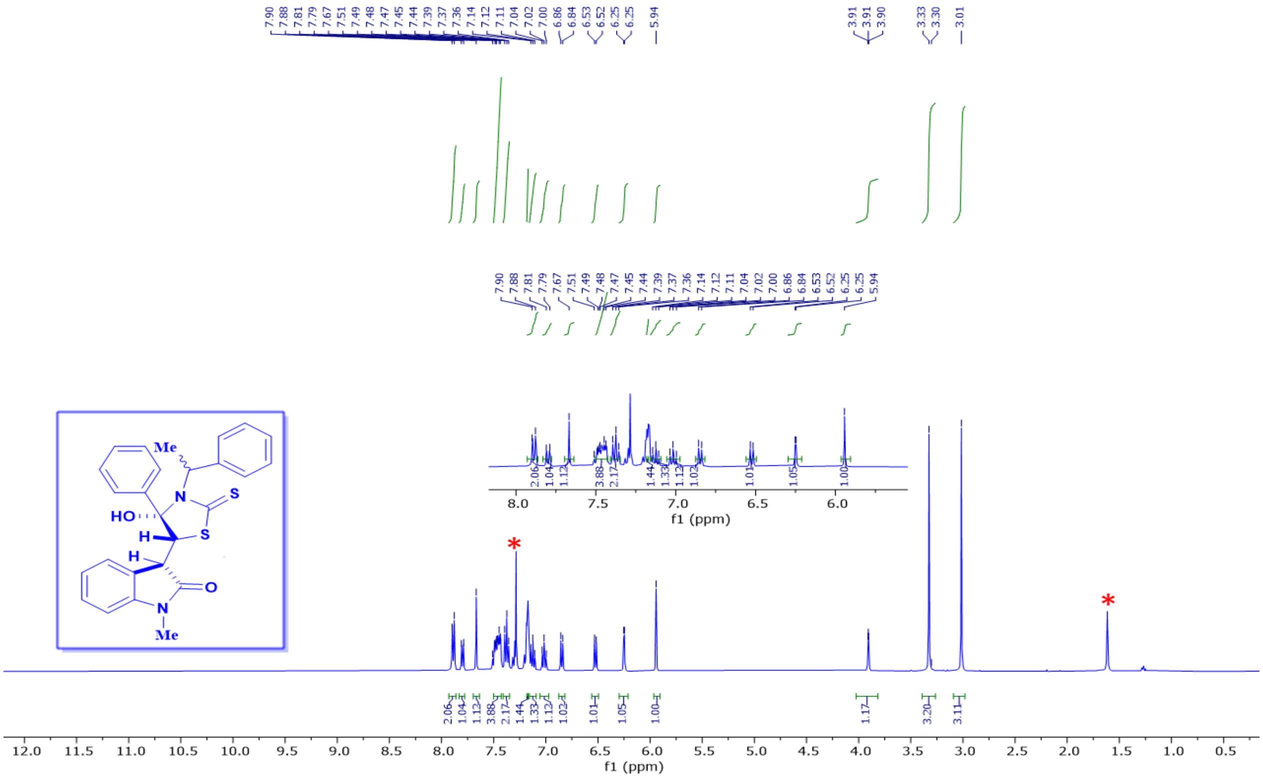

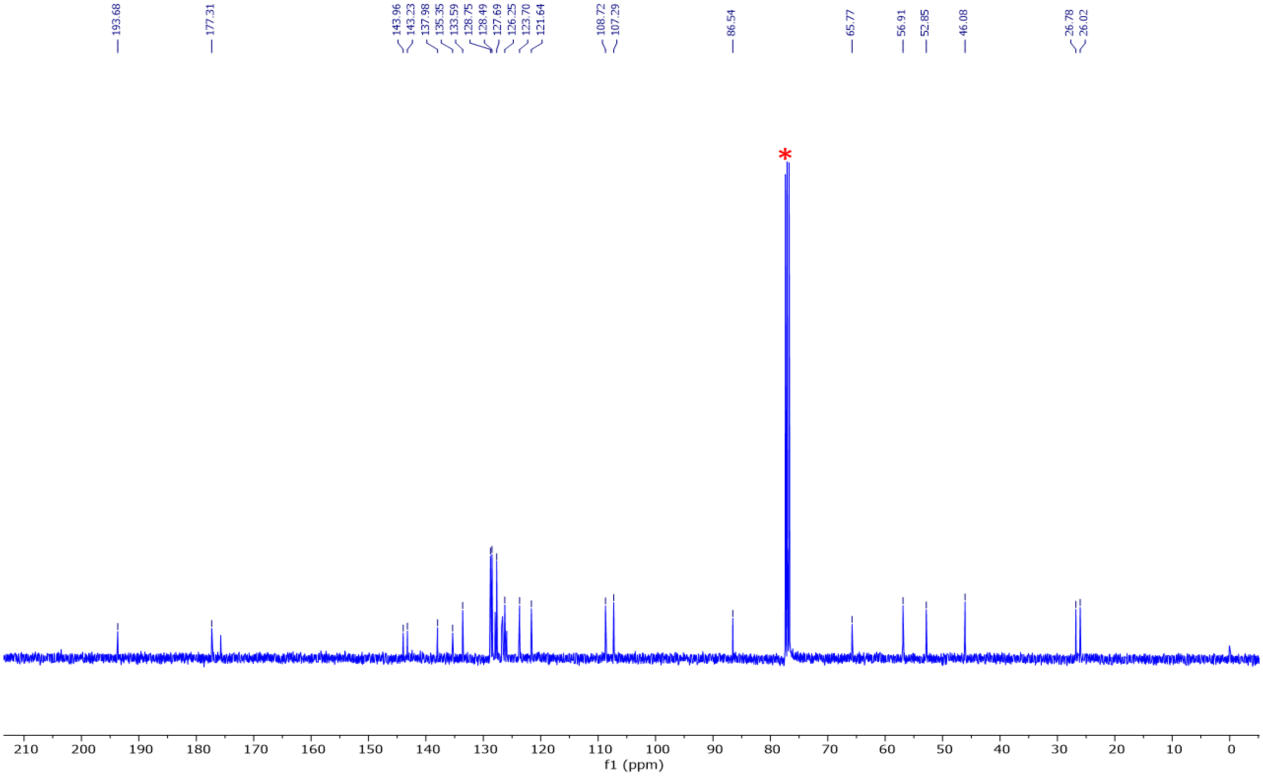

^{1}H NMR (500 MHz, $CDCl_3$), ^{13}C NMR (125 MHz, $CDCl_3$)

1-(1-allyl-2-oxoindolin-3-yl)-2-oxo-2-phenylethyl dimethylcarbamodithioate (4a)

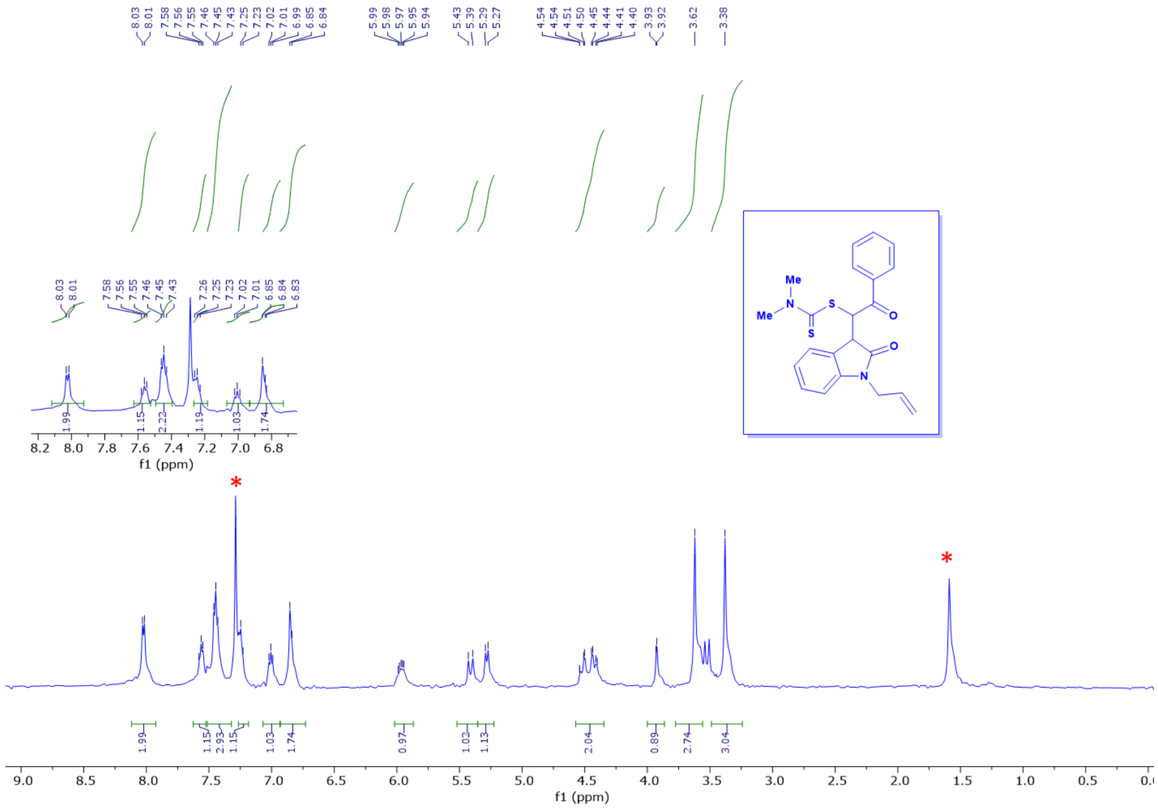

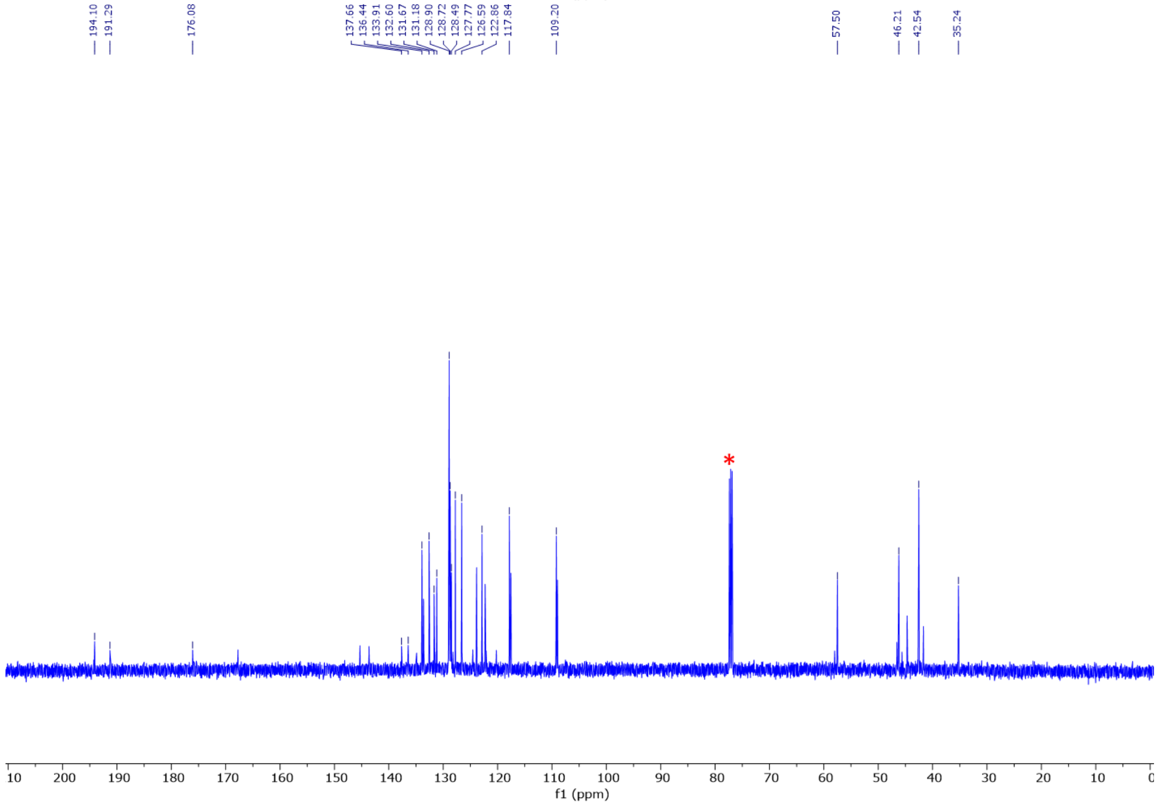

^{1}H NMR (500 MHz, $CDCl_3$), ^{13}C NMR (125 MHz, $CDCl_3$)

1-(1-benzyl-2-oxoindolin-3-yl)-2-oxo-2-phenylethyl dimethylcarbamodithioate (4b)

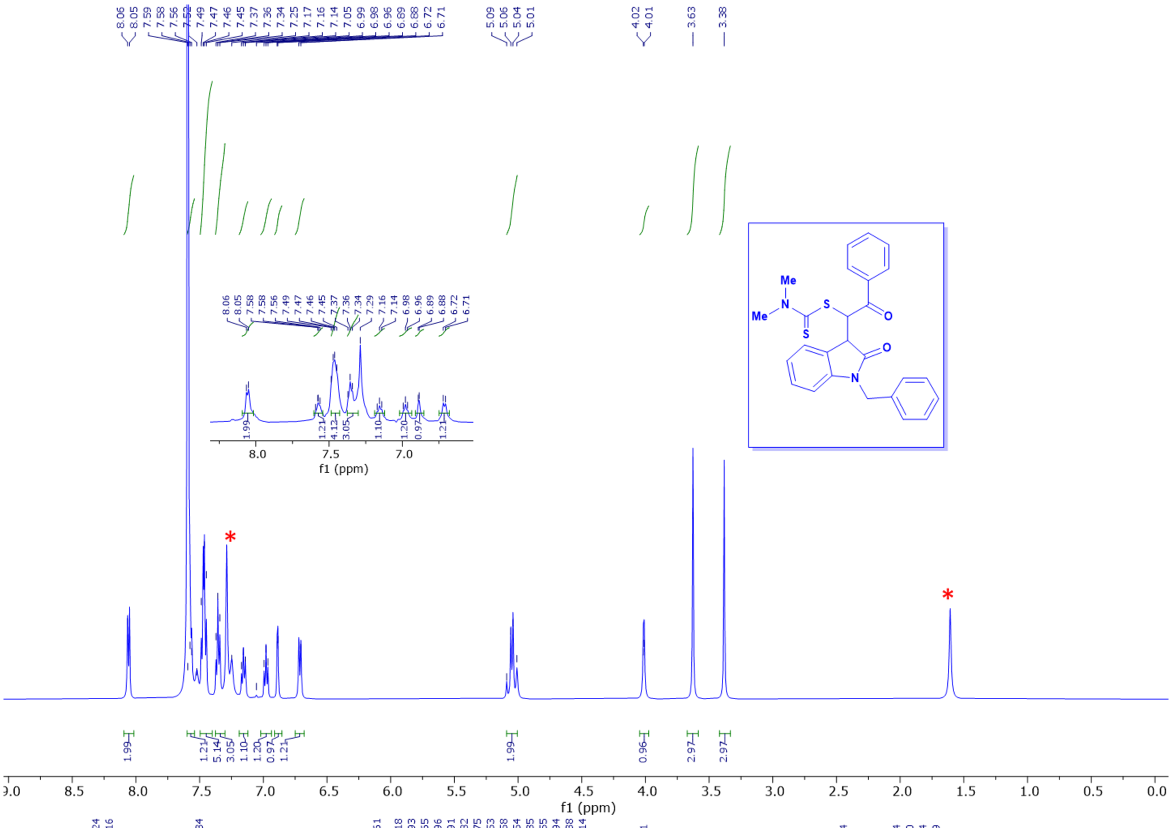

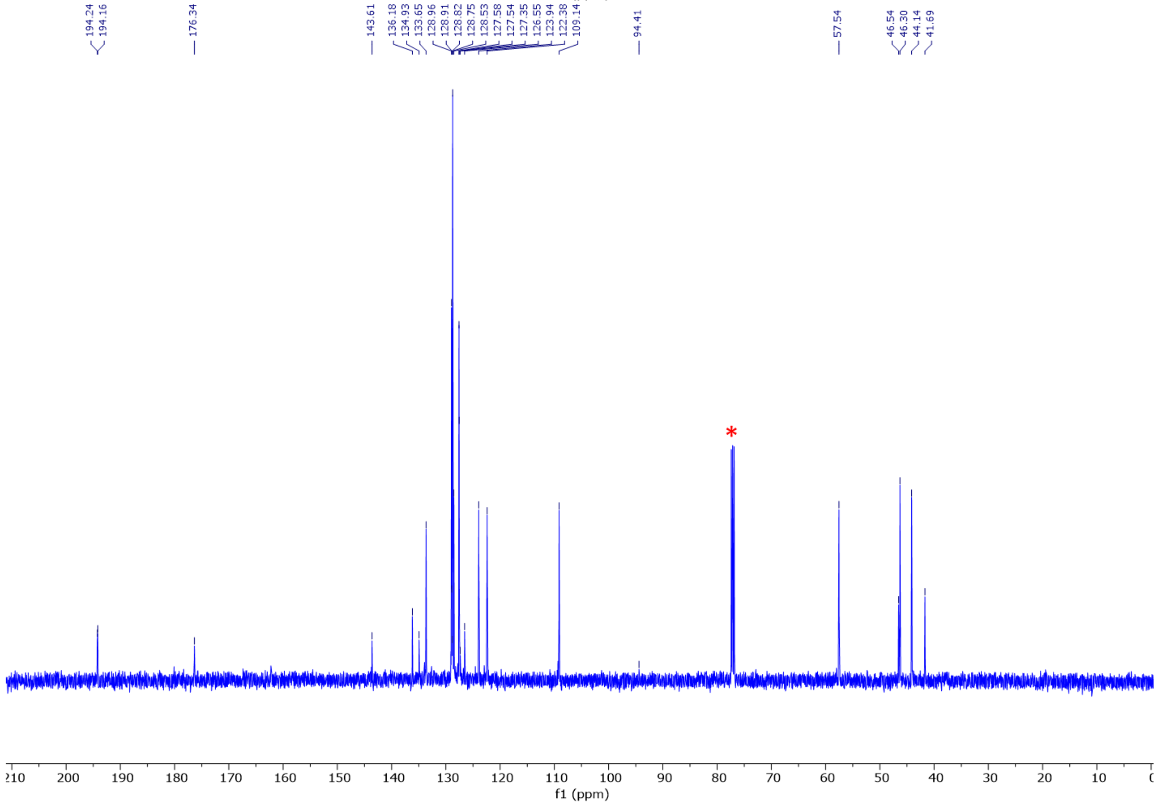

^{1}H NMR (500 MHz, $CDCl_3$), ^{13}C NMR (125 MHz, $CDCl_3$)

1-(1-allyl-5-chloro-2-oxoindolin-3-yl)-2-oxo-2-phenylethyl dimethylcarbamodithioate (4c)

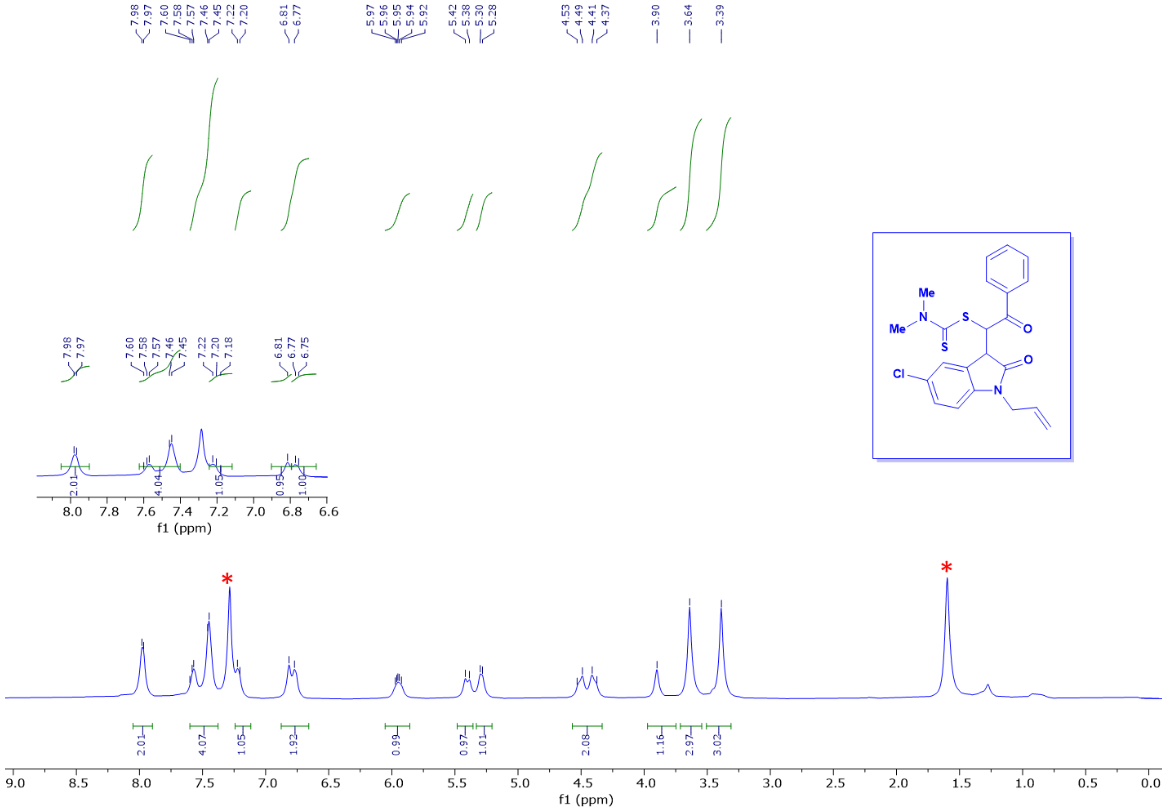

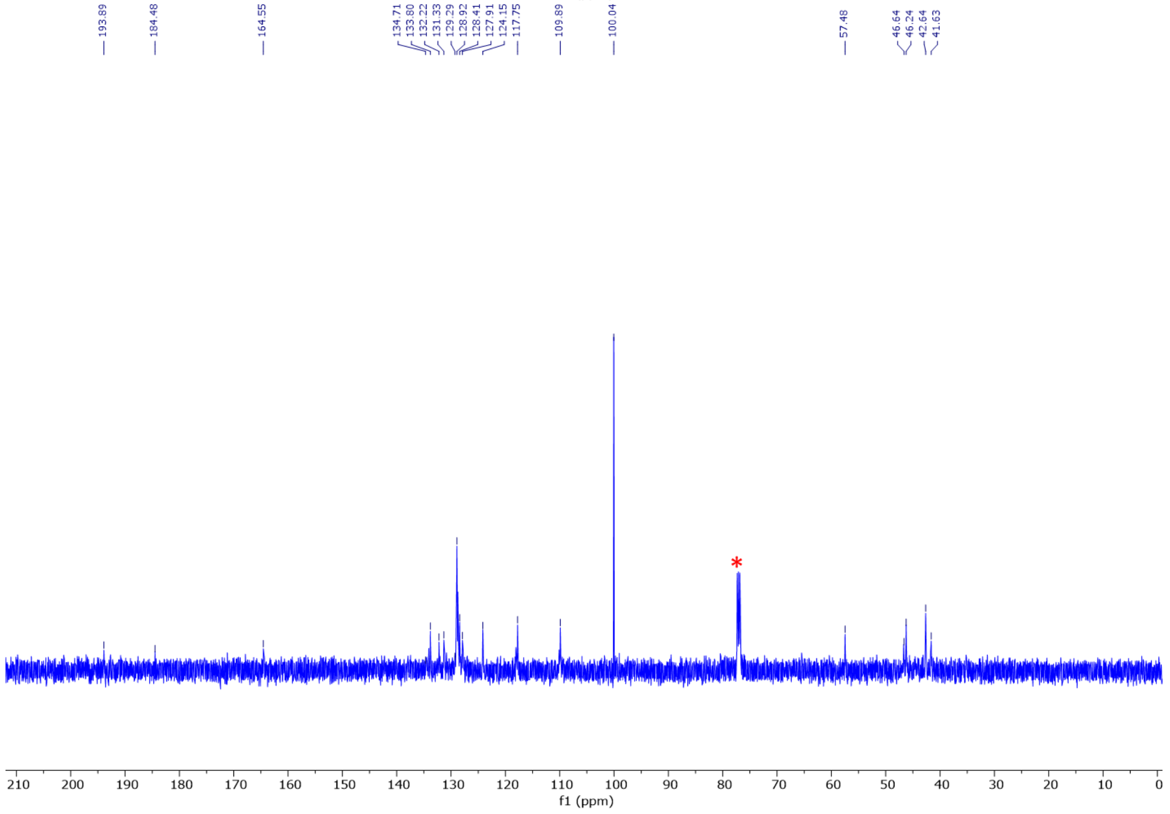

^{1}H NMR (500 MHz, $CDCl_3$), ^{13}C NMR (125 MHz, $CDCl_3$)

1-(1-benzyl-5-chloro-2-oxoindolin-3-yl)-2-oxo-2-phenylethyl dimethylcarbamodithioate (4d)

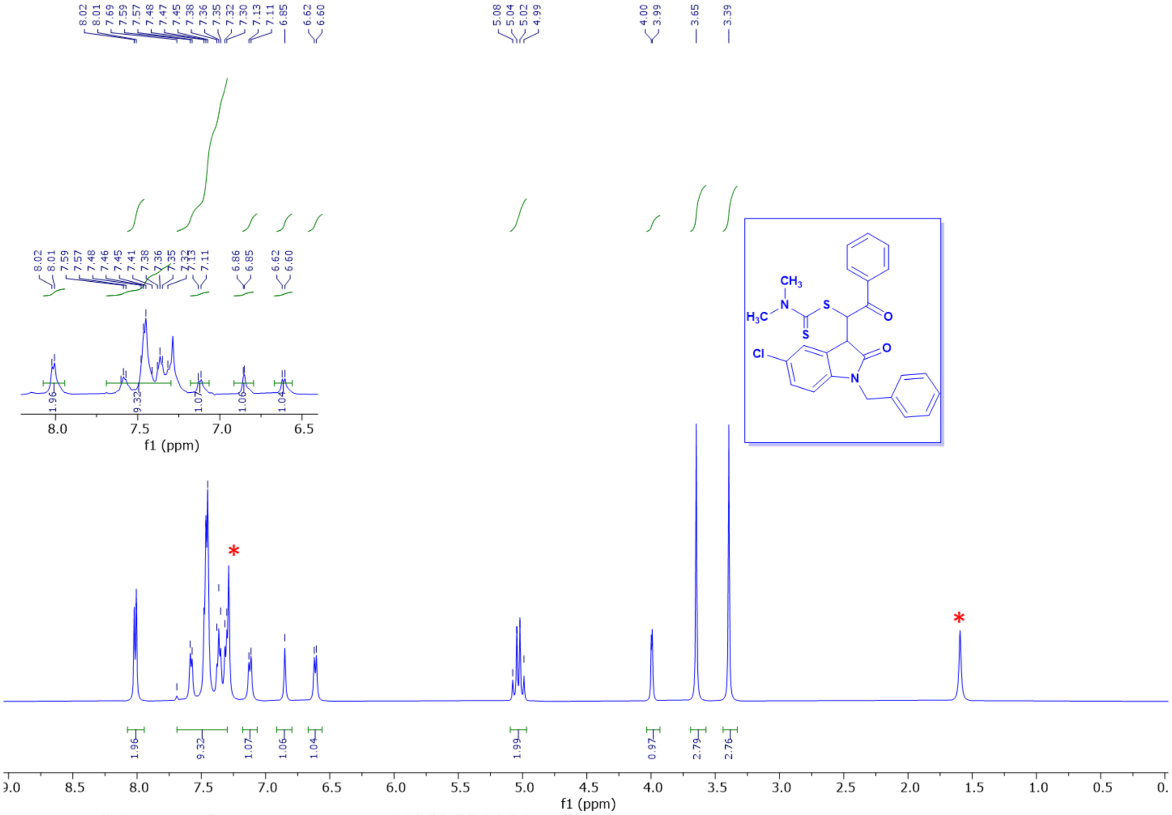

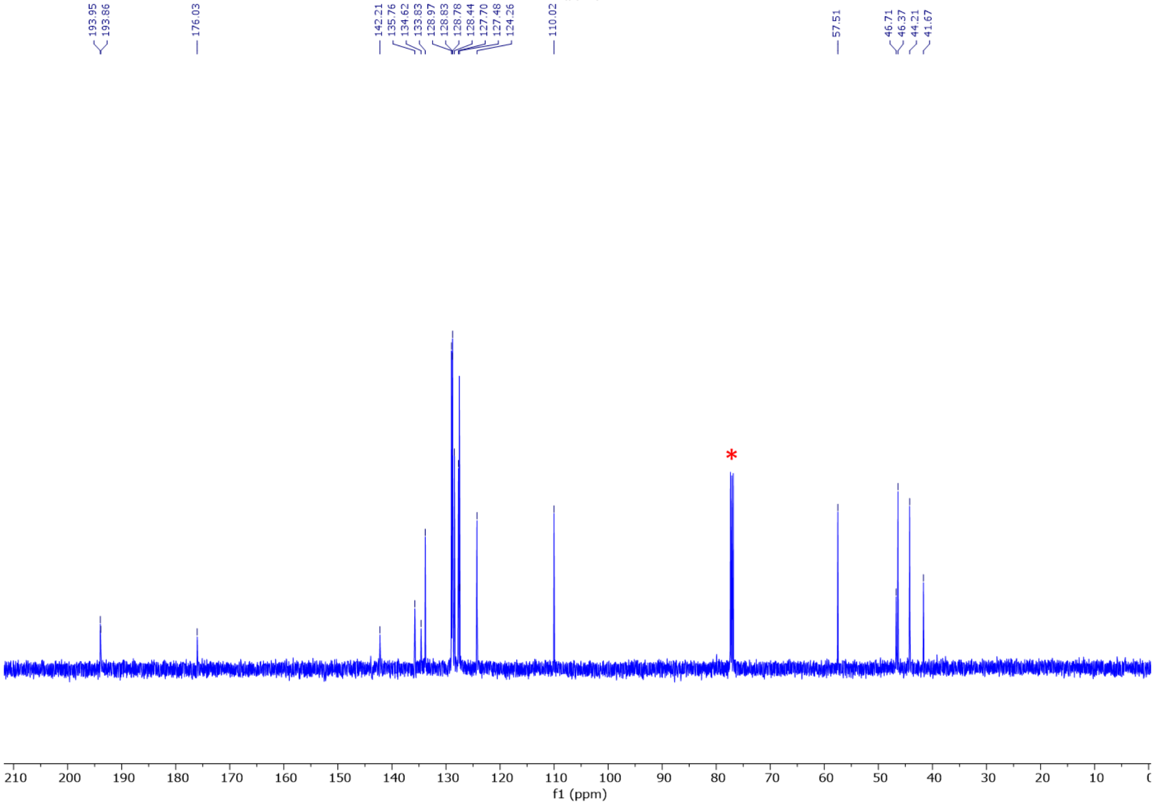

^{1}H NMR (500 MHz, DMSO-d6), ^{13}C NMR (125 MHz, DMSO-d6)

1,1''-diallyl-2'-benzoyl-4'-hydroxy-5'-methoxy-4'-phenyldispiro[indoline-3,1'-cyclopentane-3',3''-indoline]-2,2''-dione (5a)

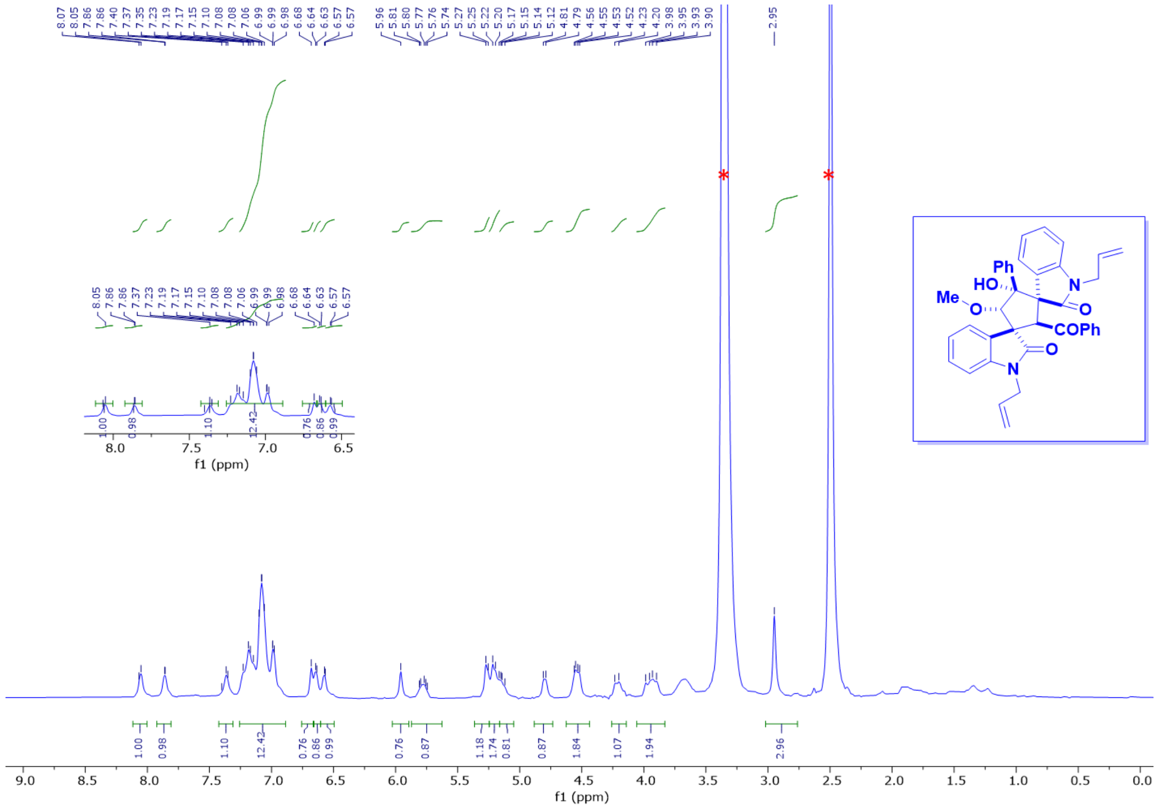

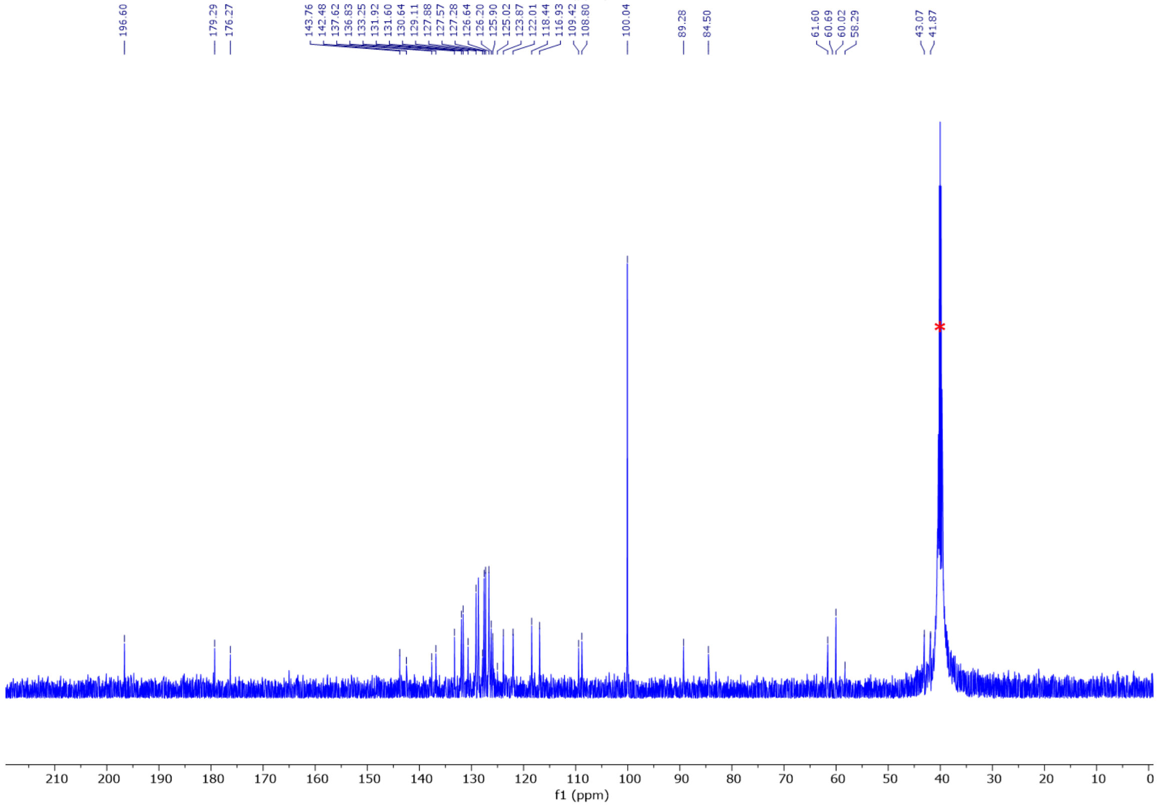

^{1}H NMR (500 MHz, DMSO-d$_6$), ^{13}C NMR (125 MHz, DMSO-d$_6$)

2'-benzoyl-4'-hydroxy-1,1"-dimethyl-5'-(octyloxy)-4'-phenyldispiro[indoline-3,1'-cyclopentane-3',3"-indoline]-2,2"-dione (5b)

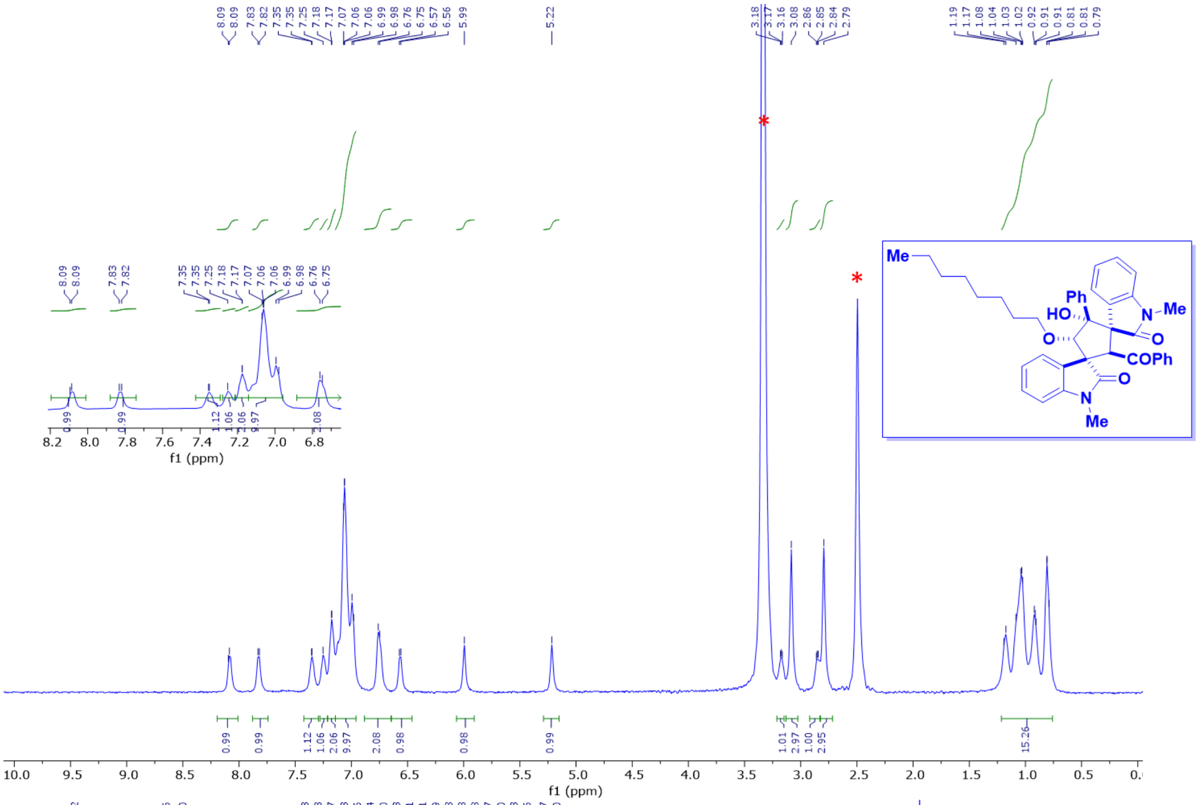

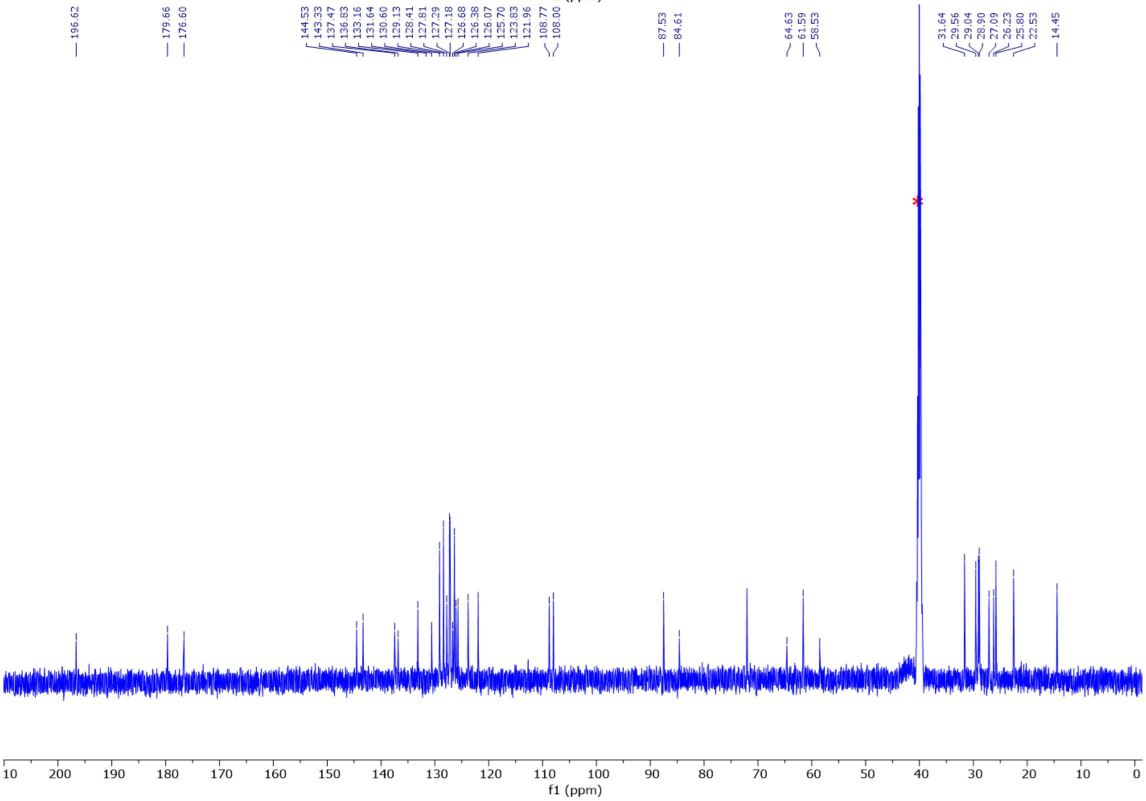

^{1}H NMR (500 MHz, DMSO-d_6), ^{13}C NMR (125 MHz, DMSO-d_6)

1,1"-diallyl-2'-benzoyl-4'-hydroxy-4'-phenyl-5'-propoxydispiro[indoline-3,1'-cyclopentane-3',3"-indoline]-2,2"-dione (5c)

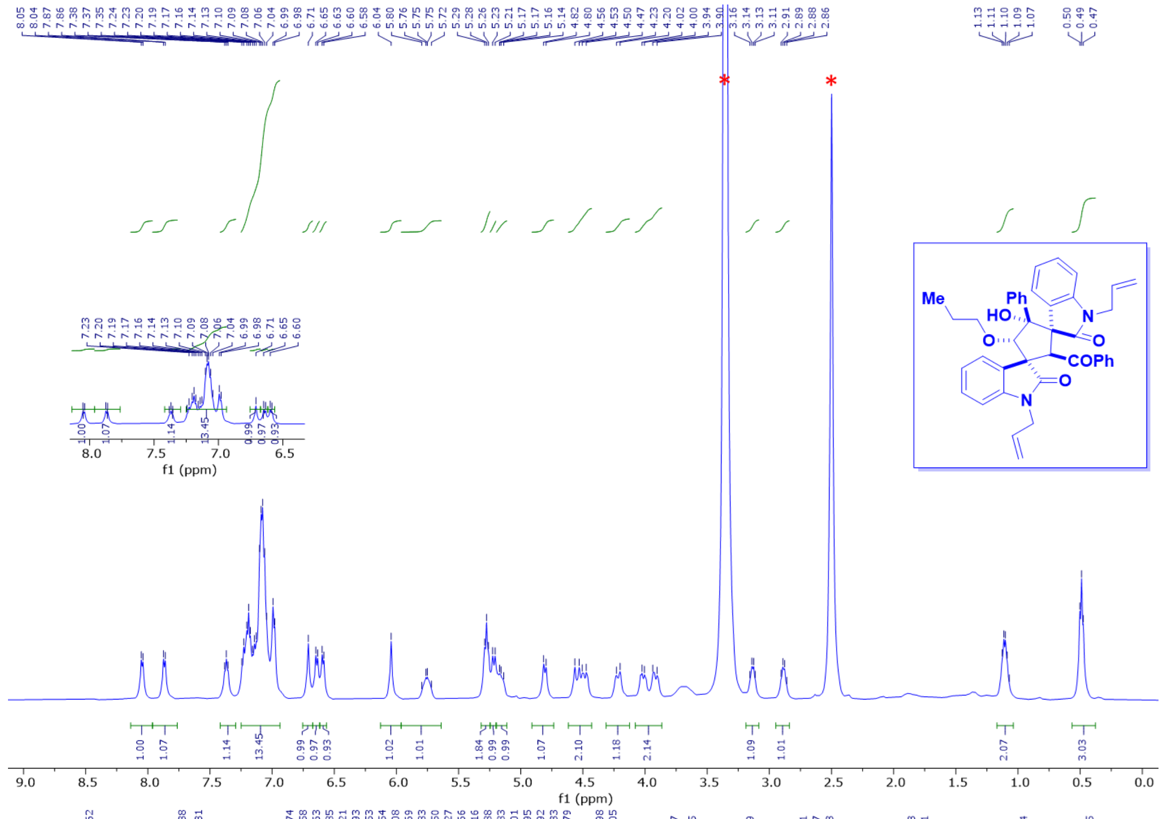

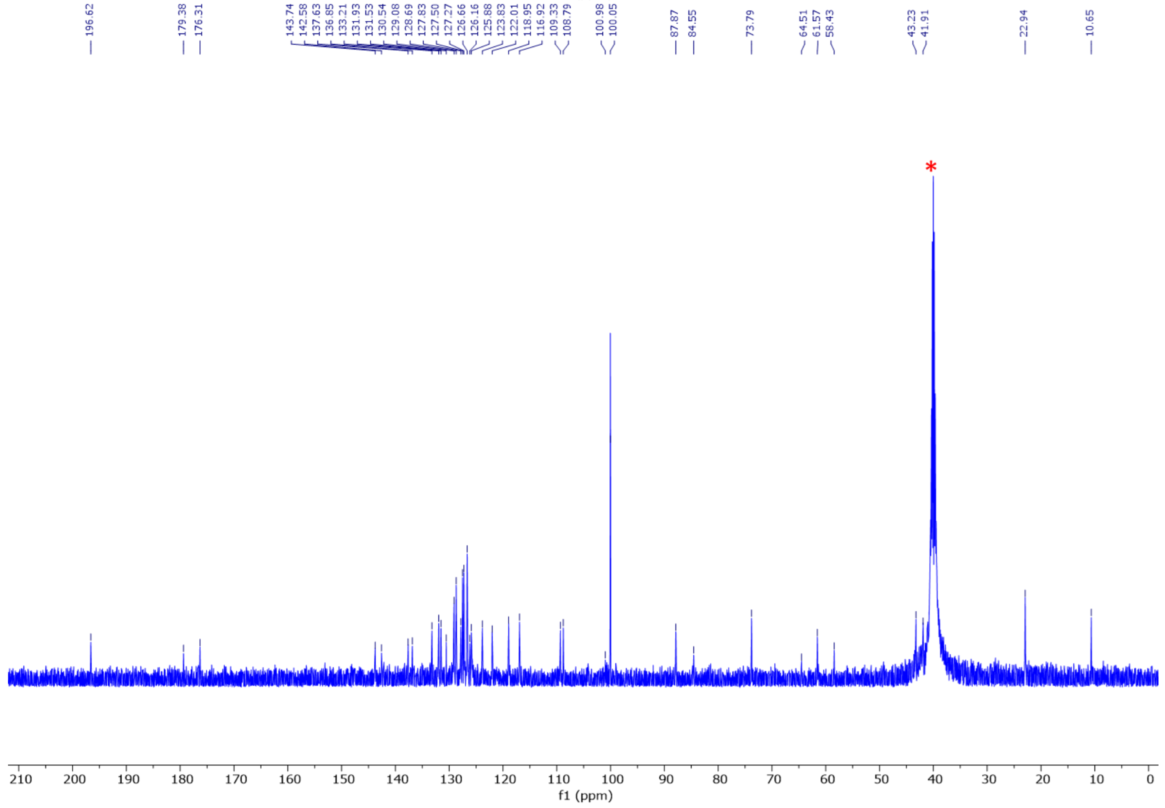

^{1}H NMR (500 MHz, DMSO-d6), ^{13}C NMR (125 MHz, DMSO-d6)

4'-hydroxy-2'-methyl-4'-phenyl-5'-propoxy-1,1"-dipropyldispiro[indoline-3,1'-cyclopentane-3',3"-indoline]-2,2"-dione (5d)

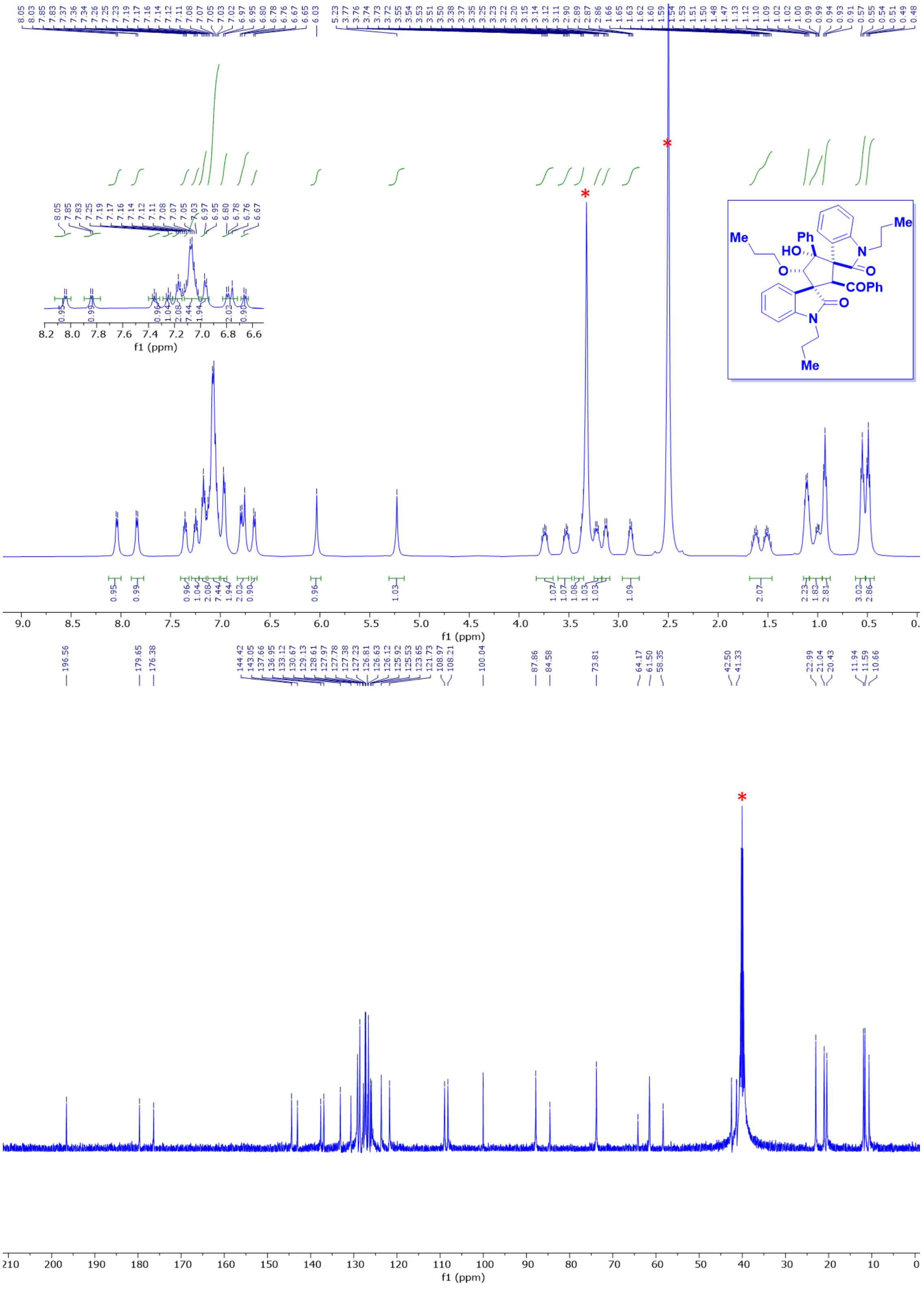

^{1}H NMR (500 MHz, DMSO-d6), ^{13}C NMR (125 MHz, DMSO-d6)

2'-benzoyl-1,1"-di(but-2-en-1-yl)-4'-hydroxy-5'-methoxy-4'-phenyldispiro[indoline-3,1'-cyclopentane-3',3"-indoline]-2,2"-dione (5e)

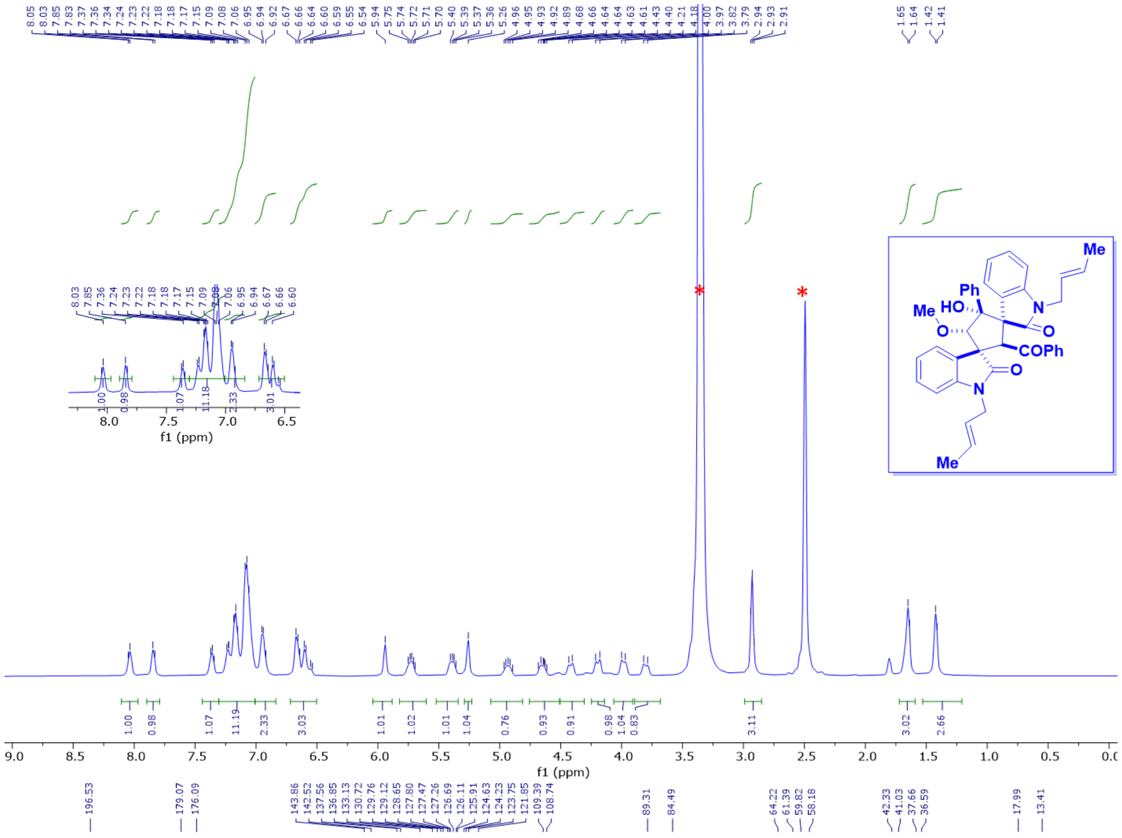

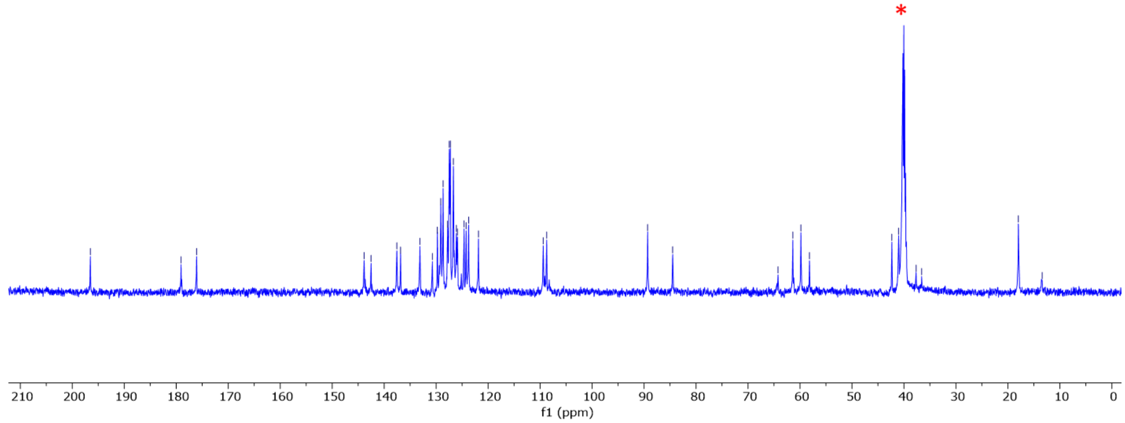

^{1}H NMR (500 MHz, DMSO-d6), ^{13}C NMR (125 MHz, DMSO-d6)

2'-benzoyl-1,1"-di((E)-but-2-en-1-yl)-5'-ethoxy-4'-hydroxy-4'-phenyldispiro[indoline-3,1'-cyclopentane-3',3"-indoline]-2,2"-dione (5f)

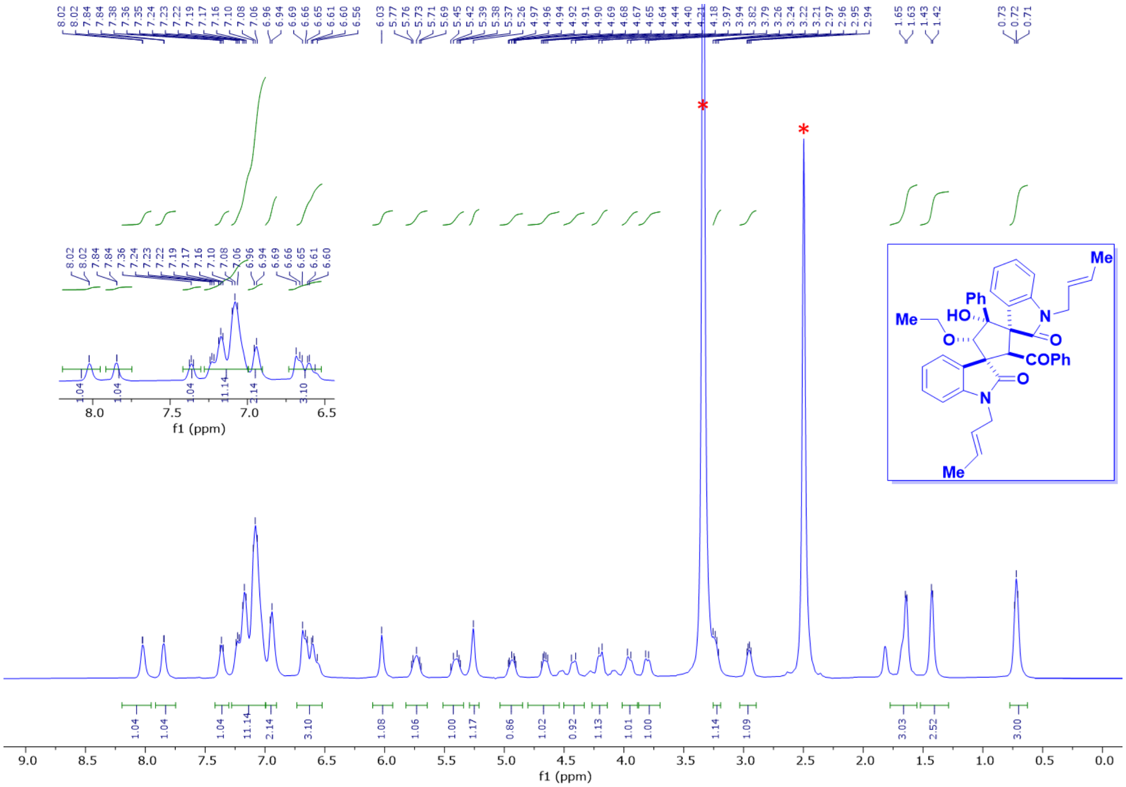

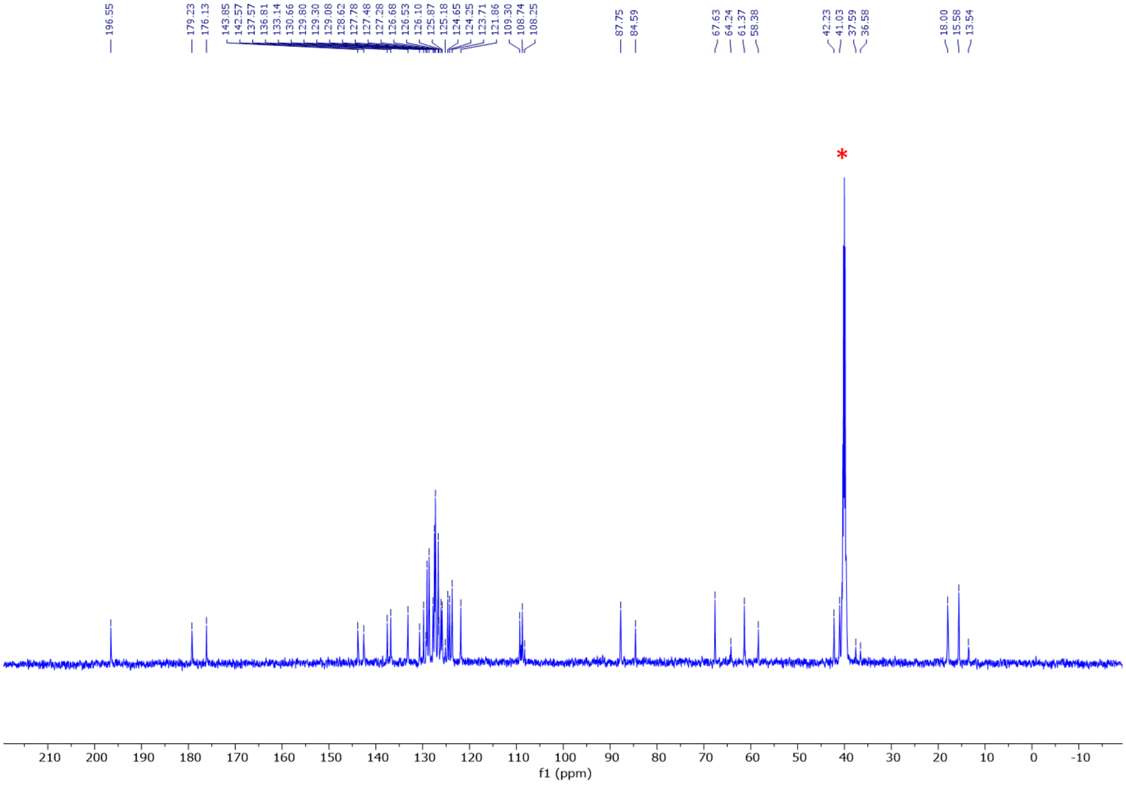

^{1}H NMR (500 MHz, DMSO-d_6), ^{13}C NMR (125 MHz, DMSO-d_6)

2'-benzoyl-5'-ethoxy-4'-hydroxy-4'-phenyl-1,1''-dimethyldispiro[indoline-3,1'-cyclopentane-3',3''-indoline]-2,2''-dione (5g)

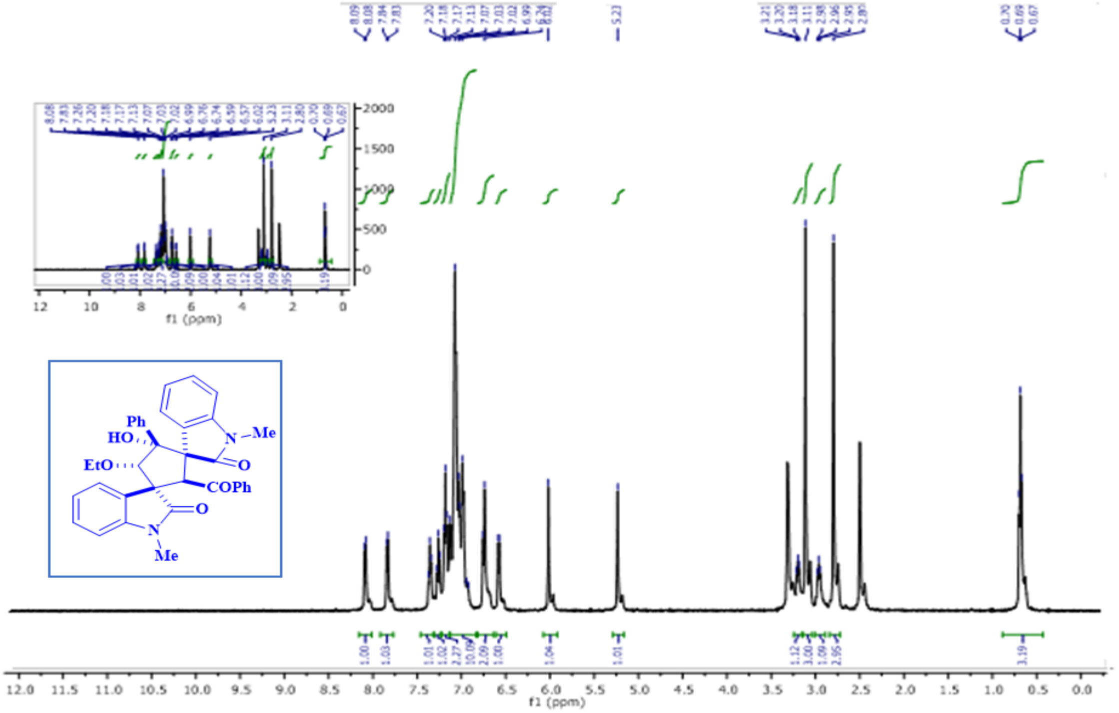

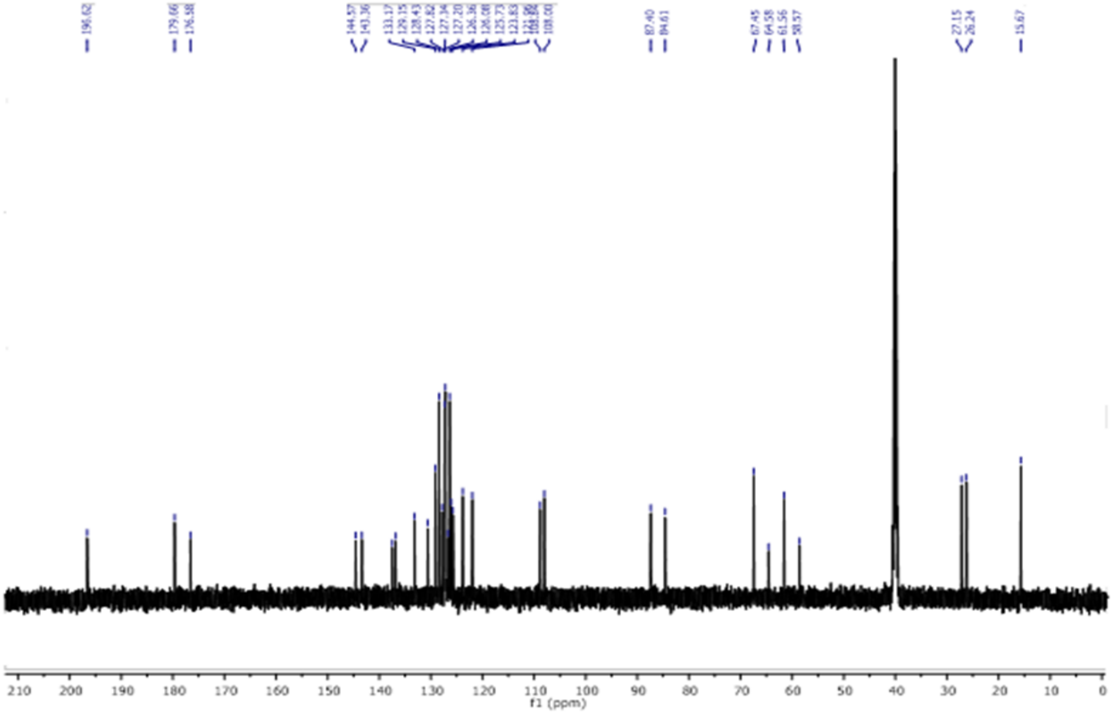

5. D_2O exchange of Product 3d

^{1}H NMR (500 MHz, $CDCl_3$), ^{13}C NMR (125 MHz, $CDCl_3$)

5-chloro-3-(4-hydroxy-3-methyl-4-phenyl-2-thioxothiazolidin-5-yl)-1-propylindolin-2-one (3d)

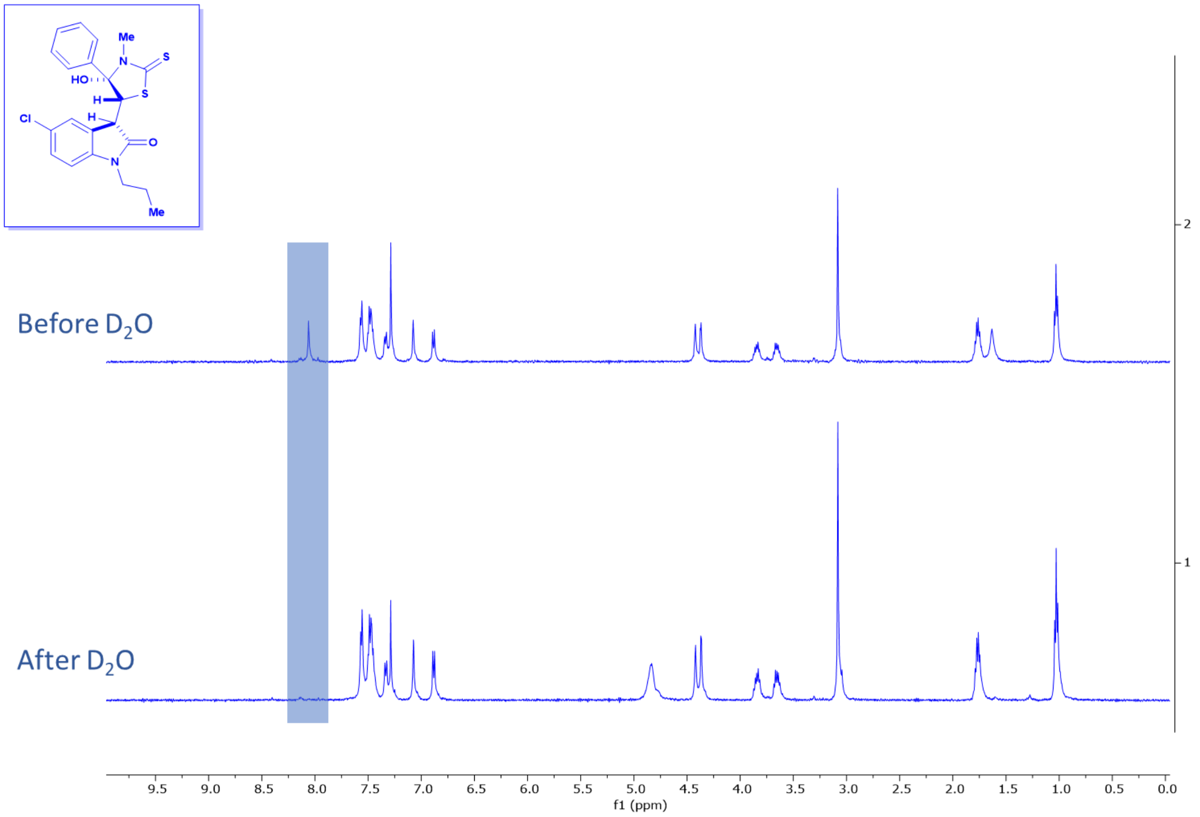

6. X-ray crystallography data

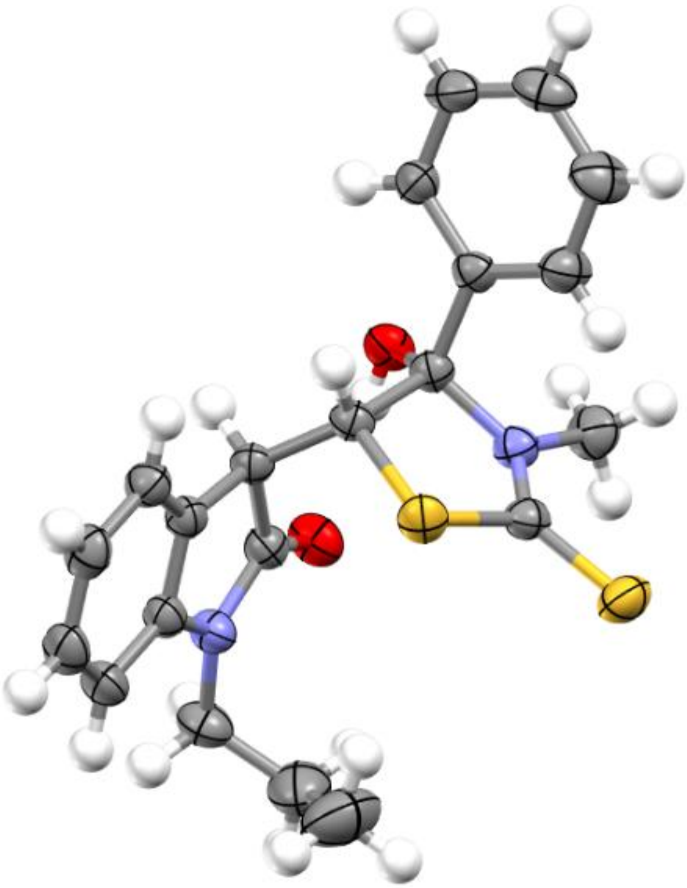

Figure S1. Single-crystal structure of 3c (ellipsoid contour % probability levels: 40).

7. Table S1. Crystal data and structure refinement for the compound 3c.

Identification code	1	
Empirical formula	C21 H22 N2 O2 S2	
Formula weight	398.52	
Temperature	293(2) K	
Wavelength	0.71073 Å	
Crystal system	Monoclinic	
Space group	$P\ 2_1/c$	
Unit cell dimensions	a = 10.921(2) Å	α= 90°
	b = 11.077(2) Å	β= 98.13(3) °
	c = 16.348(3) Å	γ = 90°
Volume	1957.8(6) $Å^3$	
Z	4	
Density (calculated)	1.352 Mg/m^3	
Absorption coefficient	0.291 mm^{-1}	
F(000)	840	
Crystal size	0.58 x 0.45 x 0.42 mm^3	
Theta range for data collection	2.228 to 25.000°.	
Index ranges	-12<=h<=12, -13<=k<=13, -19<=l<=19	
Reflections collected	11795	
Independent reflections	3370 [R(int) = 0.0435]	
Completeness to theta = 25.000°	97.9 %	
Absorption correction	Semi-empirical from equivalents	
Max. and min. transmission	1.04808 and 0.97099	
Refinement method	Full-matrix least-squares on F^2	
Data / restraints / parameters	3370 / 0 / 247	
Goodness-of-fit on F^2	1.086	
Final R indices [I>2sigma(I)]	R1 = 0.0455, wR2 = 0.1073	
R indices (all data)	R1 = 0.0499, wR2 = 0.1104	
Largest diff. peak and hole	0.311 and -0.249 e.$Å^{-3}$	

Table S2. Bond lengths [Å] and angles [°] for the compound.

S(001)-C(022)	1.819(2)
S(001)-C(026)	1.749(2)
S(004)-C(026)	1.659(2)
N(005)-C(021)	1.486(3)
N(005)-C(026)	1.337(3)
N(005)-C(041)	1.464(3)
O(008)-H(008)	0.8200
O(008)-C(021)	1.398(2)
O(010)-C(028)	1.238(3)
C(013)-C(021)	1.525(3)
C(013)-C(027)	1.391(3)
C(013)-C(038)	1.380(3)
C(014)-C(016)	1.379(3)
C(014)-C(029)	1.390(3)
C(014)-C(042)	1.515(3)
C(016)-H(016)	0.9300
C(016)-C(043)	1.398(3)
C(021)-C(022)	1.571(3)
C(022)-H(022)	0.9800
C(022)-C(042)	1.538(3)
N(023)-C(028)	1.355(3)
N(023)-C(029)	1.418(3)
N(023)-C(048)	1.462(3)
C(027)-H(027)	0.9300
C(027)-C(045)	1.386(3)
C(028)-C(042)	1.524(3)
C(029)-C(033)	1.389(3)
C(033)-H(033)	0.9300
C(033)-C(037)	1.384(4)
C(037)-H(037)	0.9300
C(037)-C(043)	1.380(4)
C(038)-H(038)	0.9300
C(038)-C(050)	1.394(4)
C(041)-H(04A)	0.9600

C(041)-H(04B)	0.9600
C(041)-H(04C)	0.9600
C(042)-H(042)	0.9800
C(043)-H(043)	0.9300
C(045)-H(045)	0.9300
C(045)-C(046)	1.372(4)
C(046)-H(046)	0.9300
C(046)-C(050)	1.367(4)
C(048)-H(04D)	0.9700
C(048)-H(04E)	0.9700
C(048)-C(051)	1.482(4)
C(050)-H(050)	0.9300
C(051)-H(05A)	0.9700
C(051)-H(05B)	0.9700
C(051)-C(1)	1.459(5)
C(1)-H(1A)	0.9600
C(1)-H(1B)	0.9600
C(1)-H(1C)	0.9600
C(026)-S(001)-C(022)	94.20(10)
C(026)-N(005)-C(021)	117.73(17)
C(026)-N(005)-C(041)	121.89(19)
C(041)-N(005)-C(021)	119.53(17)
C(021)-O(008)-H(008)	109.5
C(027)-C(013)-C(021)	118.96(19)
C(038)-C(013)-C(021)	121.94(19)
C(038)-C(013)-C(027)	119.0(2)
C(016)-C(014)-C(029)	120.0(2)
C(016)-C(014)-C(042)	131.6(2)
C(029)-C(014)-C(042)	108.32(18)
C(014)-C(016)-H(016)	120.5
C(014)-C(016)-C(043)	118.9(2)
C(043)-C(016)-H(016)	120.5
N(005)-C(021)-C(013)	110.24(17)
N(005)-C(021)-C(022)	106.20(16)
O(008)-C(021)-N(005)	110.18(16)

O(008)-C(021)-C(013)	107.57(16)
O(008)-C(021)-C(022)	112.91(16)
C(013)-C(021)-C(022)	109.75(16)
S(001)-C(022)-H(022)	107.2
C(021)-C(022)-S(001)	105.65(14)
C(021)-C(022)-H(022)	107.2
C(042)-C(022)-S(001)	111.41(14)
C(042)-C(022)-C(021)	117.56(17)
C(042)-C(022)-H(022)	107.2
C(028)-N(023)-C(029)	110.89(18)
C(028)-N(023)-C(048)	123.35(19)
C(029)-N(023)-C(048)	125.74(19)
S(004)-C(026)-S(001)	120.20(13)
N(005)-C(026)-S(001)	111.67(16)
N(005)-C(026)-S(004)	128.11(18)
C(013)-C(027)-H(027)	120.0
C(045)-C(027)-C(013)	120.0(2)
C(045)-C(027)-H(027)	120.0
O(010)-C(028)-N(023)	124.6(2)
O(010)-C(028)-C(042)	126.51(19)
N(023)-C(028)-C(042)	108.85(18)
C(014)-C(029)-N(023)	109.56(18)
C(033)-C(029)-C(014)	121.7(2)
C(033)-C(029)-N(023)	128.7(2)
C(029)-C(033)-H(033)	121.3
C(037)-C(033)-C(029)	117.4(2)
C(037)-C(033)-H(033)	121.3
C(033)-C(037)-H(037)	119.1
C(043)-C(037)-C(033)	121.8(2)
C(043)-C(037)-H(037)	119.1
C(013)-C(038)-H(038)	119.9
C(013)-C(038)-C(050)	120.2(2)
C(050)-C(038)-H(038)	119.9
N(005)-C(041)-H(04A)	109.5
N(005)-C(041)-H(04B)	109.5
N(005)-C(041)-H(04C)	109.5

H(04A)-C(041)-H(04B)	109.5
H(04A)-C(041)-H(04C)	109.5
H(04B)-C(041)-H(04C)	109.5
C(014)-C(042)-C(022)	112.38(17)
C(014)-C(042)-C(028)	102.32(16)
C(014)-C(042)-H(042)	108.8
C(022)-C(042)-H(042)	108.8
C(028)-C(042)-C(022)	115.30(17)
C(028)-C(042)-H(042)	108.8
C(016)-C(043)-H(043)	119.9
C(037)-C(043)-C(016)	120.1(2)
C(037)-C(043)-H(043)	119.9
C(027)-C(045)-H(045)	119.8
C(046)-C(045)-C(027)	120.5(2)
C(046)-C(045)-H(045)	119.8
C(045)-C(046)-H(046)	120.1
C(050)-C(046)-C(045)	119.9(2)
C(050)-C(046)-H(046)	120.1
N(023)-C(048)-H(04D)	108.7
N(023)-C(048)-H(04E)	108.7
N(023)-C(048)-C(051)	114.3(2)
H(04D)-C(048)-H(04E)	107.6
C(051)-C(048)-H(04D)	108.7
C(051)-C(048)-H(04E)	108.7
C(038)-C(050)-H(050)	119.8
C(046)-C(050)-C(038)	120.4(3)
C(046)-C(050)-H(050)	119.8
C(048)-C(051)-H(05A)	108.2
C(048)-C(051)-H(05B)	108.2
H(05A)-C(051)-H(05B)	107.3
C(1)-C(051)-C(048)	116.4(3)
C(1)-C(051)-H(05A)	108.2
C(1)-C(051)-H(05B)	108.2
C(051)-C(1)-H(1A)	109.5
C(051)-C(1)-H(1B)	109.5
C(051)-C(1)-H(1C)	109.5

H(1A)-C(1)-H(1B)	109.5
H(1A)-C(1)-H(1C)	109.5
H(1B)-C(1)-H(1C)	109.5

Symmetry transformations used to generate equivalent atoms:

Table S3. Torsion angles [°] for the compound.

S(001)-C(022)-C(042)-C(014)	53.0(2)
S(001)-C(022)-C(042)-C(028)	-63.8(2)
N(005)-C(021)-C(022)-S(001)	21.12(18)
N(005)-C(021)-C(022)-C(042)	-103.90(19)
O(008)-C(021)-C(022)-S(001)	141.97(15)
O(008)-C(021)-C(022)-C(042)	17.0(3)
O(010)-C(028)-C(042)-C(014)	-179.7(2)
O(010)-C(028)-C(042)-C(022)	-57.5(3)
C(013)-C(021)-C(022)-S(001)	-98.03(16)
C(013)-C(021)-C(022)-C(042)	136.95(18)
C(013)-C(027)-C(045)-C(046)	-0.9(4)
C(013)-C(038)-C(050)-C(046)	0.0(4)
C(014)-C(016)-C(043)-C(037)	-0.6(4)
C(014)-C(029)-C(033)-C(037)	-0.1(3)
C(016)-C(014)-C(029)-N(023)	-179.75(19)
C(016)-C(014)-C(029)-C(033)	0.6(3)
C(016)-C(014)-C(042)-C(022)	54.3(3)
C(016)-C(014)-C(042)-C(028)	178.5(2)
C(021)-N(005)-C(026)-S(001)	10.5(2)
C(021)-N(005)-C(026)-S(004)	-167.92(16)
C(021)-C(013)-C(027)-C(045)	179.1(2)
C(021)-C(013)-C(038)-C(050)	-178.6(2)
C(021)-C(022)-C(042)-C(014)	175.08(17)
C(021)-C(022)-C(042)-C(028)	58.3(2)
C(022)-S(001)-C(026)-S(004)	-178.00(14)
C(022)-S(001)-C(026)-N(005)	3.48(17)
N(023)-C(028)-C(042)-C(014)	2.3(2)
N(023)-C(028)-C(042)-C(022)	124.62(19)
N(023)-C(029)-C(033)-C(037)	-179.7(2)
N(023)-C(048)-C(051)-C(1)	52.9(4)
C(026)-S(001)-C(022)-C(021)	-14.61(15)
C(026)-S(001)-C(022)-C(042)	114.14(15)
C(026)-N(005)-C(021)-O(008)	-143.85(18)
C(026)-N(005)-C(021)-C(013)	97.6(2)

C(026)-N(005)-C(021)-C(022)	-21.3(2)
C(027)-C(013)-C(021)-N(005)	161.17(18)
C(027)-C(013)-C(021)-O(008)	41.0(2)
C(027)-C(013)-C(021)-C(022)	-82.2(2)
C(027)-C(013)-C(038)-C(050)	-1.3(4)
C(027)-C(045)-C(046)-C(050)	-0.5(4)
C(028)-N(023)-C(029)-C(014)	1.3(2)
C(028)-N(023)-C(029)-C(033)	-179.1(2)
C(028)-N(023)-C(048)-C(051)	74.9(3)
C(029)-C(014)-C(016)-C(043)	-0.2(3)
C(029)-C(014)-C(042)-C(022)	-125.82(19)
C(029)-C(014)-C(042)-C(028)	-1.6(2)
C(029)-N(023)-C(028)-O(010)	179.7(2)
C(029)-N(023)-C(028)-C(042)	-2.3(2)
C(029)-N(023)-C(048)-C(051)	-106.2(3)
C(029)-C(033)-C(037)-C(043)	-0.8(4)
C(033)-C(037)-C(043)-C(016)	1.1(4)
C(038)-C(013)-C(021)-N(005)	-21.5(3)
C(038)-C(013)-C(021)-O(008)	-141.7(2)
C(038)-C(013)-C(021)-C(022)	95.1(2)
C(038)-C(013)-C(027)-C(045)	1.7(3)
C(041)-N(005)-C(021)-O(008)	46.5(3)
C(041)-N(005)-C(021)-C(013)	-72.0(2)
C(041)-N(005)-C(021)-C(022)	169.13(19)
C(041)-N(005)-C(026)-S(001)	179.81(18)
C(041)-N(005)-C(026)-S(004)	1.4(3)
C(042)-C(014)-C(016)-C(043)	179.7(2)
C(042)-C(014)-C(029)-N(023)	0.3(2)
C(042)-C(014)-C(029)-C(033)	-179.3(2)
C(045)-C(046)-C(050)-C(038)	0.9(4)
C(048)-N(023)-C(028)-O(010)	-1.2(3)
C(048)-N(023)-C(028)-C(042)	176.8(2)
C(048)-N(023)-C(029)-C(014)	-177.8(2)
C(048)-N(023)-C(029)-C(033)	1.8(4)
